# Glacier influence shapes the genomic architecture of the downstream aquatic microbiome

**DOI:** 10.1093/ismeco/ycaf076

**Published:** 2025-05-14

**Authors:** Massimo Bourquin, Hannes Peter, Grégoire Michoud, Aileen Geers, The Vanishing Glaciers Field Team, Susheel Bhanu Busi, Tom Ian Battin

**Affiliations:** River Ecosystems Laboratory, Alpine and Polar Environmental Research Center, Ecole Polytechnique Fédérale de Lausanne, EPFL, 1951 Sion, Switzerland; River Ecosystems Laboratory, Alpine and Polar Environmental Research Center, Ecole Polytechnique Fédérale de Lausanne, EPFL, 1951 Sion, Switzerland; River Ecosystems Laboratory, Alpine and Polar Environmental Research Center, Ecole Polytechnique Fédérale de Lausanne, EPFL, 1951 Sion, Switzerland; River Ecosystems Laboratory, Alpine and Polar Environmental Research Center, Ecole Polytechnique Fédérale de Lausanne, EPFL, 1951 Sion, Switzerland; UK Centre for Ecology and Hydrology, Wallingford, OX10 8BB, United Kingdom; River Ecosystems Laboratory, Alpine and Polar Environmental Research Center, Ecole Polytechnique Fédérale de Lausanne, EPFL, 1951 Sion, Switzerland

**Keywords:** phylogenetics, glacier-fed streams, cryosphere, metagenomics, genomic architecture, microbial genomics, Gammaproteobacteria

## Abstract

The factors and processes that shape microbial genomes and determine the success of microbes in different environments have long attracted scientific interest. Here, leveraging 2855 metagenome-assembled genomes sampled by the *Vanishing Glacier Project* from glacier-fed streams (GFSs), we shed light on the genomic architecture of the benthic microbiome in these harsh ecosystems—now vanishing because of climate change. Owing to glacial influence, the GFS benthic habitat is unstable, notoriously cold, and ultra-oligotrophic. Along gradients of glacial influence and concomitant variation in benthic algal biomass across 149 GFSs draining Earth’s major mountain ranges, we show how genomes of GFS bacteria vary in terms of size, coding density, gene redundancy, and translational machinery. We develop a novel, phylogeny-rooted analytical framework that allows pinpointing the phylogenetic depth at which patterns in genomic trends occur. These analyses reveal both deep- and shallow-rooting phylogenetic patterns in genomic features associated with key GFS taxa and functional potential relevant to live in these ecosystems. Additionally, we highlight the role of several clades of Gammaproteobacteria in shaping community-level genomic architecture. Our work shows how genome architecture is shaped by selective environmental constraints in an extreme environment. These insights are important as they reveal putatively important adaptations to the GFS environment which is now changing at rapid pace due to climate change.

## Introduction

Bacterial genomes exhibit substantial variation in size and complexity [1, 2] and are shaped by processes including genetic drift [2, 3], selection by environmental constraints such as oligotrophy and symbiotic interactions that facilitate gene loss [1, 4]. Among the factors that shape bacterial genome architecture, environmental drivers related to genome size variation have attracted most attention. For instance, thermophilic microbes thriving in hot springs often possess small genomes [5], whereas psychrophilic microbes in cryospheric environments tend to have larger genomes [6–8]. Increased genome size has also been associated with the need to maintain a broad functional repertoire to cope with fluctuating environmental conditions [9–11]. In addition to habitat characteristics such as temperature and nutrient availability, microbial lifestyle—such as free-living pelagic versus attached forms—have also been associated with genome size variation [12–14]. However, other genomic features, such as variation in guanine-cytosine (GC) content, gene redundancy or the translational machinery have received less attention, particularly for environmental bacteria. Here, we analyse metagenomic and environmental data from glacier-fed streams (GFSs) and investigate relationships between glacial influence and variation in genomic features of the benthic microbiome.

Owing to the direct influence of glaciers [15], GFSs are ultra-oligotrophic, cold, and unstable environments, yet they harbor diverse microbial communities [16–18]. In GFSs, bacteria forming biofilms attached to sedimentary surfaces dominate microbial life, where they orchestrate important ecosystem functions [19–21]. These communities are shaped by selective environmental conditions, which is reflected by deterministic community assembly and elevated microdiversity [22, 23]. Yet, how the environmental characteristics shape the genomic architecture of GFS bacteria remains unknown. In the light of ongoing climate change and glacier recession [24], better understanding genomic trends along environmental factors is however important, as genome architecture underpins the diversity, distribution, and metabolic versatility of bacteria [25, 26].

Glacier meltwaters are oligotrophic, which may favor genome streamlining and low GC content, as has been observed in other nutrient-limited environments. GFS streamwater is often turbid due to high loads of fine suspended sediments [15, 27], which reduces light available for primary producers (i.e. benthic algae) and further aggravates resource limitation for heterotrophic bacteria [28]. In line with this, smaller average genome sizes have been reported from GFSs compared tributary streams that are not under glacial influence, albeit draining the same proglacial floodplains [29].

On the other hand, efficient stress response, abundant mobile genetic elements, translational flexibility and genome plasticity have been related to larger genomes of bacteria in cryospheric ecosystems [30]. In GFSs, rapid variation in flow and sediment loads and low streamwater temperatures may thus promote larger genomes [5]. Moreover, GFS bacteria thrive during windows of opportunity, which mainly arise in spring and autumn when nutrients and light are available and streamflow is moderate [19, 31, 32]. Similarly, bacteria with larger genomes and an expanded functional and regulatory repertoire thrive in in pelagic environments where rapid nutrient influx or depletion can occur [33].

Here, we consider genomic traits prevalent under high glacial influence to reflect selective pressures and thus as being indicative of adaptations to GFS conditions. This perspective is based on the idea that genomic features conferring fitness advantages—such as metabolic efficiency—become prevalent under strong selective constraints, while at the same time being shaped by the need to maintain sufficient functional flexibility to cope with environmental fluctuation [1]. Given the close relationship between genomic traits and evolutionary history, phylogenetic context is critical for interpreting variation in genomic features [34]. To this end, we establish a novel analytical framework for resolving the phylogenetic signatures of genomic features in GFSs. This null-model based framework allows us to determine the phylogenetic depth at which genomic traits exhibit a significant signal, allowing us to explore how genome variation is structured across phylogenetic scales and to disentangling the contribution of specific clades to community-level genomic patterns. Our work provides new insights into how environmental constraints shape bacterial genome architecture and contribute to the ecological success of specific clades in GFSs. These findings are particularly relevant in the context of climate change, as diminishing glacial influence may alter key selective constraints and potentially threaten microorganisms adapted to the GFS environment.

## Material and methods

### Glacier-fed stream sampling and environmental parameters

We sampled benthic biofilms (upper 5 cm of the streambed) from 149 GFSs in the European Alps, Scandinavian Mountains, Himalayas, Pamir and Tian Shan, Ecuadorian Andes, Southwest Greenland, Russian Caucasus, Rwenzori in Africa, and Southern Alps in New Zealand between January 2019 and July 2022. GFSs were sampled in spring or autumn during “windows of opportunity” when streamflow and streamwater turbidity are relatively low; this sampling strategy facilitates comparability between GFSs. We did not sample GFSs from heavily debris-covered and rock glaciers, and we avoided GFSs downstream of proglacial lakes, with debris flows, or tributaries in the reaches above the sampling sites. At each GFS, we sampled an upstream reach, as close as possible to the glacier snout, and a downstream reach. Within each reach, sandy sediments (250 μm to 3.15 mm size fraction) were collected from three independent patches (~10 m apart). All sampling devices were flame-sterilized in the field. Sediment samples were transferred into sterile cryovials, immediately flash-frozen in liquid nitrogen in the field and subsequently stored at −80°C before and following shipping to Switzerland for deoxyribonucleic acid (DNA) extraction and biomass analyses.

For each GFS, the distance to the glacier snout was calculated based on georeferencing (GPSMAPR 66 s, GARMIN) of the sampling reach, as well as glacier surface area and glacierized percentage catchment based on satellite imagery (Sentinel-2; Level 2a, March 2019–July 2022 from *scihub.copernicus.eu*) and a catchment definition derived from the ASTER Global Digital Elevation Model (GDEM) v3. (NASA/Meti/Aist/Japan Spacesystems and US/Japan Aster Science Team, 2019). The glacier index (GI) was calculated as $\frac{\sqrt{Glacier\ area}}{\sqrt{Glacier\ area}+ Distance\ to\ the\ glacier}$ according to Jacobsen & Dangles (2011) [35]. Benthic chlorophyll-*a*, a proxy for algal biomass, was extracted from the sediment (90% EtOH) in a hot (78°C) water bath for 10 min and further incubated (24 h, 4°C). After vortexing and centrifugation, chlorophyll-*a* concentration in the supernatant was quantified using a plate reader (BioTek Synergy H1; EX/EM: 436/680) and a spinach chlorophyll-a standard (Sigma Aldrich) and normalized to dry mass (DM) of sediment.

### Metagenomics

Metagenomes were sequenced for 149 sediment samples. DNA extraction, purification, library preparation, sequencing, and metagenome assembly steps were performed as described elsewhere [19]. Briefly, 5 g of sediments were treated using a phenol:chloroform-based extraction method subsequently followed by an ethanol precipitation step. This protocol yielded on average 50 ng of DNA per sample which was used for library preparation using the NEBNext Ultra II FS library kit, which also included 6 PCR cycles. Sequencing was performed at the Functional Genomics Centre Zurich using a S4 flowcell on a NovaSeq (Illumina).

The metagenomic sequence data was processed using the Integrated Meta-omic Pipeline (IMP) workflow (version 3.0; commit# 9672c874) [36]. Briefly, adapter trimming from reads using *trimmomatic* [37] is followed by an iterative assembly using *MEGAHIT* [38] and *Flye* [39]. To reduce computation time for binning, we removed sequences in the assembly <1.5 kbp and randomly selected 10% of the pre-processed reads using *seqtk* (v1.3). For each individual assembly, we then mapped the selected reads of the 5 spatially closest samples (Euclidean distances of gps coordinates) using *BWA-mem* (v0.7.17). We then used *MetaBAT2* (v2.15) [40], *CONCOCT* (v1.1.0) [41], and *MetaBinner* (v1.4.3) [42] using default parameters to obtain bins {see Code availability}. The quality of bins was assessed with *CheckM2* (v1.0.1) [43], and finally *DASTool* (v1.1.4) [44] was employed to generate a non-redundant set of bins using a score threshold of 0.3.

Bins from all samples (including the ones generated by *IMP3*) with a completeness of more than 50% were then selected for further analyses which accounted for 12 599 bins. We then used *MDMCleaner* (v0.8.3) [45] to reduce contamination of the bins. Finally, after rerunning *CheckM2* on the bins to get final estimates of completeness and contamination, we used *dRep* (v3.2.2) [46] to dereplicate bins using a minimum completeness of 70% and maximum contamination of 10% and an ANI of 99% to obtain 2855 strain-level MAGs. GTDB-Tk (v 2.1) [43, 47] was used to assign taxonomy to MAGs. We further used the concatenated alignment of 120 ubiquitous single-copy proteins created by GTDB-Tk to de novo generate a phylogenetic tree using *FastTree2* (v2.1.11) [48] under the WAG model of protein evolution with gamma-distributed rate heterogeneity. Functional annotation of the MAGs was performed with *eggNOG-Mapper* (v2.1.9) [49] after obtaining coding regions (CDS) with *prodigal* (v2.6.3) [50]. The coverage of MAGs was estimated by mapping reads of samples to the genomic contigs using *CoverM* (v0.6.1, available at https://github.com/wwood/CoverM) using the *trimmed_mean* parameter. We normalized the coverage by similarly mapping reads on the recA gene (K03553). For prevalence, presences were defined as abundance above a 10× recA coverage abundance threshold. However, one should keep in mind that metagenome-based analyses cannot differentiate between active, dormant or dead cells and that dispersal from upstream habitats may also influence patterns of prevalence in our dataset.

### Dimensions of glacial influence and community-weighted mean genomic properties

To identify the main environmental gradients across all GFS samples, Principal Component Analysis (PCA) was performed with the *prcomp* function in R (version 4.3.0), and using a non-redundant set of key physico-chemical as well as glacier-associated measures (glacier area, glacier coverage, GI, streamwater temperature, distance to the glacier, benthic chlorophyll-*a*). Community-weighted means (CWM) of genomic features [i.e. genome size, gene number, ribonucleic acid (tRNA) number, GC content, coding density, and gene redundancy index] were tested with linear effects against the first two principal components using generalized additive models (GAMs) created with the *bam* function of the *mgcv* R package (v1.9.0). For this, genomic features were first normalized using completeness and contamination as follows: ${value}_{normalised}= value\ast \left(1/ completeness\right)\ast \left(1- contamination\right).$CWM were then obtained by weighing normalized genomic features by MAG relative abundances and averaging across MAGs present in any given sample. To account for large-scale spatial patterns, we used a smoothed spline (bs = “sos”, k = −1) based on latitude and longitude in the GAMs. Detailed results of these GAMs are available in Supplementary Table 1. Significant linear effects (*P* < .01) were visualized using mean and standard errors of predictions across all GFS in the dataset. All figures were created using the *ggplot2* (version 3.4.3) and *ggpubr* (version 0.6.0) *R* packages (R version 4.3.0) [51, 52].

### Abundance-based phylogenetic permutation

To resolve the phylogenetic structure of CWM genomic features, we developed a null-model approach that randomly permutes abundances in a phylogenetic-bin based framework. For 40 values of relative phylogenetic height (h) uniformly distributed between zero and one (i.e. scanning the phylogenetic tree from the root to the tips), we performed phylogenetic agglomeration using the “average” method of the *hclust* R function on the cophenetic distances obtained with the *cophenetic.phylo* function of the *ape* R package (v5.7–1) [53]. Subsequently, for each value of h, abundances were randomly permuted within phylogenetic bins (20 iterations). Finally, GAMs accounting for spatial structure (i.e. including a smoothed spline (bs = “sos”, k = −1) on latitude and longitude as covariate) were created, testing for a linear effect of glacial influence on genomic features. Hence, this approach tests for associations between CWM genomic features and environmental parameter compared to null-model expectations across phylogenetic depth. This approach further allows identifying the relative depth at which phylogenetic signal in CWM genomic features appear along the gradients of glacier influence. Significant coefficients were assessed by combining *P*-values of the linear coefficients over the 20 iterations using Stouffer’s method in the *poolr* R package (v1.1–1), the mean and the standard deviation of the coefficients were computed to summarize the null-model permutations [54].

Additionally, this approach allowed us to pinpoint phylogenetic clades contributing to the community-level signal at a specific phylogenetic height. To this end, we used a leave-one-cluster-out approach, computing coefficients with and without a given phylogenetic cluster, and comparing the resulting coefficients’ distributions. Wilcoxon tests were used to test for difference in coefficient distributions, a median relative effect was computed comparing the median values with and without the target phylogenetic cluster [(value with – value without) / (value with)]. MAG taxonomy was used to summarize genera present within these clades. Additionally, to summarize these results at higher taxonomic level (i.e. to identify bacterial classes with disproportionately many MAGs in a phylogenetic cluster), we performed enrichment analyses using Fisher tests (fisher.test function in R). To account for multiple testing, we used the *p.adjust* R function using the Holm method.

### Functional potential

To unravel the functional potential associated with increased gene redundancy, we tested for each KO if the number of copies was higher in the MAGs that were part of significant clades compared to all other MAGs. We performed Wilcoxon tests (*wilcox.test* R function) on the log-transformed KO data (half of the minimal non-zero value was added to allow for zeroes in the dataset), and the *P*-values were adjusted using the *p.adjust* function in R with the “Bonferroni” method. KOs were considered significant if the *P-*value was <0, and the mean difference above zero. We then compared the KOs for all three relationships using intersects (*intersect* function in R).

We used LASSO regressions to identify functional genes that were associated with clades contributing signal to genomic properties (genome size, gene number, tRNA gene number) in relation to benthic chlorophyll-*a* concentration. For this, log-transformed KO data was used in a LASSO regression to explain the binomial response variable “part of clade” or “not part of clade”. The penalization in this regression type allowed to shrink the coefficient of non-important KOs to keep only KOs with high coefficients. We then compared the KOs for all three relationships using intersects (*intersect* function in R).

### Taxonomic summary

CWM genomic features of MAGs classified as *Gammaproteobacteria* were compared to all other MAGs. The taxonomic summary comparing genomic features of MAGs classified as *Gammaproteobacteria* to other taxonomic classes was created using the *dplyr* R package (v1.1.3). Wilcoxon sign rank tests were used to compare the distributions. Relative abundance and prevalence (i.e. the number of occurrences across GFSs) were used as estimates of the “ecological success” of MAGs. The assumption that abundant and prevalent MAGs in GFSs are ecologically successful is based on previous work, which show that GFS benthic communities assemble deterministically [23] and that benthic communities are distinct from the bacterial community suspended in the streamwater [17]. However, we acknowledge that we present results based on metagenomic dataset, and thus, dormant or inactive cells may be included. GAMs were built using a spline (k = 5, bs = “ts”) for these “ecological success” covariates, and genome size and coding density were used as response variables. We compared one model with a spline for all MAGs, and one with a different spline for GFS-*Gammaproteobacteria* and all other MAGs (using the “by” argument in the spline). A Bayes factor analysis was used to compare both models, using the *test_performance* function of the *performance* R package (v0.10.5). A Bayes factor above 3 was considered significant.

## Results and discussion

### Genome characteristics of the GFS microbiome

**Figure 1:**
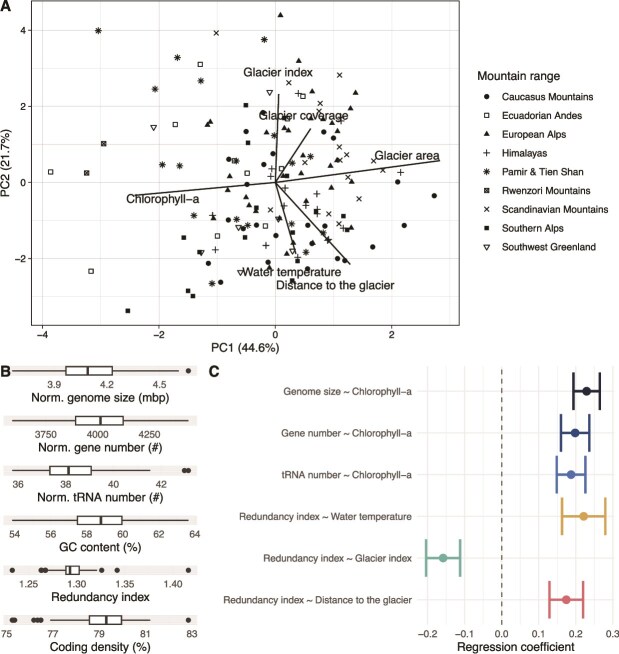
Dimensions of glacial influence and variation in genomic features. (A) the first two dimensions of a principal component analysis (PCA) depict associations among key glacier-associated environmental factors in GFS. Symbols represent mountain ranges; arrows depict scores of environmental variables. (B) Boxplot showing the distribution of community weighted mean genomic features (i.e. weighted with the relative abundance of MAGs) among GFSs. (C) Regression coefficients of genomic features that correlate with glacial covariates in the GAM analysis. GAMs considering spatial variations were fitted adding a linear effect for each pair of genomic features and glaciological variables. Significant relationships after adjusting *P*-values for multiple testing (Holm’s method, *P* < .05) are displayed.

The GFS environment is directly influenced by glaciers, primarily through the magnitude and variation of meltwater runoff [15, 27]. Runoff determines hydraulic stress, channel stability and sediment loads, while streamwater temperature affects metabolic processes [28, 55]. These physical processes are largely driven by glacier size, which translates into runoff magnitude and variability [55]. Employing PCA on the complete set of measured environmental parameters (complete dataset available as Supplementary Table 2), the first principal component (PC1; 44.6% explained variance) revealed a gradient of benthic chlorophyll-*a* inversely related to glacier area across all studied GFSs (Fig. 1A). This is striking given the overall low chlorophyll-*a* content (median: 0.0056 μg g^−1^ DM; IQR: 0.0007–0.0272) and underscores the responsiveness of benthic primary producers to environmental conditions. High runoff and loads of suspended sediments produced by large glaciers abrade benthic algae and attenuate light, thereby inhibiting primary production in GFSs and keeping chlorophyll-*a* concentrations low [56, 57]. Principal Component 2 (21.7% explained variance) depicts a gradient of streamwater temperature related to both distance to the glacier snout and glacier area (as encapsulated by the GI) across all GFSs. Indeed, depending on the magnitude of runoff, streamwater warms with increasing distance from the glacier. Taken together, the PCA reveals two main dimensions of glacial influence on GFSs at a global scale, and we will explore them as potential underpinning processes of the genomic landscape of the GFS microbiome.

Weighted by relative abundance of MAGs, bacterial genomes across all GFSs were relatively large in terms of size, had a high number of genes, and showed high GC content (Fig. 1B). These values are bracketed by those reported from other GFSs [29], various cryospheric ecosystems (e.g. permafrost, glacier ice) [6], and psychrophiles [5]. Bacterial genomes generally contain only little non-coding DNA (on average, ORFs account for 87% of genome size [58]). Hence, variation in gene number and genome size are generally tightly linked [2], a relationship attributed to the importance of effective population size [3]. GC content, coding density and genome size have also been shown to positively correlate in bacteria [5, 59, 60]. However, compared to psychrophilic, mesophilic, and thermophilic bacterial isolates [61], we found a relatively low number of tRNAs, which we mainly attribute to the discrepancy between MAGs and isolates owed to metagenomic assembly and binning [62, 63]. Because translation is energetically expensive, tRNA abundance has been linked to shorter minimal generation time and adaptability to different environmental conditions [64]. The gene redundancy index (i.e. the ratio between the total number of KOs to the number of unique KOs with a genome, median RI ~ 1.4) was lower than previously reported in cryoconite biofilms [65], which we attribute to the dynamic and unpredictable GFS environment that may select for functional plasticity rather than redundancy within a given genome.

To further explore glacier influence on these genomic properties of the GFS microbiome, we implemented GAMs accounting for large-scale spatial variation and isolating linear effects of environmental parameters on genomic properties. GAMs revealed positive associations between benthic chlorophyll-*a* content (correlated with PC1) with average genome size, gene number, and tRNA number, whereas covariates correlating with PC2 (i.e. water temperature, distance from the glacier and GI) were associated with the gene redundancy index (Fig. 1C). These findings are in line with previous work suggesting that benthic algae, through the exudation of energy-rich macromolecules, relieve GFS bacteria from energy and carbon limitation [28], ultimately promoting bacteria with larger genomes as glaciers shrink and benthic algal biomass increases [26]. Indeed, metabolic interactions between microbial heterotrophs and algae have been repeatedly reported from stream biofilms [66, 67], which may be particularly important in GFSs largely devoid of allochthonous sources of organic carbon [19, 28]. Furthermore, these analyses revealed increasing numbers of tRNAs with diminishing glacial influence, which essentially follows the observed trends in genome size (Fig. 1C). While tRNAs have been associated with cold adaptation and post-translational modifications in bacteria [68, 69], work on isolates showed that psychrophile genomes have elevated numbers of tRNAs [61]. Nevertheless, translational efficiency has been shown to be low in organisms that are able to thrive in multiple habitats, and this could potentially explain the low number of tRNAs that we observed [64]. Importantly, our analyses have not revealed any major variation in coding density along any of the glaciological variables tested. In line with expectations [3], this suggests that genome size, number of genes and thus the proportion of non-coding DNA vary concomitantly across environmental gradients in GFS.

### Dissecting the phylogenetic signatures of genomic trends along environmental gradients

Variation in community-level genomic properties along environmental gradients can either arise from changes in abundance or the replacement of taxa with different genomic characteristics. Moreover, shared evolutionary histories of microbiome members can shape relationships between genomic properties and environmental constraints [34]. For example, accounting for phylogenetic dependencies, a previous study identified deep phylogenetic signatures in genome size variation of bacteria and archaea [34]. To assess phylogenetic signatures in genomic features, we developed a null model-based approach to first identify the phylogenetic depth at which signal in genomic properties along environmental gradients arise. Using a leave-one-out approach of individual clades at the identified threshold phylogenetic distance, we then find clades that contribute most to this signal. Finally, we investigate the functional potential of these clades in comparison to other community members, to uncover functional traits associated with community-level genomic properties.

**Figure 2:**
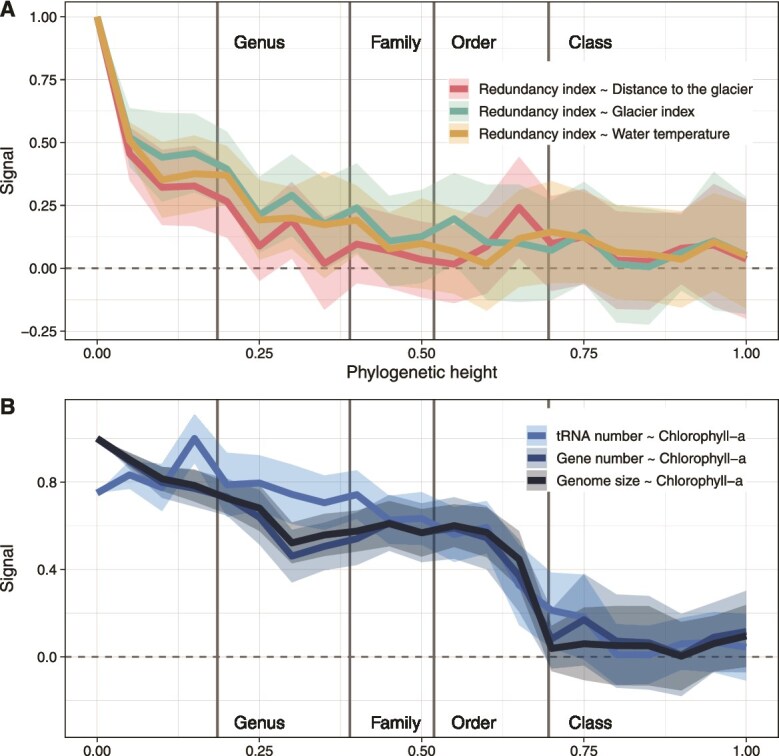
The signal between CWM of genomic features and glacier influence is phylogenetically structured. Line plots displaying the signal in relationships between the gene redundancy index as response variable and the distance to the glacier, the GI and the water temperature as covariates (A) and between gene number, genome size and tRNA number and chlorophyll-*a* as covariate (B). The signal was assessed using linear coefficients in the GAMs taking spatial variation into account when permuting abundances at various relative phylogenetic heights. Coefficients were normalized by the maximal value for any given glacial covariate-genomic feature pair over the various phylogenetic height values. Shaded areas represent the standard error obtained through 20 null model iterations. Vertical lines indicate median phylogenetic heights for different taxonomic levels and are for visual guidance only.

We found significant phylogenetic signature exclusively at low depth (i.e. among closely related members) for relationships between the gene redundancy index and streamwater temperature, GI and distance to the glacier (<0.25 relative phylogenetic tree height, corresponding approximately to median genus-level phylogenetic depth; Fig. 2A). This suggests that variation in the gene redundancy index is predominantly structured among closely related taxa.

Using the leave-one-cluster-out approach, we identified 36 clusters (out of a total of 394 clusters at a phylogenetic depth of 0.25, Supplementary Table 3) containing MAGs classified as *ELB16–189* (*n* = 42), *OLB17* (*n* = 16), *CAILRJO1* (*n* = 10), *Palsa-1315* (*n* = 10), *Deinococcus* (*n* = 5) and *Nitrospira_F* (*n* = 1) to drive relationships between the distance to the glacier and the gene redundancy index. This highlights the fine-scaled yet widely distributed phenomenon that GFS taxa possess increased gene redundancy at decreased glacial influence. Interestingly, more clusters were significant for the distance to the glacier (*n* = 36) compared to the GI (*n* = 5) and streamwater temperature (*n* = 1). Both, the GI and distance to the snout may integrate the longer-term influence of glaciers on the GFS microbiome whereas streamwater temperature fluctuates on timescales of minutes to hours [70].

To unravel which microbial functions exhibit increased redundancy in GFS microbiomes under reduced glacial influence, we compared the number of gene copies per KO in clades with and without significant relationships between redundancy and glacial influence, respectively. We identify a total of 37 KOs with significantly higher copy numbers (Table 1, Wilcoxon rank-sum test, adjusted *P*-value <.01). These include several genes associated with metabolism, including two genes related to sulfur metabolism (*ddhA*, *ddhB*), two genes encoding methane/ammonia monooxygenase subunits (B and C), and carbon-metabolism related genes (*acsE*, *ccsB*, *sucD*, *korD*). This observation aligns with previous findings [26, 28], who reported that declining environmental selection in GFSs promotes primary production, leading to shifts in microbiome functions, including changes in energy acquisition pathways. Taken together, increased gene redundancy in metabolic pathways with reduced glacial influence, may point toward an adaptive strategy of microbes to cope with environmental changes in GFSs.

**Table 1:** KEGG orthologs (KOs) that were significantly (*P* < .01) more redundant in the MAGs associated with increase gene redundancy index against all three tested glaciological parameters (distance to the glacier, GI, and water temperature). These were tested using Wilcoxon tests, and *P-*values were corrected using the Bonferroni method, only KOs with positive mean differences (i.e. higher redundancy) are displayed. The descriptions and pathways were obtained from the KEGG website (https://www.kegg.jp/entry/).

KO	Symbol and description	Pathways
K00003	hom, homoserine dehydrogenase	Glycine, serine and threonine metabolism / Cysteine and methionine metabolism / Lysine biosynthesis / Metabolic pathways / Biosynthesis of secondary metabolites / Microbial metabolism in diverse environments / Biosynthesis of amino acids
K00113	glpC, glycerol-3-phosphate dehydrogenase subunit C	Glycerophospholipid metabolism / Biosynthesis of secondary metabolites
K00176	korD, oorD, 2-oxoglutarate ferredoxin oxidoreductase subunit delta	Citrate cycle (TCA cycle) / Other carbon fixation pathways / Metabolic pathways / Biosynthesis of secondary metabolites / Microbial metabolism in diverse environments / Carbon metabolism / 2-Oxocarboxylic acid metabolism
K00311	ETFDH, electron-transferring-flavoprotein dehydrogenase	
K00373	narJ, narW, nitrate reductase molybdenum cofactor assembly chaperone NarJ/NarW	Two-component system
K00543	ASMT, acetylserotonin O-methyltransferase	Tryptophan metabolism / Metabolic pathways
K00688	PYG, glgP, glycogen phosphorylase	Starch and sucrose metabolism / Metabolic pathways / Biosynthesis of secondary metabolites / Biofilm formation - *Escherichia coli*
K00979	kdsB, 3-deoxy-manno-octulosonate cytidylyltransferase (CMP-KDO synthetase)	Biosynthesis of various nucleotide sugars / Metabolic pathways / Biosynthesis of nucleotide sugars
K01206	FUCA, alpha-L-fucosidase	Other glycan degradation / Lysosome
K01665	pabB, para-aminobenzoate synthetase component I	Folate biosynthesis / Biosynthesis of cofactors
K01839	deoB, phosphopentomutase	Pentose phosphate pathway / Purine metabolism / Metabolic pathways
K01902	sucD, succinyl-CoA synthetase alpha subunit	Citrate cycle (TCA cycle) / Propanoate metabolism / C5-Branched dibasic acid metabolism / Other carbon fixation pathways / Metabolic pathways / Biosynthesis of secondary metabolites / Microbial metabolism in diverse environments / Carbon metabolism
K02237	comEA, competence protein ComEA	
K03167	top6B, DNA topoisomerase VI subunit B	
K03581	recD, exodeoxyribonuclease V alpha subunit	Homologous recombination
K04477	ycdX, putative hydrolase	
K04767	acuB, acetoin utilization protein AcuB	
K05524	fdxA, ferredoxin	
K05809	raiA, ribosome-associated inhibitor A	
K06998	phzF, trans-2,3-dihydro-3-hydroxyanthranilate isomerase	
K07019	uncharacterized protein	
K07126	uncharacterized protein	
K10700	edbA, ethylbenzene hydroxylase subunit alpha	Ethylbenzene degradation / Metabolic pathways / Microbial metabolism in diverse environments / Degradation of aromatic compounds
K10945	pmoB-amoB, methane/ammonia monooxygenase subunit B	Methane metabolism / Nitrogen metabolism / Metabolic pathways / Microbial metabolism in diverse environments / Carbon metabolism / Nitrogen cycle
K10946	pmoC-amoC, methane/ammonia monooxygenase subunit C	Methane metabolism / Nitrogen metabolism / Metabolic pathways / Microbial metabolism in diverse environments / Carbon metabolism / Nitrogen cycle
K13795	citB, tcuB, citrate/tricarballylate utilization protein	
K15023	acsE, 5-methyltetrahydrofolate corrinoid/iron sulfur protein methyltransferase	Other carbon fixation pathways / Metabolic pathways / Microbial metabolism in diverse environments / Carbon metabolism
K15233	ccsB, citryl-CoA synthetase small subunit	Other carbon fixation pathways / Metabolic pathways / Microbial metabolism in diverse environments / Carbon metabolism
K16130	mcyA, microcystin synthetase protein McyA	Nonribosomal peptide structures
K16964	ddhA, dimethylsulfide dehydrogenase subunit alpha	Sulfur metabolism / Metabolic pathways / Microbial metabolism in diverse environments
K16965	ddhB, dimethylsulfide dehydrogenase subunit beta	Sulfur metabolism / Metabolic pathways / Microbial metabolism in diverse environments
K17048	edbB, ethylbenzene hydroxylase subunit beta	Ethylbenzene degradation / Metabolic pathways / Microbial metabolism in diverse environments / Degradation of aromatic compounds
K17052	serC, clrC, selenate/chlorate reductase subunit gamma	Selenocompound metabolism
K18896	gsmt, glycine/sarcosine N-methyltransferase	Glycine, serine and threonine metabolism / Metabolic pathways
K18897	sdmt, sarcosine/dimethylglycine N-methyltransferase	Glycine, serine and threonine metabolism / Metabolic pathways
K20435	valM, validone 7-phosphate aminotransferase	Acarbose and validamycin biosynthesis / Metabolic pathways / Biosynthesis of secondary metabolites
K21515	aviRa, 23S rRNA (guanine2535-N1)-methyltransferase	

### Members of Gammaproteobacteria shape the relationship between genomic features and chlorophyll-a

In contrast to gene redundancy, relationships of genome size, gene number, and tRNA number with benthic chlorophyll-*a* concentration arose already at greater phylogenetic depths (~0.6 relative phylogenetic tree height, corresponding to median class-level depth, Fig. 2B). This signal was conserved across the lower range of the phylogenetic tree. Leave-one-cluster-out analysis highlighted the contribution of a single cluster to signal for all three genome properties - comprising all MAGs classified as *Gammaproteobacteria* in our dataset (termed GFS-Gammaproteobacteria, Supplementary Table 4). Additionally, significant changes in genome size and gene number along the benthic chlorophyll-*a* gradient were found for phylogenetic clusters encompassing MAGs classified as *Acidobacteriota*, *Desulfobacterota*, *Myxococcota*, and *Nitrospirota*. This finding aligns with previous work on GFS community assembly, which found that homogeneous selection promotes microdiversity among *Gammaproteobacteria* (from the *Burkholderiales* order previously assigned to *Betaproteobacteria*) and *Nitrospira* among a few other taxa [23]. Moreover, these results highlight the importance of chlorophyll-*a* in profoundly shaping the structure of the GFS microbiome. We deem the fact that chlorophyll-*a* concentration, a biological factor, is more important in shaping deep-rooting genomic signatures than physical factors (e.g. temperature) particularly relevant considering the importance of algal-bacterial interactions [19] and pronounced carbon limitation in GFS [28]. This may point to the long-term coherence of these drivers– which are now changing in GFSs due to climate-change induced retreat of glaciers.

**Figure 3:**
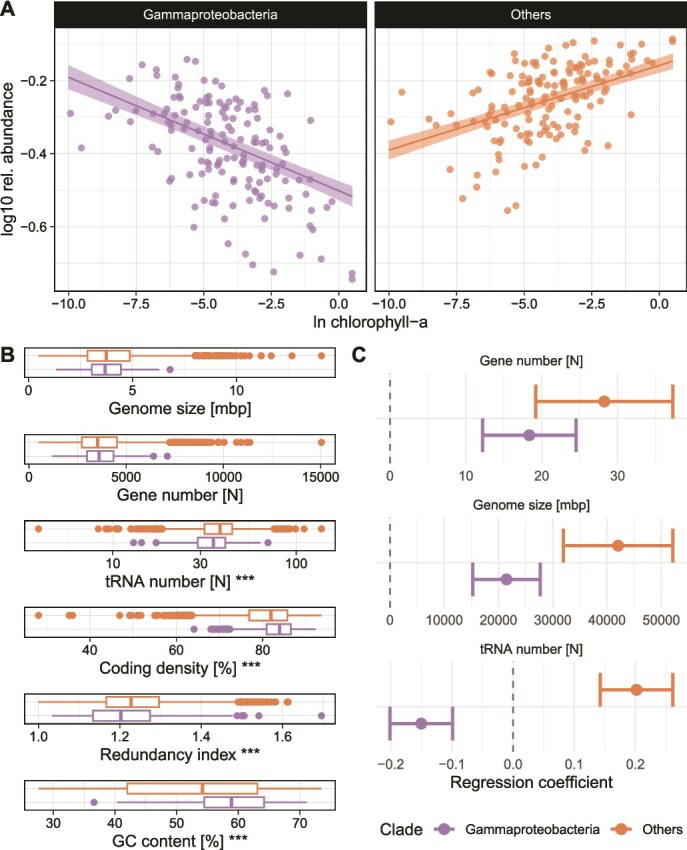
GFS-Gammaproteobacteria drive the variation in genomic features along the gradient of chlorophyll-*a* (A) scatterplot showing the variation in the relative abundance of GFS-Gammaproteobacteria and all other MAGs along the gradient of benthic chlorophyll-*a* in the world’s GFSs. Lines show linear GAM fits accounting for large-scale spatial patterns; shaded areas show prediction intervals. (B) Distributions of genomic features for GFS-Gammaproteobacteria and other MAGs are displayed. Stars denote significance (*P* < .01) of Wilcoxon signed rank tests comparing the two groups. (C) Linear GAM coefficients representing the variation of genomic feature averages.

Given the abundance and prevalence of GFS-Gammaproteobacteria [18, 21, 23], we next investigated the genomic properties of GFS-Gammaproteobacteria in relation to glacier influence (Fig. 3). Indeed, we found a strong negative relationship between relative abundance of GFS-Gammaproteobacteria and benthic chlorophyll-*a* (Fig. 3A). GFS-Gammaproteobacteria had significantly increased coding density (median difference: 2%), but fewer tRNAs (median difference: 3.05), and a lower gene redundancy index (median difference: 0.025) compared to all other MAGs in our dataset (Fig. 3B). On the other hand, genome size and gene numbers of GFS-Gammaproteobacteria were not significantly different from other MAGs. This contrasts our findings on community-weighted average genomic features and suggests that abundance differences of GFS-Gammaproteobacteria across gradients of glacier influence may contribute to the microbiome-weighted averages.

**Figure 4:**
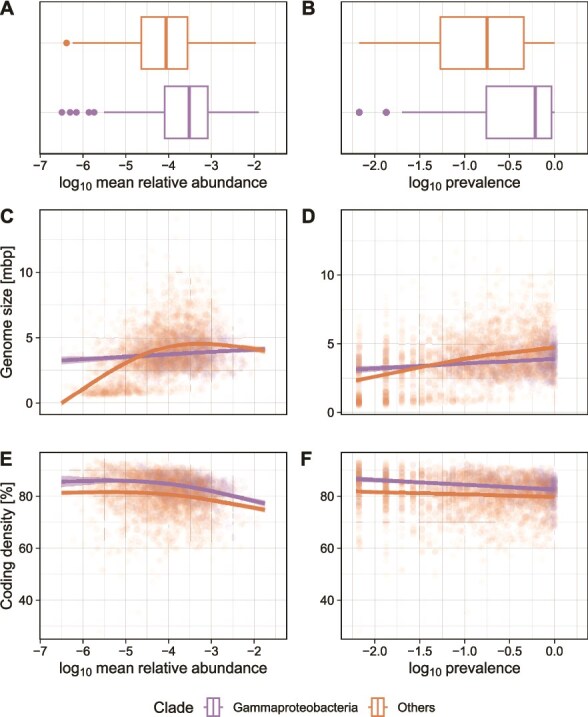
GFS-Gammaproteobacteria are abundant and prevalent. Comparison of relative abundance (A) and prevalence (B) in GFS of MAGs affiliated to *Gammaproteobacteria* and other classes. Smoothed splines representing GAMs comparing mean abundance (C and E) and prevalence (D and F) with normalized genome size (C and D) and coding density (E & F). Models with separate splines for GFS-Gammaproteobacteria and all other MAGs were better supported (Bayes factor > 1000) than a combined model. While the difference is driven by high abundance and prevalence of large genomes among other classes (or conversely the absence of small genomes at low abundance and prevalence in GFS-Gammaproteobacteria), GFS-Gammaproteobacteria exhibit increased values for coding density across the entire gradient.

Next, we examined relationships between genomic properties and prevalence and mean relative abundance of MAGs across our global repository of GFSs. Looking at the distribution of mean relative abundance and prevalence, we find that the GFS-Gammaproteobacteria harbor representatives with high values (Fig. 4A and D, Wilcoxon tests, adjusted *P*-values <.001, log median difference *=* 0.54 for both, relative abundance and prevalence). Additionally, we found positive relationships between genome size and MAG prevalence and relative abundance (Fig. 4C and D, whereas coding density was negatively related to prevalence and abundance (Fig. 4E and F). Using GAMs and a Bayes factor analysis, we tested whether these relationships differed between GFS-Gammaproteobacteria and other MAGs. A GAM with separate splines for GFS-Gammaproteobacteria and other MAGs was better supported by the data (Bayes factor > 1000 for all comparisons) than a GAM with one spline for all MAGs (Fig. 4). This indicates that GFS-Gammaproteobacteria combine increased coding density with reduced genome size compared to classes that are similarly abundant and prevalent in GFSs. Given the compositional nature of microbiomes, this relationship could in part (at low prevalence) also be driven by the low prevalence of symbiotic *Patescibacteria* that have particularly small and streamlined genomes and seem to show low dispersal capabilities [71].

However, that the signal in genomic properties along the chlorophyll-*a* gradient was conserved across phylogenetic depths indicates that not the entire GFS-Gammaproteobacteria clade but rather specific sub-clades may drive this relationship. Indeed, the leave-one-cluster-out analysis performed at shallower phylogenetic depth (relative phylogenetic depth = 0.2) identified specific clades including *Polaromonas* (n_MAGs_ = 25), *Rhodoferax* (n_MAGs_ = 21), JAAFIP01 (n_MAGs_ = 23), *Aquabacterium_A* (n_MAGs_ = 20), and *Rubrivivax* (n_MAGs_ = 27). Other notable taxa included *Novosphingobium* (n_MAGs_ = 42) and the *Patescibacteria* genus *OLB19* (n_MAGs_ = 32). The signal among multiple genus-level clades across the GFS-Gammaproteobacteria suggests that the observed increase in genome size with higher chlorophyll-*a* concentrations (or, inversely, the reduction in genome size under high glacial influence when benthic chlorophyll-*a* concentration is particularly low) may result from either convergent evolution across diverse lineages or an early adaptive expansion within the GFS-*Gammaproteobacteria*. However, further phylogenomic analyses would be needed to be better understand the mechanisms and timescales of these processes. As a first step, we provide comparative pangenome analyses between GFS-Gammaproteobacteria and their sister clade in SI (Supplementary information, section “Pangenome analyses”).

**Table 2:** KEGG orthologs (KOs) that were identified by the LASSO regression analysis to be enriched in clades driving variations for both genome size and gene numbers, along the gradient of chlorophyll-*a*. The descriptions and pathways were obtained on the KEGG website (https://www.kegg.jp/entry/), human diseases related pathways were not included.

**KO**	**Symbol & Description**	**Pathways**
K00028	malate dehydrogenase (decarboxylating)	Pyruvate metabolism / Carbon fixation by Calvin cycle / Metabolic pathways / Microbial metabolism in diverse environments / Carbon metabolism
K00090	ghrB, glyoxylate/hydroxypyruvate/2-ketogluconate reductase	Pentose phosphate pathway / Glycine, serine and threonine metabolism / Pyruvate metabolism / Glyoxylate and dicarboxylate metabolism / Metabolic pathways / Biosynthesis of secondary metabolites / Microbial metabolism in diverse environments
K00525	E1.17.4.1A, nrdA, nrdE, ribonucleoside-diphosphate reductase alpha chain	Purine metabolism / Pyrimidine metabolism / Metabolic pathways / Nucleotide metabolism
K00571	E2.1.1.72, site-specific DNA-methyltransferase (adenine-specific)	
K00646	pksF, curC, aprD, corD, malonyl-[acp] decarboxylase	
K01141	sbcB, exoI, exodeoxyribonuclease I	Mismatch repair
K01432	AFMID, arylformamidase	Tryptophan metabolism / Glyoxylate and dicarboxylate metabolism / Metabolic pathways / Biosynthesis of cofactors
K01491	folD, methylenetetrahydrofolate dehydrogenase (NADP+) / methenyltetrahydrofolate cyclohydrolase	One carbon pool by folate / Other carbon fixation pathways / Metabolic pathways / Microbial metabolism in diverse environments / Carbon metabolism / Biosynthesis of cofactors
K01626	E2.5.1.54, aroF, aroG, aroH, 3-deoxy-7-phosphoheptulonate synthase	Phenylalanine, tyrosine and tryptophan biosynthesis / Metabolic pathways / Biosynthesis of secondary metabolites / Biosynthesis of amino acids / Quorum sensing
K01673	cynT, can, carbonic anhydrase	Nitrogen metabolism / Metabolic pathways
K01952	PFAS, purL, phosphoribosylformylglycinamidine synthase	Purine metabolism / Metabolic pathways / Biosynthesis of secondary metabolites
K02083	allC, allantoate deiminase	Purine metabolism / Metabolic pathways / Microbial metabolism in diverse environments
K02568	napB, nitrate reductase (cytochrome), electron transfer subunit	Nitrogen metabolism / Metabolic pathways / Microbial metabolism in diverse environments / Nitrogen cycle
K03169	topB, DNA topoisomerase III	
K03198	virB3, lvhB3, type IV secretion system protein VirB3	Bacterial secretion system
K03442	mscS, small conductance mechanosensitive channel	
K03775	slyD, FKBP-type peptidyl-prolyl cis-trans isomerase SlyD	
K03818	wcaF, putative colanic acid biosynthesis acetyltransferase WcaF	Exopolysaccharide biosynthesis
K03832	tonB, periplasmic protein TonB	
K05962	protein-histidine pros-kinase	
K06192	pqiB, paraquat-inducible protein B	
K07025	putative hydrolase of the HAD superfamily	
K07114	yfbK, Ca-activated chloride channel homolog	
K07343	tfoX, DNA transformation protein and related proteins	
K07684	narL, two-component system, NarL family, nitrate/nitrite response regulator NarL	Two-component system
K07712	glnG, ntrC, two-component system, NtrC family, nitrogen regulation response regulator GlnG	Two-component system
K10012	arnC, pmrF, undecaprenyl-phosphate 4-deoxy-4-formamido-L-arabinose transferase	Biosynthesis of various nucleotide sugars / Metabolic pathways / Cationic antimicrobial peptide (CAMP) resistance
K10537	araF, L-arabinose transport system substrate-binding protein	ABC transporters
K12055	parA, chromosome partitioning related protein ParA	
K12500	tesC, thioesterase III	
K12601	SKI8, superkiller protein 8	RNA degradation
K12602	WDR61, REC14, SKI8, WD repeat-containing protein 61	RNA degradation
K13117	DHX35, ATP-dependent RNA helicase DDX35	
K14160	imuA, protein ImuA	
K14742	tsaB, tRNA threonylcarbamoyladenosine biosynthesis protein TsaB	
K15653	mxcG, nonribosomal peptide synthetase MxcG	Biosynthesis of siderophore group nonribosomal peptides
K17218	sqr, sulfide:quinone oxidoreductase	Sulfur metabolism / Microbial metabolism in diverse environments
K20036	dmdD, (methylthio)acryloyl-CoA hydratase	Sulfur metabolism / Metabolic pathways / Microbial metabolism in diverse environments
K20534	gtrB, polyisoprenyl-phosphate glycosyltransferase	
K20906	hcmA, 2-hydroxyisobutanoyl-CoA mutase large subunit	
K21211	ncsC1, NDP-hexose 4,6-dehydratase	
K21394	Biosynthesis of enediyne antibiotics / Biosynthesis of secondary metabolites	
K21405	acoR, sigma-54 dependent transcriptional regulator, acetoin dehydrogenase operon transcriptional activator AcoR	
K21739	rclA, probable pyridine nucleotide-disulfide oxidoreductase	
K21843	TTC7, tetratricopeptide repeat protein 7	

We next sought to identify the functional potential associate with the differences in genome size, gene number and tRNA number along gradients of chlorophyll-*a*. We applied lasso regression to pinpoint KOs associated with clades exhibiting significant signal. This approach enabled us to identify functions that were enriched in these clusters, representing candidate drivers of expanded functional potential. While we observed 47 KOs that were shared for genome size and gene number (Table 2), not a single significant clade (and hence KO) was found for the relationship between tRNA number and chlorophyll-*a* at shallower depth. This may be attributable to a generally weaker signal observed for tRNA compared to genome size and gene number (as reflected in the *P*-values of the GAMs), and potentially also to the tendency for tRNA genes to be underrepresented on MAGs due to metagenomic binning.

Nevertheless, for genome size and gene number, several metabolic pathways—including pyruvate metabolism, the glyoxylate cycle, and nucleotide biosynthesis—were represented by key enzymes such as malate dehydrogenase (decarboxylating), glyoxylate/hydroxypyruvate/2-ketogluconate reductase, and ribonucleotide reductases. Nitrogen and sulfur metabolism were also represented, with genes like *napB*, *cynT*, and *sqr* suggesting chemolithoautotrophy, typical of oligotrophic glacier-related systems [21, 72, 73]. Notably, genes involved in quorum sensing and secondary metabolite biosynthesis—such as the *aroF/G/H* cluster and *mxcG* (non-ribosomal peptide synthetase)—point to increased microbial interactions and competition at reduced glacial influence, which is compatible with the “greening” of GFSs [26, 28]. Two-component systems (*narL*, *glnG*) and secretion-related proteins (*virB3*) further highlight regulatory complexity linked to environmental responsiveness, potentially a crucial adaptation to the fluctuating environmental conditions of GFSs. Overall, and given the taxonomic diversity and variety of functional adaptations observed, more targeted, taxon-specific analyses will be necessary to gain deeper insights into the ecological strategies of individual lineages.

## Conclusions

Evolutionary history and environmental constraints shape the genomic architecture of microbial communities, ultimately with consequences for diversity and function. Here, we developed a phylogeny-rooted analytical framework that unravels signatures of genomic trends in the world’s GFSs. The approach allows pinpointing the phylogenetic depth at which these signatures arise and the importance of individual clades at shaping community-level genomic features. We find significant variation in genome size, gene number, tRNA gene numbers, and modulation of genomic redundancy along gradients of glacial influence. Collectively, our findings suggest that the selective constraints in GFSs explain microbiome-level patterns in genome architecture and that changes in genomic features mainly occur via changes in abundance among specific GFS-Gammaproteobacteria clades. We deem these findings critical because the deep phylogenetic rooting of these signatures reflects the long-term and putatively consistent nature of this extreme environment, which is now changing at a rapid pace owing to climate change.

## Consortium

The Vanishing Glaciers Field Team

Michael Styllas^1^, Matteo Tolosano^1^, Martina Schön^1^, Vincent de Staercke^1^, and Tyler Kohler^2^.

1. River Ecosystems Laboratory, Alpine and Polar Environmental Research Center, École Polytechnique Fédérale de Lausanne (EPFL), Sion, Switzerland
2. Department of Ecology, Faculty of Science, Charles University, Prague, Czechia

## Supplementary Material

MAGFS_final_pangenome_SI_ycaf076

MAGFS_final_SI_tables_ycaf076

## Data Availability

The code and data used in this study are available on the GitHub repository: https://github.com/Mass23/MAGFS. The MAGs are deposited on NCBI under the BioProject PRJNA781406. Additionally, the code for binning is available on this link: https://github.com/michoug/VanishingGlacierMAGs.
