# Supplementary material for: Glacier influence shapes the genomic architecture of the downstream aquatic microbiome
Source: ISME Commun. 2025 May 14;5(1):ycaf076. doi: 10.1093/ismeco/ycaf076 (PMC12684718; doi:10.1093/ismeco/ycaf076)
Supplement: MAGFS_final_pangenome_SI_ycaf076 [file magfs_final_pangenome_si_ycaf076.pdf]

Supplementary Information

Glacier influence shapes the genomic architecture of the downstream aquatic microbiome

Massimo Bourquin[1,*], Hannes Peter[1], Grégoire Michoud[1], Aileen Geers[1], Susheel Bhanu Busi[2], The Vanishing Glaciers Field Team**, Tom Ian Battin[1,*]

(1) River Ecosystems Laboratory, Alpine and Polar Environmental Research Center, Ecole Polytechnique Fédérale de Lausanne, EPFL, Lausanne, Switzerland
(2) UK Centre for Ecology and Hydrology, Wallingford, United Kingdom

*corresponding authors: *Massimo.bourquin@epfl.ch*, *tom.battin@epfl.ch*

****The Vanishing Glaciers Field Team**: Mike Styllas, Matteo Tolosano, Martina Schön, Vincent de Staercke, Tyler Kohler

Pangenome analysis to identify potential adaptations of GFS-Gammaproteobacteria

Since we observe distinct genomic patterns within the GFS-Gammaproteobacteria, their origin could stem from multiple eco-evolutionary processes. These include early adaptations that occurred in the most recent common ancestor (MRCA) of Gammaproteobacteria in glacier-fed streams (GFS), or more recent diversification events potentially driven by horizontal gene transfer (HGT). However, given the pronounced phylogenetic signal observed, we hypothesized that early evolutionary processes played a central role. To investigate this, we performed a pangenome analysis of the monophyletic GFS-Gammaproteobacteria clade to identify a conserved set of core genes. We then compared this core pangenome to that of both Gamma- and Alphaproteobacteria—using the latter as a paraphyletic outgroup within the GFS microbiome—to trace patterns of early gene retention or loss. This comparative approach could potentially pinpoint genes consistently gained or lost in the GFS-Gammaproteobacteria relative to their ancestral state, offering insight into potential early, adaptive evolutionary events specific to their diversification in the GFS ecosystem. However, we acknowledge that further tree reconciliation based analyses will be required to validate these findings.

Nevertheless, our analysis allowed to uncover a core pangenome of 830 gene clusters for GFS-Gammaproteobacteria, and 548 gene clusters when Alphaproteobacteria MAGs were also included. Analysing KEGG orthologous groups functions (KOs) of putative gene gains and losses, we identified 309 KOs gained and 273 KOs lost in the GFS-Gammaproteobacteria. Both putative gene gains and losses were predominantly concentrated in transporters, enzymes, and the two-component system (Supplementary figure 1). Additionally, putative gene gains were observed in secretion systems, lipopolysaccharide biosynthesis, and bacterial motility, while potential gene losses were most common in butanoate metabolism, glyoxylate and

dicarboxylate metabolism, amino sugar and nucleotide sugar metabolism, and fructose and mannose metabolism. In the context of GFSs, these putative losses observed in several metabolism pathways could be associated with the scarcity of organic carbon.

By applying a text-mining approach, we identified higher-level functions associated with these gains and losses. Notably, we observed the gain of 20 ATP-binding transporters related to twitching motility and type IV pili, while one KO associated with flagella was lost. While bacteria in biofilms are typically less motile than their free-living counterparts [1], twitching motility through type IV pili is well known for its implications in biofilm formation and movement of prokaryotes on surfaces [2]. Moreover, five KOs related to chemotaxis were putative gene gains while one was lost. In case this result arises from evolutionary signal, this could potentially suggest a change in motility of GFS-Gammaproteobacteria. We also noted the loss of nine KOs linked to fermentation, particularly in the butanoate metabolism category, and the gain of seven KOs associated with lipids. While the losses in fermentation and especially butanoate metabolism could be attributed to the high oxygenation of the GFS environment, lipids (e.g., lipopolysaccharides) have been associated with the adaptation of membranes and cell walls to cold conditions [3]. While we find putative KO gains associated with cold adaptation and a biofilm lifestyle, we observe losses that could be adaptive given the association we observed between genome size and glacier influence. Interestingly, several functional categories identified through the lasso regression approach—such as pyruvate metabolism, the glyoxylate cycle, sulfur and nitrogen metabolism, quorum sensing, and two-component regulatory systems—are shared with the pangenome-based analysis of GFS-Gammaproteobacteria. In both cases, functions related to environmental responsiveness (e.g., chemotaxis, secretion systems) were prominent. This overlap strengthen the hypothesis that these functional traits represent adaptation to glacier-fed streams with reduced glacier influence.

Supplementary Figure 1

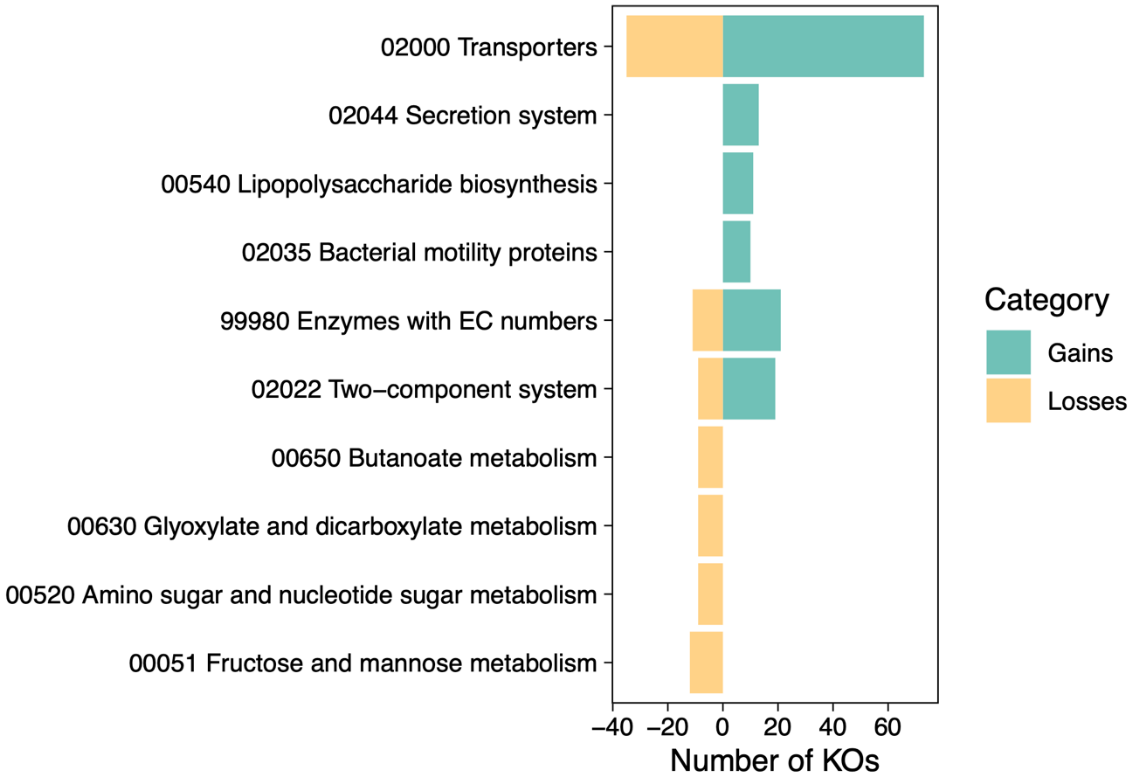

Supplementary Figure 1. KO gains and losses of GFS-Gammaproteobacteria compared to the closest sister clade (*Alphaproteobacteria*). Shown are functional categories that contain the highest number of gene gains and losses in GFS-Gammaproteobacteria. Gains were defined as KOs present in the core genome of GFS-Gammaproteobacteria, but not in the core genome when GFS-Alphaproteobacteria were included. Losses were defined as KOs that were present in the combined core pangenome, but not in the core genome of GFS-Gammaproteobacteria.

Methods

We used *mOTUpan* [4] to create pangenomes of high-quality MAGs defined as a completeness higher than 90% (median: 95.06%; IQR: 92.22 - 98.05%), and a contamination threshold of <10% (median: 1.91%; IQR: 0.99 - 3.42%). The first pangenome was created with all MAGs from the phylogenetic bin (n=127, identified with the phylogenetic permutation) including all representatives of the GFS-Gammaproteobacteria. A second pangenome (n=223) was created with GFS-Gammaproteobacteria and MAGs classified as Alphaproteobacteria. Default parameters were used on the .faa files, and the *CheckM2* output was used to initiate completeness values, the --seed parameter was set to 90. KEGG ortholog (KO) and CAZyme annotations were used to assign functions to each gene cluster. We then summarised the KOs and CAZymes present in the core genome of both pangenomes in a comparative genomics approach, with the idea to identify functions putatively gained and lost early in the evolution of *Gammaproteobacteria* within the GFS ecosystem compared to the *Alphaproteobacteria* paraphyletic clade. Such an approach was deemed more powerful than including genomes from other environments as it is not biased by horizontal gene transfer in other ecosystems, and thus, reflects selection pressure on the microbiome in GFS. KOs putatively gained by GFS-Gammaproteobacteria were defined as those that were part of the core genome of the Gammaproteobacteria pangenome, but not in the pangenome created including Alphaproteobacteria MAGs. Similarly, gene losses were defined as genes that were part of the core genome in the pangenomes of both classes, but not in the pangenome of GFS-Gammaproteobacteria. KEGG pathways and categories were then used to summarise potential gene losses and gains using a “text-mining” approach. The approach consisted in using the description of KOs (available at: https://www.genome.jp/kegg/) to identify words that occurred often in the set of KOs putatively gained and lost. The number of descriptions matching queries of interest was computed using regular expression (*regex*) subsetting with the *grepl* function of

R [5]. The queries of interest were based on words that were found in the greatest number of genes descriptions, they included the words: “ATP-binding”, “motility”, “chemotaxis”, “CoA-transferase”, and the suffixes “flagell-”, and “lipo-”.

References

1. Guttenplan SB, Kearns DB. Regulation of flagellar motility during biofilm formation. *FEMS Microbiol Rev* 2013;**37**:849–871. https://doi.org/10.1111/1574-6976.12018

2. Burrows LL. Pseudomonas aeruginosa Twitching Motility: Type IV Pili in Action. *Annu Rev Microbiol* 2012;**66**:493–520. https://doi.org/10.1146/annurev-micro-092611-150055

3. De Maayer P et al. Some like it cold: understanding the survival strategies of psychrophiles. *EMBO Rep* 2014;**15**:508–517. https://doi.org/10.1002/embr.201338170

4. Buck M, Mehrshad M, Bertilsson S. mOTUpan: a robust Bayesian approach to leverage metagenome-assembled genomes for core-genome estimation. *NAR Genomics Bioinforma* 2022;**4**:lqac060. https://doi.org/10.1093/nargab/lqac060

5. R Core Team. R: A Language and Environment for Statistical Computing. Vienna, Austria: R Foundation for Statistical Computing, 2023.
