# Supplementary material for: Glacier influence shapes the genomic architecture of the downstream aquatic microbiome
Source: ISME Commun. 2025 May 14;5(1):ycaf076. doi: 10.1093/ismeco/ycaf076 (PMC12684718; doi:10.1093/ismeco/ycaf076)
Supplement: MAGFS_final_SI_tables_ycaf076 [file magfs_final_si_tables_ycaf076.pdf]

Supplementary Information

Glacier influence shapes the genomic architecture of the downstream aquatic microbiome

Massimo Bourquin[1,*], Hannes Peter[1], Grégoire Michoud[1], Aileen Geers[1], Susheel Bhanu Busi[2], The Vanishing Glaciers Field Team**, Tom Ian Battin[1,*]

(1) River Ecosystems Laboratory, Alpine and Polar Environmental Research Center, Ecole Polytechnique Fédérale de Lausanne, EPFL, Lausanne, Switzerland

(2) UK Centre for Ecology and Hydrology, Wallingford, United Kingdom

*corresponding authors: *Massimo.bourquin@epfl.ch*, *tom.battin@epfl.ch*

****The Vanishing Glaciers Field Team**: Mike Styllas, Matteo Tolosano, Martina Schön, Vincent de Staercke, Tyler Kohler

Supplementary Table 1 GAM models output for community averages models. resp_var = response variable, t = t-value of the linear effect, coef = coefficient of the linear effect, coef_se = standard error of the estimated coefficient, p = p-value for the linear effect, spatial_f = f-value of the spatial spline, spatial_p = p-value for the spatial spline, padj = adjusted p-value for the linear effect.

covariate	resp_var	t	coef	coef_se	p	spatial_f	spatial_p	padj
**water_temp**	mean_norm_size	1.317	0.074	0.056	0.380	1.809	0.000	1.000
**water_temp**	mean_GC	-0.763	-0.041	0.054	0.894	2.299	0.000	1.000
**water_temp**	mean_norm_gene_number	1.404	0.083	0.059	0.325	1.354	0.000	1.000
**water_temp**	mean_norm_tRNAs	1.473	0.082	0.056	0.286	2.019	0.000	1.000
**water_temp**	mean_redundancy_index	3.788	0.221	0.058	0.000	0.822	0.000	0.014
**water_temp**	mean_coding_density	0.286	0.014	0.049	1.551	3.382	0.000	1.000
**chla**	mean_norm_size	6.457	0.229	0.036	0.000	1.144	0.000	0.000
**chla**	mean_GC	-1.949	-0.077	0.039	0.107	1.980	0.000	1.000
**chla**	mean_norm_gene_number	5.190	0.198	0.038	0.000	0.908	0.000	0.000
**chla**	mean_norm_tRNAs	4.842	0.187	0.039	0.000	1.782	0.000	0.000
**chla**	mean_redundancy_index	0.889	0.038	0.042	0.751	1.044	0.000	1.000
**chla**	mean_coding_density	-2.907	-0.103	0.035	0.009	2.979	0.000	0.256

**gl_area**	mean_norm_size	-2.250	-0.119	0.053	0.052	1.749	0.000	1.000
**gl_area**	mean_GC	-0.468	-0.025	0.053	1.281	1.966	0.000	1.000
**gl_area**	mean_norm_gene_number	-2.374	-0.130	0.055	0.038	1.301	0.000	0.950
**gl_area**	mean_norm_tRNAs	-1.368	-0.075	0.055	0.347	2.001	0.000	1.000
**gl_area**	mean_redundancy_index	0.357	0.020	0.057	1.443	0.988	0.000	1.000
**gl_area**	mean_coding_density	0.403	0.019	0.048	1.375	3.399	0.000	1.000
**gl_dist**	mean_norm_size	0.612	0.027	0.044	1.083	1.744	0.000	1.000
**gl_dist**	mean_GC	-0.338	-0.015	0.043	1.472	2.100	0.000	1.000
**gl_dist**	mean_norm_gene_number	0.912	0.042	0.046	0.727	1.329	0.000	1.000
**gl_dist**	mean_norm_tRNAs	2.438	0.106	0.044	0.032	2.091	0.000	0.837
**gl_dist**	mean_redundancy_index	3.849	0.174	0.045	0.000	1.012	0.000	0.012
**gl_dist**	mean_coding_density	1.374	0.053	0.039	0.344	3.526	0.000	1.000
**gl_cov**	mean_norm_size	-1.519	-0.558	0.368	0.262	1.850	0.000	1.000
**gl_cov**	mean_GC	1.211	0.441	0.364	0.456	2.193	0.000	1.000

**gl_cov**	mean_norm_gene_number	-1.388	-0.526	0.379	0.335	1.345	0.000	1.000
**gl_cov**	mean_norm_tRNAs	-2.611	-0.969	0.371	0.020	2.005	0.000	0.564
**gl_cov**	mean_redundancy_index	-2.510	-0.946	0.377	0.026	0.849	0.000	0.714
**gl_cov**	mean_coding_density	0.237	0.078	0.332	1.626	3.276	0.000	1.000
**gl_index**	mean_norm_size	-1.478	-0.065	0.044	0.283	1.865	0.000	1.000
**gl_index**	mean_GC	0.170	0.007	0.043	1.731	2.290	0.000	1.000
**gl_index**	mean_norm_gene_number	-1.836	-0.084	0.046	0.137	1.440	0.000	1.000
**gl_index**	mean_norm_tRNAs	-2.827	-0.123	0.043	0.011	2.161	0.000	0.315
**gl_index**	mean_redundancy_index	-3.442	-0.158	0.046	0.002	0.909	0.000	0.047
**gl_index**	mean_coding_density	-1.162	-0.045	0.039	0.495	3.489	0.000	1.000

Supplementary Table 2 Environmental parameters and sample information for all samples used in this study. Column names are: mountain_range = Mountain range, date = sampling date, time = sampling time, water_temp = Streamwater temperature, gl_name = Name of the glacier upstream, lat_sp = latitude, lon_sp = longitude, ele_sp = elevation, sn_sp_dist = distance from the sampling point to the glacier, gl_sa = glacier surface area, gl_cov = coverage of the catchment by the glacier, rgi_v6 = ID of the glacier in the RGI database, glims_id = ID of the glacier in the glims database, wgms_id = ID of the glacier in the WGMS database, chla = chlorophyll-*a* concentration. Units are described in brackets.

Sample	mountain_range	date [DD.MM.YYYY]	time [HH:MM]	water_temp [C]	gl_name	lat_sp [DD]	lon_sp [DD]	ele_sp [m]	sn_sp_dist [m]	gl_sa [km2]	gl_cov [%]	gl_a [km2]	rgi_v6	glims_id	wgms_id	chla [ug g-1]
**GL1_UP_1**	Southern Alps	22.01.19	10:00	2.4	Franz Josef	-43.45	170.1753	470	397	35.51	0.7	50.53	RGI60-18.02397	G170225E43495S	899	6.2E-06
**GL1_UP_2**	Southern Alps	22.01.19	10:00	2.4	Franz Josef	-43.45	170.1753	470	397	35.51	0.7	50.53	RGI60-18.02397	G170225E43495S	899	3.76E-05
**GL1_UP_3**	Southern Alps	22.01.19	10:00	2.4	Franz Josef	-43.45	170.1753	470	397	35.51	0.7	50.53	RGI60-18.02397	G170225E43495S	899	0.000275467
**GL1_DN_1**	Southern Alps	22.01.19	14:26	3.1	Franz Josef	-43.4274	170.1734	248	2907	35.51	0.61	58.17	RGI60-18.02397	G170225E43495S	899	8.47E-05
**GL1_DN_2**	Southern Alps	22.01.19	14:26	3.1	Franz Josef	-43.4274	170.1734	248	2907	35.51	0.61	58.17	RGI60-18.02397	G170225E43495S	899	2.23E-05
**GL1_DN_3**	Southern Alps	22.01.19	14:26	3.1	Franz Josef	-43.4274	170.1734	248	2907	35.51	0.61	58.17	RGI60-18.02397	G170225E43495S	899	0.0001969
**GL2_UP_1**	Southern Alps	25.01.19	08:37	0	Victoria	-43.4976	170.139	1145	56	3.93	0.48	8.16	RGI60-18.02270	G170174E43508S	3034	0
**GL2_UP_2**	Southern Alps	25.01.19	08:37	0	Victoria	-43.4976	170.139	1145	56	3.93	0.48	8.16	RGI60-18.02270	G170174E43508S	3034	0
**GL2_UP_3**	Southern Alps	25.01.19	08:37	0	Victoria	-43.4976	170.139	1145	56	3.93	0.48	8.16	RGI60-18.02270	G170174E43508S	3034	0
**GL2_DN_1**	Southern Alps	25.01.19	11:59	1.2	Victoria	-43.4978	170.1291	1093	855	4.08	0.41	9.99	RGI60-18.02270	G170174E43508S	3034	0
**GL2_DN_2**	Southern Alps	25.01.19	11:59	1.2	Victoria	-43.4978	170.1291	1093	855	4.08	0.41	9.99	RGI60-18.02270	G170174E43508S	3034	5.57E-06
**GL2_DN_3**	Southern Alps	25.01.19	11:59	1.2	Victoria	-43.4978	170.1291	1093	855	4.08	0.41	9.99	RGI60-18.02270	G170174E43508S	3034	0
**GL3_UP_1**	Southern Alps	26.01.19	10:30	0.3	Fox	-43.5006	170.059	338	628	38.38	0.53	72.29	RGI60-18.02375	G170162E43537S	1536	0

**GL3_UP_2**	Southern Alps	26.01.19	10:30	0.3	Fox	-43.5006	170.059	338	628	38.38	0.53	72.29	RGI60-18.02375	G170162E43537S	1536	0
**GL3_UP_3**	Southern Alps	26.01.19	10:30	0.3	Fox	-43.5006	170.059	338	628	38.38	0.53	72.29	RGI60-18.02375	G170162E43537S	1536	0
**GL3_DN_1**	Southern Alps	26.01.19	14:49	4.3	Fox	-43.4869	170.0293	226	3469	38.38	0.48	80.42	RGI60-18.02375	G170162E43537S	1536	0
**GL3_DN_2**	Southern Alps	26.01.19	14:49	4.3	Fox	-43.4869	170.0293	226	3469	38.38	0.48	80.42	RGI60-18.02375	G170162E43537S	1536	0.000577333
**GL3_DN_3**	Southern Alps	26.01.19	14:49	4.3	Fox	-43.4869	170.0293	226	3469	38.38	0.48	80.42	RGI60-18.02375	G170162E43537S	1536	1.65E-05
**GL5_UP_1**	Southern Alps	29.01.19	11:47	7.7	Lancelot	-42.9249	171.5141	1325	821	0.03	0.06	0.46	RGI60-18.02895	G171502E42928S	NA	0.070815333
**GL5_UP_2**	Southern Alps	29.01.19	11:47	7.7	Lancelot	-42.9249	171.5141	1325	821	0.03	0.06	0.46	RGI60-18.02895	G171502E42928S	NA	0.150236333
**GL5_UP_3**	Southern Alps	29.01.19	11:47	7.7	Lancelot	-42.9249	171.5141	1325	821	0.03	0.06	0.46	RGI60-18.02895	G171502E42928S	NA	0.250707333
**GL5_DN_1**	Southern Alps	29.01.19	16:04	9.4	Lancelot	-42.9263	171.5149	1255	987	0.03	0.05	0.54	RGI60-18.02895	G171502E42928S	NA	0.405664333
**GL5_DN_2**	Southern Alps	29.01.19	16:04	9.4	Lancelot	-42.9263	171.5149	1255	987	0.03	0.05	0.54	RGI60-18.02895	G171502E42928S	NA	0.212072667
**GL5_DN_3**	Southern Alps	29.01.19	16:04	9.4	Lancelot	-42.9263	171.5149	1255	987	0.03	0.05	0.54	RGI60-18.02895	G171502E42928S	NA	0.155349667
**GL6_UP_1**	Southern Alps	30.01.19	13:53	8	Crow	-42.9241	171.5137	1362	462	0.51	0.69	0.75	RGI60-18.02896	G171511E42916S	23514	0.008811667
**GL6_UP_2**	Southern Alps	30.01.19	13:53	8	Crow	-42.9241	171.5137	1362	462	0.51	0.69	0.75	RGI60-18.02896	G171511E42916S	23514	0.008560333
**GL6_UP_3**	Southern Alps	30.01.19	13:53	8	Crow	-42.9241	171.5137	1362	462	0.51	0.69	0.75	RGI60-18.02896	G171511E42916S	23514	0.010128333
**GL6_DN_1**	Southern Alps	30.01.19	09:48	8	Crow	-42.9307	171.5185	1118	1293	0.57	0.19	3.01	RGI60-18.02896	G171511E42916S	23514	0.003018667
**GL6_DN_2**	Southern Alps	30.01.19	09:48	8	Crow	-42.9307	171.5185	1118	1293	0.57	0.19	3.01	RGI60-18.02896	G171511E42916S	23514	0.003021333
**GL6_DN_3**	Southern Alps	30.01.19	09:48	8	Crow	-42.9307	171.5185	1118	1293	0.57	0.19	3.01	RGI60-18.02896	G171511E42916S	23514	0.001037667
**GL7_UP_1**	Southern Alps	31.01.19	14:37	3	White	-42.9984	171.3898	1750	318	0.29	0.53	0.54	RGI60-18.02839	G171385E43000S	3037	0.000225633
**GL7_UP_2**	Southern Alps	31.01.19	14:37	3	White	-42.9984	171.3898	1750	318	0.29	0.53	0.54	RGI60-18.02839	G171385E43000S	3037	0.000179

**GL7_UP_3**	Southern Alps	31.01.19	14:37	3	White	-42.9984	171.3898	1750	318	0.29	0.53	0.54	RGI60-18.02839	G171385E43000S	3037	0.00019
**GL7_DN_1**	Southern Alps	31.01.19	17:50	3.6	White	-42.9963	171.3913	1687	583	0.31	0.41	0.76	RGI60-18.02839	G171385E43000S	3037	0.000164
**GL7_DN_2**	Southern Alps	31.01.19	17:50	3.6	White	-42.9963	171.3913	1687	583	0.31	0.41	0.76	RGI60-18.02839	G171385E43000S	3037	0.000351333
**GL7_DN_3**	Southern Alps	31.01.19	17:50	3.6	White	-42.9963	171.3913	1687	583	0.31	0.41	0.76	RGI60-18.02839	G171385E43000S	3037	3.15E-05
**GL8_UP_1**	Southern Alps	02.02.19	10:54	0.7	Marmaduke Dixon	-42.9875	171.3904	1629	16	0.62	0.44	1.42	RGI60-18.02823	G171383E42988S	NA	9.58E-05
**GL8_UP_2**	Southern Alps	02.02.19	10:54	0.7	Marmaduke Dixon	-42.9875	171.3904	1629	16	0.62	0.44	1.42	RGI60-18.02823	G171383E42988S	NA	0.0002015
**GL8_UP_3**	Southern Alps	02.02.19	10:54	0.7	Marmaduke Dixon	-42.9875	171.3904	1629	16	0.62	0.44	1.42	RGI60-18.02823	G171383E42988S	NA	0
**GL8_DN_1**	Southern Alps	02.02.19	15:15	1.3	Marmaduke Dixon	-42.9884	171.3923	1584	199	0.62	0.43	1.44	RGI60-18.02823	G171383E42988S	NA	0.0001524
**GL8_DN_2**	Southern Alps	02.02.19	15:15	1.3	Marmaduke Dixon	-42.9884	171.3923	1584	199	0.62	0.43	1.44	RGI60-18.02823	G171383E42988S	NA	0.000128333
**GL8_DN_3**	Southern Alps	02.02.19	15:15	1.3	Marmaduke Dixon	-42.9884	171.3923	1584	199	0.62	0.43	1.44	RGI60-18.02823	G171383E42988S	NA	0.000241333
**GL9_UP_1**	Southern Alps	03.02.19	10:12	3.6	Cahill	-42.9824	171.3978	1510	180	0.6	0.66	0.91	RGI60-18.02851	G171391E42978S	21936	0
**GL9_UP_2**	Southern Alps	03.02.19	10:12	3.6	Cahill	-42.9824	171.3978	1510	180	0.6	0.66	0.91	RGI60-18.02851	G171391E42978S	21936	3.5E-05
**GL9_UP_3**	Southern Alps	03.02.19	10:12	3.6	Cahill	-42.9824	171.3978	1510	180	0.6	0.66	0.91	RGI60-18.02851	G171391E42978S	21936	2.07E-06
**GL9_DN_1**	Southern Alps	03.02.19	13:44	6	Cahill	-42.9839	171.4025	1253	593	0.6	0.62	0.97	RGI60-18.02851	G171391E42978S	21936	1.85E-05
**GL9_DN_2**	Southern Alps	03.02.19	13:44	6	Cahill	-42.9839	171.4025	1253	593	0.6	0.62	0.97	RGI60-18.02851	G171391E42978S	21936	0.000140333
**GL9_DN_3**	Southern Alps	03.02.19	13:44	6	Cahill	-42.9839	171.4025	1253	593	0.6	0.62	0.97	RGI60-18.02851	G171391E42978S	21936	0
**GL10_UP_1**	Southern Alps	08.02.19	11:52	0.7	Dart	-44.4813	168.6059	1090	12	8.62	0.55	15.59	RGI60-18.00686	G168609E44455S	898	0
**GL10_UP_2**	Southern Alps	08.02.19	11:52	0.7	Dart	-44.4813	168.6059	1090	12	8.62	0.55	15.59	RGI60-18.00686	G168609E44455S	898	0.0002129
**GL10_UP_3**	Southern Alps	08.02.19	11:52	0.7	Dart	-44.4813	168.6059	1090	12	8.62	0.55	15.59	RGI60-18.00686	G168609E44455S	898	0.000442667

**GL10_DN_1**	Southern Alps	08.02.19	15:19	0.7	Dart	-44.4818	168.5979	1042	650	9.03	0.52	17.35	RGI60-18.00686	G168609E44455S	898	0
**GL10_DN_2**	Southern Alps	08.02.19	15:19	0.7	Dart	-44.4818	168.5979	1042	650	9.03	0.52	17.35	RGI60-18.00686	G168609E44455S	898	0.000336333
**GL10_DN_3**	Southern Alps	08.02.19	15:19	0.7	Dart	-44.4818	168.5979	1042	650	9.03	0.52	17.35	RGI60-18.00686	G168609E44455S	898	0.000402333
**GL11_UP_1**	Southern Alps	09.02.19	11:56	2.9	Reid	-44.4673	168.6216	1587	320	0.43	0.63	0.68	RGI60-18.00676	G168623E44461S	21691	0.010748333
**GL11_UP_2**	Southern Alps	09.02.19	11:56	2.9	Reid	-44.4673	168.6216	1587	320	0.43	0.63	0.68	RGI60-18.00676	G168623E44461S	21691	0.012437
**GL11_UP_3**	Southern Alps	09.02.19	11:56	2.9	Reid	-44.4673	168.6216	1587	320	0.43	0.63	0.68	RGI60-18.00676	G168623E44461S	21691	0.008135333
**GL11_DN_1**	Southern Alps	09.02.19	13:52	5.4	Reid	-44.4689	168.6193	1486	571	0.43	0.56	0.76	RGI60-18.00676	G168623E44461S	21691	0.033885
**GL11_DN_2**	Southern Alps	09.02.19	13:52	5.4	Reid	-44.4689	168.6193	1486	571	0.43	0.56	0.76	RGI60-18.00676	G168623E44461S	21691	0.022913
**GL11_DN_3**	Southern Alps	09.02.19	13:52	5.4	Reid	-44.4689	168.6193	1486	571	0.43	0.56	0.76	RGI60-18.00676	G168623E44461S	21691	0.021557333
**GL12_UP_1**	Southern Alps	10.02.19	12:38	6.6	Rob Roy	-44.4758	168.7268	749	864	1.97	0.8	2.48	RGI60-18.01013	G168718E44463S	21752	0.006117667
**GL12_UP_2**	Southern Alps	10.02.19	12:38	6.6	Rob Roy	-44.4758	168.7268	749	864	1.97	0.8	2.48	RGI60-18.01013	G168718E44463S	21752	0.001270333
**GL12_UP_3**	Southern Alps	10.02.19	12:38	6.6	Rob Roy	-44.4758	168.7268	749	864	1.97	0.8	2.48	RGI60-18.01013	G168718E44463S	21752	0.000815667
**GL12_DN_1**	Southern Alps	10.02.19	16:18	7	Rob Roy	-44.4803	168.7265	717	1367	1.97	0.78	2.52	RGI60-18.01013	G168718E44463S	21752	0.001556333
**GL12_DN_2**	Southern Alps	10.02.19	16:18	7	Rob Roy	-44.4803	168.7265	717	1367	1.97	0.78	2.52	RGI60-18.01013	G168718E44463S	21752	0.003362667
**GL12_DN_3**	Southern Alps	10.02.19	16:18	7	Rob Roy	-44.4803	168.7265	717	1367	1.97	0.78	2.52	RGI60-18.01013	G168718E44463S	21752	0.003565
**GL13_UP_1**	Southern Alps	13.02.19	13:20	0.7	Brewster	-44.0819	169.4317	1699	18	1.81	0.57	3.2	RGI60-18.01130	G169437E44072S	1597	0.0155
**GL13_UP_2**	Southern Alps	13.02.19	13:20	0.7	Brewster	-44.0819	169.4317	1699	18	1.81	0.57	3.2	RGI60-18.01130	G169437E44072S	1597	0.011838333
**GL13_UP_3**	Southern Alps	13.02.19	13:20	0.7	Brewster	-44.0819	169.4317	1699	18	1.81	0.57	3.2	RGI60-18.01130	G169437E44072S	1597	0.024356667
**GL13_DN_1**	Southern Alps	13.02.19	16:20	0.9	Brewster	-44.0838	169.4305	1655	241	1.81	0.53	3.41	RGI60-18.01130	G169437E44072S	1597	0.002597333

**GL13_DN_2**	Southern Alps	13.02.19	16:20	0.9	Brewster	-44.0838	169.4305	1655	241	1.81	0.53	3.41	RGI60-18.01130	G169437E44072S	1597	0.008608333
**GL13_DN_3**	Southern Alps	13.02.19	16:20	0.9	Brewster	-44.0838	169.4305	1655	241	1.81	0.53	3.41	RGI60-18.01130	G169437E44072S	1597	0.013549
**GL14_UP_1**	Southern Alps	16.02.19	10:12	3.4	Mc Pherson	-44.7565	167.9857	1096	309	0.3	0.32	0.93	RGI60-18.00367	G167987E44758S	NA	0.16609
**GL14_UP_2**	Southern Alps	16.02.19	10:12	3.4	Mc Pherson	-44.7565	167.9857	1096	309	0.3	0.32	0.93	RGI60-18.00367	G167987E44758S	NA	0.170620333
**GL14_UP_3**	Southern Alps	16.02.19	10:12	3.4	Mc Pherson	-44.7565	167.9857	1096	309	0.3	0.32	0.93	RGI60-18.00367	G167987E44758S	NA	0.113155667
**GL14_DN_1**	Southern Alps	16.02.19	13:18	4.9	Mc Pherson	-44.7571	167.9865	1071	402	0.3	0.26	1.15	RGI60-18.00367	G167987E44758S	NA	0.038028333
**GL14_DN_2**	Southern Alps	16.02.19	13:18	4.9	Mc Pherson	-44.7571	167.9865	1071	402	0.3	0.26	1.15	RGI60-18.00367	G167987E44758S	NA	0.108576667
**GL14_DN_3**	Southern Alps	16.02.19	13:18	4.9	Mc Pherson	-44.7571	167.9865	1071	402	0.3	0.26	1.15	RGI60-18.00367	G167987E44758S	NA	0.066757
**GL15_UP_1**	Southern Alps	17.02.19	08:52	1.4	Age	-44.6121	168.0218	1288	27	1.6	0.65	2.46	RGI60-18.00179	G168020E44606S	21646	0.002285667
**GL15_UP_2**	Southern Alps	17.02.19	08:52	1.4	Age	-44.6121	168.0218	1288	27	1.6	0.65	2.46	RGI60-18.00179	G168020E44606S	21646	0.004701
**GL15_UP_3**	Southern Alps	17.02.19	08:52	1.4	Age	-44.6121	168.0218	1288	27	1.6	0.65	2.46	RGI60-18.00179	G168020E44606S	21646	0.002838
**GL15_DN_1**	Southern Alps	17.02.19	05:31	1.6	Age	-44.6121	168.0214	1259	57	1.6	0.65	2.46	RGI60-18.00179	G168020E44606S	21646	0.086444
**GL15_DN_2**	Southern Alps	17.02.19	05:31	1.6	Age	-44.6121	168.0214	1259	57	1.6	0.65	2.46	RGI60-18.00179	G168020E44606S	21646	0.010362
**GL15_DN_3**	Southern Alps	17.02.19	05:31	1.6	Age	-44.6121	168.0214	1259	57	1.6	0.65	2.46	RGI60-18.00179	G168020E44606S	21646	0.010365333
**GL16_UP_1**	Southern Alps	22.02.19	13:53	5.7	Birch Creek	-43.794	170.0643	1320	907	0.15	0.1	1.44	RGI60-18.01559	G170055E43788S	NA	0.071213333
**GL16_UP_2**	Southern Alps	22.02.19	13:53	5.7	Birch Creek	-43.794	170.0643	1320	907	0.15	0.1	1.44	RGI60-18.01559	G170055E43788S	NA	0.062834667
**GL16_UP_3**	Southern Alps	22.02.19	13:53	5.7	Birch Creek	-43.794	170.0643	1320	907	0.15	0.1	1.44	RGI60-18.01559	G170055E43788S	NA	0.039197667
**GL16_DN_1**	Southern Alps	22.02.19	17:09	4.9	Birch Creek	-43.7946	170.0674	1206	1164	0.15	0.07	1.95	RGI60-18.01559	G170055E43788S	NA	0.044238667
**GL16_DN_2**	Southern Alps	22.02.19	17:09	4.9	Birch Creek	-43.7946	170.0674	1206	1164	0.15	0.07	1.95	RGI60-18.01559	G170055E43788S	NA	0.046043333

**GL16_DN_3**	Southern Alps	22.02.19	17:09	4.9	Birch Creek	-43.7946	170.0674	1206	1164	0.15	0.07	1.95	RGI60-18.01559	G170055E43788S	NA	0.049379667
**GL17_UP_1**	Southern Alps	23.02.19	11:50	5.9	Tewaewae	-43.6891	170.0822	1236	598	0.47	0.6	0.78	RGI60-18.01881	G170073E43683S	2276	0.003182333
**GL17_UP_2**	Southern Alps	23.02.19	11:50	5.9	Tewaewae	-43.6891	170.0822	1236	598	0.47	0.6	0.78	RGI60-18.01881	G170073E43683S	2276	0.002079667
**GL17_UP_3**	Southern Alps	23.02.19	11:50	5.9	Tewaewae	-43.6891	170.0822	1236	598	0.47	0.6	0.78	RGI60-18.01881	G170073E43683S	2276	0.002729667
**GL17_DN_1**	Southern Alps	23.02.19	15:38	6.9	Tewaewae	-43.6936	170.0849	1006	1145	0.49	0.43	1.13	RGI60-18.01881	G170073E43683S	2276	0.002893333
**GL17_DN_2**	Southern Alps	23.02.19	15:38	6.9	Tewaewae	-43.6936	170.0849	1006	1145	0.49	0.43	1.13	RGI60-18.01881	G170073E43683S	2276	0.001861
**GL17_DN_3**	Southern Alps	23.02.19	15:38	6.9	Tewaewae	-43.6936	170.0849	1006	1145	0.49	0.43	1.13	RGI60-18.01881	G170073E43683S	2276	0.001959667
**GL18_UP_1**	Southern Alps	26.02.19	11:07	4.2	Charity	-43.8169	169.9252	1208	381	0.41	0.27	1.5	RGI60-18.01835	G169924E43808S	21828	0
**GL18_UP_2**	Southern Alps	26.02.19	11:07	4.2	Charity	-43.8169	169.9252	1208	381	0.41	0.27	1.5	RGI60-18.01835	G169924E43808S	21828	2.38E-05
**GL18_UP_3**	Southern Alps	26.02.19	11:07	4.2	Charity	-43.8169	169.9252	1208	381	0.41	0.27	1.5	RGI60-18.01835	G169924E43808S	21828	0.000328
**GL18_DN_1**	Southern Alps	26.02.19	14:50	5.5	Charity	-43.8184	169.9279	1113	650	0.41	0.26	1.61	RGI60-18.01835	G169924E43808S	21828	0
**GL18_DN_2**	Southern Alps	26.02.19	14:50	5.5	Charity	-43.8184	169.9279	1113	650	0.41	0.26	1.61	RGI60-18.01835	G169924E43808S	21828	0
**GL18_DN_3**	Southern Alps	26.02.19	14:50	5.5	Charity	-43.8184	169.9279	1113	650	0.41	0.26	1.61	RGI60-18.01835	G169924E43808S	21828	7.76E-05
**GL19_UP_1**	Southern Alps	27.02.19	09:37	0.1	Richardson	-43.8172	169.9336	1146	53	2.45	0.23	10.5	RGI60-18.01958	G169947E43804S	1574	0
**GL19_UP_2**	Southern Alps	27.02.19	09:37	0.1	Richardson	-43.8172	169.9336	1146	53	2.45	0.23	10.5	RGI60-18.01958	G169947E43804S	1574	0
**GL19_UP_3**	Southern Alps	27.02.19	09:37	0.1	Richardson	-43.8172	169.9336	1146	53	2.45	0.23	10.5	RGI60-18.01958	G169947E43804S	1574	0
**GL19_DN_1**	Southern Alps	27.02.19	16:07	1.8	Richardson	-43.8239	169.9231	1097	1179	2.86	0.19	15.19	RGI60-18.01958	G169947E43804S	1574	0
**GL19_DN_2**	Southern Alps	27.02.19	16:07	1.8	Richardson	-43.8239	169.9231	1097	1179	2.86	0.19	15.19	RGI60-18.01958	G169947E43804S	1574	0
**GL19_DN_3**	Southern Alps	27.02.19	16:07	1.8	Richardson	-43.8239	169.9231	1097	1179	2.86	0.19	15.19	RGI60-18.01958	G169947E43804S	1574	0

**GL20_UP_1**	Southern Alps	01.03.19	12:36	2.6	Mawson	-43.418	170.5	1448	96	0.8	0.49	1.64	RGI60-18.02348	G170508E43421S	21883	0
**GL20_UP_2**	Southern Alps	01.03.19	12:36	2.6	Mawson	-43.418	170.5	1448	96	0.8	0.49	1.64	RGI60-18.02348	G170508E43421S	21883	0
**GL20_UP_3**	Southern Alps	01.03.19	12:36	2.6	Mawson	-43.418	170.5	1448	96	0.8	0.49	1.64	RGI60-18.02348	G170508E43421S	21883	0.000336333
**GL20_DN_1**	Southern Alps	01.03.19	16:45	5.3	Mawson	-43.4074	170.4994	1004	1275	0.8	0.26	3.07	RGI60-18.02348	G170508E43421S	21883	0.000244667
**GL20_DN_2**	Southern Alps	01.03.19	16:45	5.3	Mawson	-43.4074	170.4994	1004	1275	0.8	0.26	3.07	RGI60-18.02348	G170508E43421S	21883	0.000935667
**GL20_DN_3**	Southern Alps	01.03.19	16:45	5.3	Mawson	-43.4074	170.4994	1004	1275	0.8	0.26	3.07	RGI60-18.02348	G170508E43421S	21883	9.88E-05
**GL21_UP_1**	Southern Alps	02.03.19	10:12	3.4	Shackleton	-43.4043	170.5064	1138	145	2.23	0.56	4.02	RGI60-18.02298	G170521E43401S	2285	0.001400667
**GL21_UP_2**	Southern Alps	02.03.19	10:12	3.4	Shackleton	-43.4043	170.5064	1138	145	2.23	0.56	4.02	RGI60-18.02298	G170521E43401S	2285	0.002305333
**GL21_UP_3**	Southern Alps	02.03.19	10:12	3.4	Shackleton	-43.4043	170.5064	1138	145	2.23	0.56	4.02	RGI60-18.02298	G170521E43401S	2285	0.001926
**GL21_DN_1**	Southern Alps	02.03.19	13:43	2.7	Shackleton	-43.406	170.501	982	621	2.38	0.44	5.42	RGI60-18.02298	G170521E43401S	2285	9E-05
**GL21_DN_2**	Southern Alps	02.03.19	13:43	2.7	Shackleton	-43.406	170.501	982	621	2.38	0.44	5.42	RGI60-18.02298	G170521E43401S	2285	6.87E-05
**GL21_DN_3**	Southern Alps	02.03.19	13:43	2.7	Shackleton	-43.406	170.501	982	621	2.38	0.44	5.42	RGI60-18.02298	G170521E43401S	2285	0.000172333
**GL22_UP_1**	European Alps	25.06.19	09:13	0.2	Valsorey	45.9166	7.2669	2441	76	1.97	0.43	4.58	RGI60-11.02927	G007257E45892N	365	0.007273667
**GL22_UP_2**	European Alps	25.06.19	09:13	0.2	Valsorey	45.9166	7.2669	2441	76	1.97	0.43	4.58	RGI60-11.02927	G007257E45892N	365	1.42E-05
**GL22_UP_3**	European Alps	25.06.19	09:13	0.2	Valsorey	45.9166	7.2669	2441	76	1.97	0.43	4.58	RGI60-11.02927	G007257E45892N	365	0.000532667
**GL22_DN_1**	European Alps	25.06.19	13:36	4.7	Valsorey	45.9208	7.257	2387	973	1.97	0.24	8.12	RGI60-11.02927	G007257E45892N	365	0.001584667
**GL22_DN_2**	European Alps	25.06.19	13:36	4.7	Valsorey	45.9208	7.257	2387	973	1.97	0.24	8.12	RGI60-11.02927	G007257E45892N	365	0.002420667
**GL22_DN_3**	European Alps	25.06.19	13:36	4.7	Valsorey	45.9208	7.257	2387	973	1.97	0.24	8.12	RGI60-11.02927	G007257E45892N	365	0.042358333
**GL23_UP_1**	European Alps	03.06.20	12:46	0.3	Furgg	45.979	7.6868	2735	15	3.01	0.84	3.56	RGI60-11.02819	G007696E45965N	5490	0.000545633

**GL23_UP_2**	European Alps	03.06.20	12:46	0.3	Furgg	45.979	7.6868	2735	15	3.01	0.84	3.56	RGI60-11.02819	G007696E45965N	5490	0.001948
**GL23_UP_3**	European Alps	03.06.20	12:46	0.3	Furgg	45.979	7.6868	2735	15	3.01	0.84	3.56	RGI60-11.02819	G007696E45965N	5490	0.000942667
**GL23_DN_1**	European Alps	03.06.20	15:18	2.4	Furgg	45.9845	7.6928	2680	778	4.52	0.59	7.71	RGI60-11.02819	G007696E45965N	5490	0.000395667
**GL23_DN_2**	European Alps	03.06.20	15:18	2.4	Furgg	45.9845	7.6928	2680	778	4.52	0.59	7.71	RGI60-11.02819	G007696E45965N	5490	0.000349667
**GL23_DN_3**	European Alps	03.06.20	15:18	2.4	Furgg	45.9845	7.6928	2680	778	4.52	0.59	7.71	RGI60-11.02819	G007696E45965N	5490	0.001693667
**GL24_UP_1**	European Alps	04.06.20	09:10	0	Zmutt	46.0016	7.6511	2317	31	14.15	0.54	26.02	RGI60-11.02739	G007639E45977N	390	0.000656333
**GL24_UP_2**	European Alps	04.06.20	09:10	0	Zmutt	46.0016	7.6511	2317	31	14.15	0.54	26.02	RGI60-11.02739	G007639E45977N	390	0.01942
**GL24_UP_3**	European Alps	04.06.20	09:10	0	Zmutt	46.0016	7.6511	2317	31	14.15	0.54	26.02	RGI60-11.02739	G007639E45977N	390	0.020264
**GL24_DN_1**	European Alps	04.06.20	11:50	1.9	Zmutt	46.003	7.6573	2247	536	14.58	0.53	27.29	RGI60-11.02739	G007639E45977N	390	0.005411667
**GL24_DN_2**	European Alps	04.06.20	11:50	1.9	Zmutt	46.003	7.6573	2247	536	14.58	0.53	27.29	RGI60-11.02739	G007639E45977N	390	0.072743667
**GL24_DN_3**	European Alps	04.06.20	11:50	1.9	Zmutt	46.003	7.6573	2247	536	14.58	0.53	27.29	RGI60-11.02739	G007639E45977N	390	0.006101667
**GL25_UP_1**	European Alps	05.06.20	11:00	0.5	Findelen	46.0108	7.8263	2557	158	15.38	0.75	20.51	RGI60-11.02773	G007880E45990N	389	0.005571333
**GL25_UP_2**	European Alps	05.06.20	11:00	0.5	Findelen	46.0108	7.8263	2557	158	15.38	0.75	20.51	RGI60-11.02773	G007880E45990N	389	0.007291
**GL25_UP_3**	European Alps	05.06.20	11:00	0.5	Findelen	46.0108	7.8263	2557	158	15.38	0.75	20.51	RGI60-11.02773	G007880E45990N	389	0.001326333
**GL25_DN_1**	European Alps	05.06.20	14:30	0.7	Findelen	46.0105	7.8199	2508	652	15.38	0.73	20.97	RGI60-11.02773	G007880E45990N	389	0.000745
**GL25_DN_2**	European Alps	05.06.20	14:30	0.7	Findelen	46.0105	7.8199	2508	652	15.38	0.73	20.97	RGI60-11.02773	G007880E45990N	389	0.000794667
**GL25_DN_3**	European Alps	05.06.20	14:30	0.7	Findelen	46.0105	7.8199	2508	652	15.38	0.73	20.97	RGI60-11.02773	G007880E45990N	389	0.001719333
**GL26_UP_1**	European Alps	06.06.20	11:30	0.7	Längflue-N	46.031	7.8515	2916	288	0.86	0.72	1.19	RGI60-11.02742	G007860E46022N	NA	0.119485667
**GL26_UP_2**	European Alps	06.06.20	11:30	0.7	Längflue-N	46.031	7.8515	2916	288	0.86	0.72	1.19	RGI60-11.02742	G007860E46022N	NA	0.111111333

**GL26_UP_3**	European Alps	06.06.20	11:30	0.7	Längflue-N	46.031	7.8515	2916	288	0.86	0.72	1.19	RGI60-11.02742	G007860E46022N	NA	0.063384667
**GL26_DN_1**	European Alps	06.06.20	14:31	3.4	Längflue-N	46.0318	7.8495	2876	471	0.86	0.64	1.34	RGI60-11.02742	G007860E46022N	NA	0.337788333
**GL26_DN_2**	European Alps	06.06.20	14:31	3.4	Längflue-N	46.0318	7.8495	2876	471	0.86	0.64	1.34	RGI60-11.02742	G007860E46022N	NA	0.44469
**GL26_DN_3**	European Alps	06.06.20	14:31	3.4	Längflue-N	46.0318	7.8495	2876	471	0.86	0.64	1.34	RGI60-11.02742	G007860E46022N	NA	0.303640333
**GL27_UP_1**	European Alps	08.06.20	10:12	0.3	Arolla (Bas)	45.9901	7.496	2265	125	4.77	0.78	6.08	RGI60-11.02787	G007490E45965N	377	0.013854333
**GL27_UP_2**	European Alps	08.06.20	10:12	0.3	Arolla (Bas)	45.9901	7.496	2265	125	4.77	0.78	6.08	RGI60-11.02787	G007490E45965N	377	0.005714
**GL27_UP_3**	European Alps	08.06.20	10:12	0.3	Arolla (Bas)	45.9901	7.496	2265	125	4.77	0.78	6.08	RGI60-11.02787	G007490E45965N	377	0.006970333
**GL27_DN_1**	European Alps	08.06.20	14:48	3.1	Arolla (Bas)	46.0006	7.4921	2112	1330	10.4	0.4	25.92	RGI60-11.02787	G007490E45965N	377	0.008597
**GL27_DN_2**	European Alps	08.06.20	14:48	3.1	Arolla (Bas)	46.0006	7.4921	2112	1330	10.4	0.4	25.92	RGI60-11.02787	G007490E45965N	377	0.014540333
**GL27_DN_3**	European Alps	08.06.20	14:48	3.1	Arolla (Bas)	46.0006	7.4921	2112	1330	10.4	0.4	25.92	RGI60-11.02787	G007490E45965N	377	0.002167667
**GL28_UP_1**	European Alps	09.06.20	08:16	0	Tsidjiore Nouve	46.016	7.4692	2277	76	2.77	0.6	4.62	RGI60-11.02755	G007450E45997N	376	0.001927
**GL28_UP_2**	European Alps	09.06.20	08:16	0	Tsidjiore Nouve	46.016	7.4692	2277	76	2.77	0.6	4.62	RGI60-11.02755	G007450E45997N	376	0.000647667
**GL28_UP_3**	European Alps	09.06.20	08:16	0	Tsidjiore Nouve	46.016	7.4692	2277	76	2.77	0.6	4.62	RGI60-11.02755	G007450E45997N	376	0.001127
**GL28_DN_1**	European Alps	10.06.20	11:05	2.1	Tsidjiore Nouve	46.0188	7.4736	2148	537	2.77	0.58	4.76	RGI60-11.02755	G007450E45997N	376	0.004775667
**GL28_DN_2**	European Alps	10.06.20	11:05	2.1	Tsidjiore Nouve	46.0188	7.4736	2148	537	2.77	0.58	4.76	RGI60-11.02755	G007450E45997N	376	0.006204333
**GL28_DN_3**	European Alps	10.06.20	11:05	2.1	Tsidjiore Nouve	46.0188	7.4736	2148	537	2.77	0.58	4.76	RGI60-11.02755	G007450E45997N	376	0.023702333
**GL29_UP_1**	European Alps	11.06.20	10:30	0.6	Mont Mine	46.0396	7.5505	2085	35	10.48	0.64	16.49	RGI60-11.02709	G007553E45994N	378	0.001602667
**GL29_UP_2**	European Alps	11.06.20	10:30	0.6	Mont Mine	46.0396	7.5505	2085	35	10.48	0.64	16.49	RGI60-11.02709	G007553E45994N	378	0.000208
**GL29_UP_3**	European Alps	11.06.20	10:30	0.6	Mont Mine	46.0396	7.5505	2085	35	10.48	0.64	16.49	RGI60-11.02709	G007553E45994N	378	0.001210333

**GL29_DN_1**	European Alps	11.06.20	13:50	1.7	Mont Mine	46.0437	7.5535	1977	547	10.48	0.62	16.95	RGI60-11.02709	G007553E45994N	378	0.014284667
**GL29_DN_2**	European Alps	11.06.20	13:50	1.7	Mont Mine	46.0437	7.5535	1977	547	10.48	0.62	16.95	RGI60-11.02709	G007553E45994N	378	0.00392
**GL29_DN_3**	European Alps	11.06.20	13:50	1.7	Mont Mine	46.0437	7.5535	1977	547	10.48	0.62	16.95	RGI60-11.02709	G007553E45994N	378	0.003433667
**GL30_UP_1**	European Alps	30.06.20	11:11	4.8	Hohlaub-N	46.1446	7.9939	3081	205	0.33	0.64	0.52	RGI60-11.02526	G008004E46144N	5434	0.015691667
**GL30_UP_2**	European Alps	30.06.20	11:11	4.8	Hohlaub-N	46.1446	7.9939	3081	205	0.33	0.64	0.52	RGI60-11.02526	G008004E46144N	5434	0.006014667
**GL30_UP_3**	European Alps	30.06.20	11:11	4.8	Hohlaub-N	46.1446	7.9939	3081	205	0.33	0.64	0.52	RGI60-11.02526	G008004E46144N	5434	0.006263
**GL30_DN_1**	European Alps	30.06.20	13:00	2.8	Hohlaub-N	46.1449	7.9921	2983	345	0.33	0.63	0.53	RGI60-11.02526	G008004E46144N	5434	0.009701
**GL30_DN_2**	European Alps	30.06.20	13:00	2.8	Hohlaub-N	46.1449	7.9921	2983	345	0.33	0.63	0.53	RGI60-11.02526	G008004E46144N	5434	0.006833333
**GL30_DN_3**	European Alps	30.06.20	13:00	2.8	Hohlaub-N	46.1449	7.9921	2983	345	0.33	0.63	0.53	RGI60-11.02526	G008004E46144N	5434	0.006349667
**GL31_UP_1**	European Alps	01.07.20	11:15	0.4	Schwarzberg	46.0265	7.939	2662	72	5.84	0.72	8.13	RGI60-11.02746	G007922E46000N	395	0.016622333
**GL31_UP_2**	European Alps	01.07.20	11:15	0.4	Schwarzberg	46.0265	7.939	2662	72	5.84	0.72	8.13	RGI60-11.02746	G007922E46000N	395	0.004452333
**GL31_UP_3**	European Alps	01.07.20	11:15	0.4	Schwarzberg	46.0265	7.939	2662	72	5.84	0.72	8.13	RGI60-11.02746	G007922E46000N	395	0.008858
**GL31_DN_1**	European Alps	01.07.20	13:30	1.8	Schwarzberg	46.0276	7.9397	2659	205	5.84	0.68	8.6	RGI60-11.02746	G007922E46000N	395	0.017418
**GL31_DN_2**	European Alps	01.07.20	13:30	1.8	Schwarzberg	46.0276	7.9397	2659	205	5.84	0.68	8.6	RGI60-11.02746	G007922E46000N	395	0.005623333
**GL31_DN_3**	European Alps	01.07.20	13:30	1.8	Schwarzberg	46.0276	7.9397	2659	205	5.84	0.68	8.6	RGI60-11.02746	G007922E46000N	395	0.001099
**GL32_UP_1**	Southwest Greenland	15.07.19	20:50	0.1	"Nuuk east"	64.1383	-51.1768	613	15	0.16	0.18	0.86	RGI60-05.07197	G308826E64132N	NA	0.00828
**GL32_UP_2**	Southwest Greenland	15.07.19	20:50	0.1	"Nuuk east"	64.1383	-51.1768	613	15	0.16	0.18	0.86	RGI60-05.07197	G308826E64132N	NA	0.025584667
**GL32_UP_3**	Southwest Greenland	15.07.19	20:50	0.1	"Nuuk east"	64.1383	-51.1768	613	15	0.16	0.18	0.86	RGI60-05.07197	G308826E64132N	NA	0.025738333
**GL32_DN_1**	Southwest Greenland	16.07.19	20:18	6.3	"Nuuk east"	64.1395	-51.1732	550	234	0.16	0.16	0.96	RGI60-05.07197	G308826E64132N	NA	0.015709667

**GL32_DN_2**	Southwest Greenland	16.07.19	20:18	6.3	"Nuuk east"	64.1395	-51.1732	550	234	0.16	0.16	0.96	RGI60-05.07197	G308826E64132N	NA	0.019202
**GL32_DN_3**	Southwest Greenland	16.07.19	20:18	6.3	"Nuuk east"	64.1395	-51.1732	550	234	0.16	0.16	0.96	RGI60-05.07197	G308826E64132N	NA	0.015037
**GL33_UP_1**	Southwest Greenland	16.07.19	12:00	0.1	"Nuuk middle"	64.1406	-51.1926	668	10	0.55	0.57	0.96	RGI60-05.07202	G308801E64139N	NA	0.001384
**GL33_UP_2**	Southwest Greenland	16.07.19	12:00	0.1	"Nuuk middle"	64.1406	-51.1926	668	10	0.55	0.57	0.96	RGI60-05.07202	G308801E64139N	NA	0.004201667
**GL33_UP_3**	Southwest Greenland	16.07.19	12:00	0.1	"Nuuk middle"	64.1406	-51.1926	668	10	0.55	0.57	0.96	RGI60-05.07202	G308801E64139N	NA	0.002988333
**GL33_DN_1**	Southwest Greenland	16.07.19	16:00	2.2	"Nuuk middle"	64.1418	-51.19	588	196	0.56	0.55	1.02	RGI60-05.07202	G308801E64139N	NA	0.057034333
**GL33_DN_2**	Southwest Greenland	16.07.19	16:00	2.2	"Nuuk middle"	64.1418	-51.19	588	196	0.56	0.55	1.02	RGI60-05.07202	G308801E64139N	NA	0.083216333
**GL33_DN_3**	Southwest Greenland	16.07.19	16:00	2.2	"Nuuk middle"	64.1418	-51.19	588	196	0.56	0.55	1.02	RGI60-05.07202	G308801E64139N	NA	0.051829
**GL34_UP_1**	Southwest Greenland	17.07.19	14:37	0.3	"Nuuk west"	64.1514	-51.2181	557	23	1.49	0.69	2.15	RGI60-05.07208	G308763E64146N	NA	0.002433333
**GL34_UP_2**	Southwest Greenland	17.07.19	14:37	0.3	"Nuuk west"	64.1514	-51.2181	557	23	1.49	0.69	2.15	RGI60-05.07208	G308763E64146N	NA	0.003629667
**GL34_UP_3**	Southwest Greenland	17.07.19	14:37	0.3	"Nuuk west"	64.1514	-51.2181	557	23	1.49	0.69	2.15	RGI60-05.07208	G308763E64146N	NA	0.007994333
**GL34_DN_1**	Southwest Greenland	17.07.19	17:30	2.6	"Nuuk west"	64.1479	-51.1958	473	1174	1.54	0.25	6.27	RGI60-05.07208	G308763E64146N	NA	0.014012
**GL34_DN_2**	Southwest Greenland	17.07.19	17:30	2.6	"Nuuk west"	64.1479	-51.1958	473	1174	1.54	0.25	6.27	RGI60-05.07208	G308763E64146N	NA	0.005614333
**GL34_DN_3**	Southwest Greenland	17.07.19	17:30	2.6	"Nuuk west"	64.1479	-51.1958	473	1174	1.54	0.25	6.27	RGI60-05.07208	G308763E64146N	NA	0.004548667
**GL35_UP_1**	Southwest Greenland	18.07.19	14:00	1.2	"Nuuk east"	64.166	-51.0694	757	4	1	0.68	1.46	RGI60-05.07210	G308916E64163N	NA	0.004911667
**GL35_UP_2**	Southwest Greenland	18.07.19	14:00	1.2	"Nuuk east"	64.166	-51.0694	757	4	1	0.68	1.46	RGI60-05.07210	G308916E64163N	NA	0.000245
**GL35_UP_3**	Southwest Greenland	18.07.19	14:00	1.2	"Nuuk east"	64.166	-51.0694	757	4	1	0.68	1.46	RGI60-05.07210	G308916E64163N	NA	0.003338333
**GL35_DN_1**	Southwest Greenland	18.07.19	13:00	2.8	"Nuuk east"	64.1626	-51.059	547	633	1	0.6	1.66	RGI60-05.07210	G308916E64163N	NA	0.020087667
**GL35_DN_2**	Southwest Greenland	18.07.19	13:00	2.8	"Nuuk east"	64.1626	-51.059	547	633	1	0.6	1.66	RGI60-05.07210	G308916E64163N	NA	0.014171333

**GL35_DN_3**	Southwest Greenland	18.07.19	13:00	2.8	"Nuuk east"	64.16<br>26	-<br>51.05<br>9	547	633	1	0.6	1.66	RGI60<br>-<br>05.07<br>210	G308916E64<br>163N	NA	0.009669<br>667
**GL36_UP_1**	Southwest Greenland	21.07.19	12:55	7.3	Aajuitsup Sermia	64.12<br>11	-<br>51.48<br>05	523	286	0.61	0.47	1.3	RGI60<br>-<br>05.07<br>199	G308534E64<br>118N	NA	0.018958<br>333
**GL36_UP_2**	Southwest Greenland	21.07.19	12:55	7.3	Aajuitsup Sermia	64.12<br>11	-<br>51.48<br>05	523	286	0.61	0.47	1.3	RGI60<br>-<br>05.07<br>199	G308534E64<br>118N	NA	0.060896<br>667
**GL36_UP_3**	Southwest Greenland	21.07.19	12:55	7.3	Aajuitsup Sermia	64.12<br>11	-<br>51.48<br>05	523	286	0.61	0.47	1.3	RGI60<br>-<br>05.07<br>199	G308534E64<br>118N	NA	0.025469
**GL36_DN_1**	Southwest Greenland	21.07.19	16:50	9.2	Aajuitsup Sermia	64.12<br>62	-<br>51.49<br>01	388	1027	0.63	0.33	1.92	RGI60<br>-<br>05.07<br>199	G308534E64<br>118N	NA	0.030085<br>667
**GL36_DN_2**	Southwest Greenland	21.07.19	16:50	9.2	Aajuitsup Sermia	64.12<br>62	-<br>51.49<br>01	388	1027	0.63	0.33	1.92	RGI60<br>-<br>05.07<br>199	G308534E64<br>118N	NA	0.061720<br>333
**GL36_DN_3**	Southwest Greenland	21.07.19	16:50	9.2	Aajuitsup Sermia	64.12<br>62	-<br>51.49<br>01	388	1027	0.63	0.33	1.92	RGI60<br>-<br>05.07<br>199	G308534E64<br>118N	NA	0.067941
**GL37_UP_1**	Southwest Greenland	22.07.19	12:31	0.4	Teqqiinngallip Sermia	64.13<br>52	-<br>51.47<br>07	439	111	0.56	0.62	0.9	RGI60<br>-<br>05.07<br>207	G308542E64<br>131N	NA	0.011044<br>333
**GL37_UP_2**	Southwest Greenland	22.07.19	12:31	0.4	Teqqiinngallip Sermia	64.13<br>52	-<br>51.47<br>07	439	111	0.56	0.62	0.9	RGI60<br>-<br>05.07<br>207	G308542E64<br>131N	NA	0.007492<br>667
**GL37_UP_3**	Southwest Greenland	22.07.19	12:31	0.4	Teqqiinngallip Sermia	64.13<br>52	-<br>51.47<br>07	439	111	0.56	0.62	0.9	RGI60<br>-<br>05.07<br>207	G308542E64<br>131N	NA	0.003624
**GL37_DN_1**	Southwest Greenland	22.07.19	16:12	4.6	Teqqiinngallip Sermia	64.14<br>08	-<br>51.47<br>55	249	773	0.56	0.74	0.76	RGI60<br>-<br>05.07<br>207	G308542E64<br>131N	NA	0.187054<br>667
**GL37_DN_2**	Southwest Greenland	22.07.19	16:12	4.6	Teqqiinngallip Sermia	64.14<br>08	-<br>51.47<br>55	249	773	0.56	0.74	0.76	RGI60<br>-<br>05.07<br>207	G308542E64<br>131N	NA	0.044527
**GL37_DN_3**	Southwest Greenland	22.07.19	16:12	4.6	Teqqiinngallip Sermia	64.14<br>08	-<br>51.47<br>55	249	773	0.56	0.74	0.76	RGI60<br>-<br>05.07<br>207	G308542E64<br>131N	NA	0.062654
**GL38_UP_1**	Southwest Greenland	29.07.19	11:40	0.3	Lyngmarks brae	69.29<br>04	-<br>53.57<br>1	737	23	1.14	1	1.14	RGI60<br>-<br>05.00<br>625	G306419E69<br>295N	3737-<br>28	0.025518<br>333
**GL38_UP_2**	Southwest Greenland	29.07.19	11:40	0.3	Lyngmarks brae	69.29<br>04	-<br>53.57<br>1	737	23	1.14	1	1.14	RGI60<br>-<br>05.00<br>625	G306419E69<br>295N	3737-<br>28	0.006757<br>667
**GL38_UP_3**	Southwest Greenland	29.07.19	11:40	0.3	Lyngmarks brae	69.29<br>04	-<br>53.57<br>1	737	23	1.14	1	1.14	RGI60<br>-<br>05.00<br>625	G306419E69<br>295N	3737-<br>28	0.00209
**GL38_DN_1**	Southwest Greenland	29.07.19	15:25	5.5	Lyngmarks brae	69.28<br>15	-<br>53.53<br>72	444	1689	1.21	0.32	3.76	RGI60<br>-<br>05.00<br>625	G306419E69<br>295N	3737-<br>28	0.017746<br>333
**GL38_DN_2**	Southwest Greenland	29.07.19	15:25	5.5	Lyngmarks brae	69.28<br>15	-<br>53.53<br>72	444	1689	1.21	0.32	3.76	RGI60<br>-<br>05.00<br>625	G306419E69<br>295N	3737-<br>28	0.002993<br>333
**GL38_DN_3**	Southwest Greenland	29.07.19	15:25	5.5	Lyngmarks brae	69.28<br>15	-<br>53.53<br>72	444	1689	1.21	0.32	3.76	RGI60<br>-<br>05.00<br>625	G306419E69<br>295N	3737-<br>28	0.001370<br>333

**GL39_UP_1**	Southwest Greenland	30.07.19	12:06	0.2	Chamberlin	69.3204	-53.5282	468	40	4.25	0.89	4.76	RGI60-05.00623	G306428E69317N	3735	0.002131
**GL39_UP_2**	Southwest Greenland	30.07.19	12:06	0.2	Chamberlin	69.3204	-53.5282	468	40	4.25	0.89	4.76	RGI60-05.00623	G306428E69317N	3735	0.001390667
**GL39_UP_3**	Southwest Greenland	30.07.19	12:06	0.2	Chamberlin	69.3204	-53.5282	468	40	4.25	0.89	4.76	RGI60-05.00623	G306428E69317N	3735	0.001088
**GL39_DN_1**	Southwest Greenland	30.07.19	15:19	2.3	Chamberlin	69.321	-53.4954	332	1306	4.77	0.47	10.21	RGI60-05.00623	G306428E69317N	3735	0.000866333
**GL39_DN_2**	Southwest Greenland	30.07.19	15:19	2.3	Chamberlin	69.321	-53.4954	332	1306	4.77	0.47	10.21	RGI60-05.00623	G306428E69317N	3735	0.000372333
**GL39_DN_3**	Southwest Greenland	30.07.19	15:19	2.3	Chamberlin	69.321	-53.4954	332	1306	4.77	0.47	10.21	RGI60-05.00623	G306428E69317N	3735	0.000311333
**GL40_UP_1**	Southwest Greenland	31.07.19	12:25	2.8	Petersen	69.3013	-53.5476	576	66	0.66	0.94	0.7	RGI60-05.00623	G306433E69302N	3741	0.00434
**GL40_UP_2**	Southwest Greenland	31.07.19	12:25	2.8	Petersen	69.3013	-53.5476	576	66	0.66	0.94	0.7	RGI60-05.00623	G306433E69302N	3741	0.004811
**GL40_UP_3**	Southwest Greenland	31.07.19	12:25	2.8	Petersen	69.3013	-53.5476	576	66	0.66	0.94	0.7	RGI60-05.00623	G306433E69302N	3741	0.003034667
**GL40_DN_1**	Southwest Greenland	31.07.19	15:20	5.3	Petersen	69.2999	-53.5273	446	877	0.67	0.54	1.26	RGI60-05.00623	G306433E69302N	3741	0.005824
**GL40_DN_2**	Southwest Greenland	31.07.19	15:20	5.3	Petersen	69.2999	-53.5273	446	877	0.67	0.54	1.26	RGI60-05.00623	G306433E69302N	3741	0.002817667
**GL40_DN_3**	Southwest Greenland	31.07.19	15:20	5.3	Petersen	69.2999	-53.5273	446	877	0.67	0.54	1.26	RGI60-05.00623	G306433E69302N	3741	0.003789333
**GL41_UP_1**	Southwest Greenland	01.08.19	11:32	0.5	Pjetursson	69.2955	-53.3929	629	25	0.45	0.66	0.67	RGI60-05.00602	G306613E69293N	3742	0.001634667
**GL41_UP_2**	Southwest Greenland	01.08.19	11:32	0.5	Pjetursson	69.2955	-53.3929	629	25	0.45	0.66	0.67	RGI60-05.00602	G306613E69293N	3742	0.001282667
**GL41_UP_3**	Southwest Greenland	01.08.19	11:32	0.5	Pjetursson	69.2955	-53.3929	629	25	0.45	0.66	0.67	RGI60-05.00602	G306613E69293N	3742	0.001794333
**GL41_DN_1**	Southwest Greenland	01.08.19	16:35	4.5	Pjetursson	69.3009	-53.4289	395	1567	0.47	0.12	3.75	RGI60-05.00602	G306613E69293N	3742	0.019440667
**GL41_DN_2**	Southwest Greenland	01.08.19	16:35	4.5	Pjetursson	69.3009	-53.4289	395	1567	0.47	0.12	3.75	RGI60-05.00602	G306613E69293N	3742	0.006369667
**GL41_DN_3**	Southwest Greenland	01.08.19	16:35	4.5	Pjetursson	69.3009	-53.4289	395	1567	0.47	0.12	3.75	RGI60-05.00602	G306613E69293N	3742	0.005484333
**GL42_UP_1**	Caucasus Mountains	11.09.19	12:09	0.3	Midjirgi	43.0936	43.1692	2637	122	14.92	0.48	31.23	RGI60-12.01262	G043172E43060N	1509	0.003801

**GL42_UP_2**	Caucasus Mountains	11.09.19	12:09	0.3	Midjirgi	43.0936	43.1692	2637	122	14.92	0.48	31.23	RGI60-12.01262	G043172E43060N	1509	0.001527667
**GL42_UP_3**	Caucasus Mountains	11.09.19	12:09	0.3	Midjirgi	43.0936	43.1692	2637	122	14.92	0.48	31.23	RGI60-12.01262	G043172E43060N	1509	0.001299
**GL42_DN_1**	Caucasus Mountains	08.09.19	11:35	3.8	Midjirgi	43.1122	43.1494	2121	2737	16.41	0.36	45.68	RGI60-12.01262	G043172E43060N	1509	0.009174
**GL42_DN_2**	Caucasus Mountains	08.09.19	11:35	3.8	Midjirgi	43.1122	43.1494	2121	2737	16.41	0.36	45.68	RGI60-12.01262	G043172E43060N	1509	0.004156
**GL42_DN_3**	Caucasus Mountains	08.09.19	11:35	3.8	Midjirgi	43.1122	43.1494	2121	2737	16.41	0.36	45.68	RGI60-12.01262	G043172E43060N	1509	0.009060333
**GL43_UP_1**	Caucasus Mountains	09.09.19	10:05	0	Bezengi	43.1057	43.1318	2190	81	42.12	0.45	93.79	RGI50-12.00014	G043084E43033N	703	0.001277
**GL43_UP_2**	Caucasus Mountains	09.09.19	10:05	0	Bezengi	43.1057	43.1318	2190	81	42.12	0.45	93.79	RGI50-12.00014	G043084E43033N	703	0.001102667
**GL43_UP_3**	Caucasus Mountains	09.09.19	10:05	0	Bezengi	43.1057	43.1318	2190	81	42.12	0.45	93.79	RGI50-12.00014	G043084E43033N	703	0
**GL43_DN_1**	Caucasus Mountains	08.09.19	04:48	0.6	Bezengi	43.1172	43.1493	2037	1993	42.13	0.42	100.78	RGI50-12.00014	G043084E43033N	703	0.001734667
**GL43_DN_2**	Caucasus Mountains	08.09.19	04:48	0.6	Bezengi	43.1172	43.1493	2037	1993	42.13	0.42	100.78	RGI50-12.00014	G043084E43033N	703	0.000341667
**GL43_DN_3**	Caucasus Mountains	08.09.19	04:48	0.6	Bezengi	43.1172	43.1493	2037	1993	42.13	0.42	100.78	RGI50-12.00014	G043084E43033N	703	0
**GL44_UP_1**	Caucasus Mountains	10.09.19	13:00	0.2	"50"/"442"	43.0924	43.199	3481	197	0.96	0.42	2.26	RGI60-12.00595	G043218E43085N	NA	0.008527
**GL44_UP_2**	Caucasus Mountains	10.09.19	13:00	0.2	"50"/"442"	43.0924	43.199	3481	197	0.96	0.42	2.26	RGI60-12.00595	G043218E43085N	NA	0.003952
**GL44_UP_3**	Caucasus Mountains	10.09.19	13:00	0.2	"50"/"442"	43.0924	43.199	3481	197	0.96	0.42	2.26	RGI60-12.00595	G043218E43085N	NA	0.005223667
**GL44_DN_1**	Caucasus Mountains	11.09.19	15:33	3	"50"/"442"	43.0941	43.1733	2660	2297	1.06	0.2	5.25	RGI60-12.00595	G043218E43085N	NA	0.510728667
**GL44_DN_2**	Caucasus Mountains	11.09.19	15:33	3	"50"/"442"	43.0941	43.1733	2660	2297	1.06	0.2	5.25	RGI60-12.00595	G043218E43085N	NA	0.169341667
**GL44_DN_3**	Caucasus Mountains	11.09.19	15:33	3	"50"/"442"	43.0941	43.1733	2660	2297	1.06	0.2	5.25	RGI60-12.00595	G043218E43085N	NA	0.210178667
**GL45_UP_1**	Caucasus Mountains	12.09.19	12:02	0.6	"50"/"4446"	43.0752	43.1743	3027	415	0.31	0.26	1.22	RGI60-12.01187	G043188E43071N	NA	0.001939667
**GL45_UP_2**	Caucasus Mountains	12.09.19	12:02	0.6	"50"/"4446"	43.0752	43.1743	3027	415	0.31	0.26	1.22	RGI60-12.01187	G043188E43071N	NA	0.002615

**GL45_UP_3**	Caucasus Mountains	12.09.19	12:02	0.6	"50"/"4446"	43.0752	43.1743	3027	415	0.31	0.26	1.22	RGI60-12.01187	G043188E43071N	NA	0.026807
**GL45_DN_1**	Caucasus Mountains	12.09.19	14:51	4.7	"50"/"4446"	43.0751	43.1714	2924	645	0.31	0.25	1.25	RGI60-12.01187	G043188E43071N	NA	0.075332
**GL45_DN_2**	Caucasus Mountains	12.09.19	14:51	4.7	"50"/"4446"	43.0751	43.1714	2924	645	0.31	0.25	1.25	RGI60-12.01187	G043188E43071N	NA	0.058852333
**GL45_DN_3**	Caucasus Mountains	12.09.19	14:51	4.7	"50"/"4446"	43.0751	43.1714	2924	645	0.31	0.25	1.25	RGI60-12.01187	G043188E43071N	NA	0.044089
**GL46_UP_1**	Caucasus Mountains	13.09.19	11:30	0.4	"443"	43.0832	43.1839	3154	440	1.57	0.55	2.88	RGI60-12.00396	G043201E43079N	NA	0.048202667
**GL46_UP_2**	Caucasus Mountains	13.09.19	11:30	0.4	"443"	43.0832	43.1839	3154	440	1.57	0.55	2.88	RGI60-12.00396	G043201E43079N	NA	0.036712333
**GL46_UP_3**	Caucasus Mountains	13.09.19	11:30	0.4	"443"	43.0832	43.1839	3154	440	1.57	0.55	2.88	RGI60-12.00396	G043201E43079N	NA	0.128029
**GL46_DN_1**	Caucasus Mountains	13.09.19	14:30	7	"443"	43.0858	43.173	2779	1373	1.58	0.4	3.92	RGI60-12.00396	G043201E43079N	NA	0.044039
**GL46_DN_2**	Caucasus Mountains	13.09.19	14:30	7	"443"	43.0858	43.173	2779	1373	1.58	0.4	3.92	RGI60-12.00396	G043201E43079N	NA	0.115836
**GL46_DN_3**	Caucasus Mountains	13.09.19	14:30	7	"443"	43.0858	43.173	2779	1373	1.58	0.4	3.92	RGI60-12.00396	G043201E43079N	NA	0.016782
**GL47_UP_1**	Caucasus Mountains	17.09.19	10:23	0.5	Kashkatash	43.2116	42.6847	2517	122	3.04	0.42	7.22	RGI60-12.00259	G042693E43189N	4318	0.015034333
**GL47_UP_2**	Caucasus Mountains	17.09.19	10:23	0.5	Kashkatash	43.2116	42.6847	2517	122	3.04	0.42	7.22	RGI60-12.00259	G042693E43189N	4318	0.025104667
**GL47_UP_3**	Caucasus Mountains	17.09.19	10:23	0.5	Kashkatash	43.2116	42.6847	2517	122	3.04	0.42	7.22	RGI60-12.00259	G042693E43189N	4318	0.010400667
**GL47_DN_1**	Caucasus Mountains	17.09.19	14:31	2.4	Kashkatash	43.2203	42.6839	2177	1087	3.04	0.39	7.75	RGI60-12.00259	G042693E43189N	4318	0.11745
**GL47_DN_2**	Caucasus Mountains	17.09.19	14:31	2.4	Kashkatash	43.2203	42.6839	2177	1087	3.04	0.39	7.75	RGI60-12.00259	G042693E43189N	4318	0.041785333
**GL47_DN_3**	Caucasus Mountains	17.09.19	14:31	2.4	Kashkatash	43.2203	42.6839	2177	1087	3.04	0.39	7.75	RGI60-12.00259	G042693E43189N	4318	0.040395667
**GL48_UP_1**	Caucasus Mountains	18.09.19	10:59	1	Djankuat	43.2034	42.7501	2760	29	2.44	0.67	3.61	RGI50-12.01132	G042766E43192N	726	0.006356667
**GL48_UP_2**	Caucasus Mountains	18.09.19	10:59	1	Djankuat	43.2034	42.7501	2760	29	2.44	0.67	3.61	RGI50-12.01132	G042766E43192N	726	0.038353
**GL48_UP_3**	Caucasus Mountains	18.09.19	10:59	1	Djankuat	43.2034	42.7501	2760	29	2.44	0.67	3.61	RGI50-12.01132	G042766E43192N	726	0.011656667

**GL48_DN_1**	Caucasus Mountains	18.09.19	14:36	3.9	Djankuat	43.2088	42.7396	2642	1075	2.73	0.33	8.4	RGI50-12.01132	G042766E43192N	726	0.009819
**GL48_DN_2**	Caucasus Mountains	18.09.19	14:36	3.9	Djankuat	43.2088	42.7396	2642	1075	2.73	0.33	8.4	RGI50-12.01132	G042766E43192N	726	0.007130667
**GL48_DN_3**	Caucasus Mountains	18.09.19	14:36	3.9	Djankuat	43.2088	42.7396	2642	1075	2.73	0.33	8.4	RGI50-12.01132	G042766E43192N	726	0.007120667
**GL49_UP_1**	Caucasus Mountains	19.09.19	11:38	1.9	Djantugan	43.198	42.7382	2799	63	0.5	0.64	0.78	RGI60-12.00314	G042746E43195N	NA	0.017017
**GL49_UP_2**	Caucasus Mountains	19.09.19	11:38	1.9	Djantugan	43.198	42.7382	2799	63	0.5	0.64	0.78	RGI60-12.00314	G042746E43195N	NA	0.023936667
**GL49_UP_3**	Caucasus Mountains	19.09.19	11:38	1.9	Djantugan	43.198	42.7382	2799	63	0.5	0.64	0.78	RGI60-12.00314	G042746E43195N	NA	0.020288667
**GL49_DN_1**	Caucasus Mountains	19.09.19	14:16	4.1	Djantugan	43.1992	42.7342	2741	413	0.56	0.47	1.18	RGI60-12.00314	G042746E43195N	NA	0.027558667
**GL49_DN_2**	Caucasus Mountains	19.09.19	14:16	4.1	Djantugan	43.1992	42.7342	2741	413	0.56	0.47	1.18	RGI60-12.00314	G042746E43195N	NA	0.036984667
**GL49_DN_3**	Caucasus Mountains	19.09.19	14:16	4.1	Djantugan	43.1992	42.7342	2741	413	0.56	0.47	1.18	RGI60-12.00314	G042746E43195N	NA	0.014621667
**GL50_UP_1**	Caucasus Mountains	20.09.19	12:03	0.8	Shkhelda	43.1952	42.6484	2364	84	11.27	0.48	23.69	RGI60-12.00426	G042639E43169N	NA	0.009853
**GL50_UP_2**	Caucasus Mountains	20.09.19	12:03	0.8	Shkhelda	43.1952	42.6484	2364	84	11.27	0.48	23.69	RGI60-12.00426	G042639E43169N	NA	0.002656667
**GL50_UP_3**	Caucasus Mountains	20.09.19	12:03	0.8	Shkhelda	43.1952	42.6484	2364	84	11.27	0.48	23.69	RGI60-12.00426	G042639E43169N	NA	0.009706333
**GL50_DN_1**	Caucasus Mountains	20.09.19	15:20	2	Shkhelda	43.208	42.6511	2212	1525	13.72	0.39	35.02	RGI60-12.00426	G042639E43169N	NA	0.040711
**GL50_DN_2**	Caucasus Mountains	20.09.19	15:20	2	Shkhelda	43.208	42.6511	2212	1525	13.72	0.39	35.02	RGI60-12.00426	G042639E43169N	NA	0.027668333
**GL50_DN_3**	Caucasus Mountains	20.09.19	15:20	2	Shkhelda	43.208	42.6511	2212	1525	13.72	0.39	35.02	RGI60-12.00426	G042639E43169N	NA	0.078774667
**GL51_UP_1**	Caucasus Mountains	22.09.19	10:13	1	Bashkara	43.2099	42.7242	2576	47	4.42	0.52	8.49	RGI60-12.00849	G042727E43193N	NA	0.116456
**GL51_UP_2**	Caucasus Mountains	22.09.19	10:13	1	Bashkara	43.2099	42.7242	2576	47	4.42	0.52	8.49	RGI60-12.00849	G042727E43193N	NA	0.116893
**GL51_UP_3**	Caucasus Mountains	22.09.19	10:13	1	Bashkara	43.2099	42.7242	2576	47	4.42	0.52	8.49	RGI60-12.00849	G042727E43193N	NA	0.068305
**GL51_DN_1**	Caucasus Mountains	22.09.19	13:30	2.5	Bashkara	43.2133	42.7148	2466	898	4.47	0.47	9.54	RGI60-12.00849	G042727E43193N	NA	0.506979

**GL51_DN_2**	Caucasus Mountains	22.09.19	13:30	2.5	Bashkara	43.2133	42.7148	2466	898	4.47	0.47	9.54	RGI60-12.00849	G042727E43193N	NA	0.218531667
**GL51_DN_3**	Caucasus Mountains	22.09.19	13:30	2.5	Bashkara	43.2133	42.7148	2466	898	4.47	0.47	9.54	RGI60-12.00849	G042727E43193N	NA	0.330091
**GL52_UP_1**	Caucasus Mountains	23.09.19	11:33	5	Terskol	43.291	42.5067	2709	1108	7.34	0.61	11.99	RGI60-12.00821	G042482E43317N	720	0.006012
**GL52_UP_2**	Caucasus Mountains	23.09.19	11:33	5	Terskol	43.291	42.5067	2709	1108	7.34	0.61	11.99	RGI60-12.00821	G042482E43317N	720	0.013647667
**GL52_UP_3**	Caucasus Mountains	23.09.19	11:33	5	Terskol	43.291	42.5067	2709	1108	7.34	0.61	11.99	RGI60-12.00821	G042482E43317N	720	0.012305333
**GL52_DN_1**	Caucasus Mountains	23.09.19	14:23	5.7	Terskol	43.2753	42.5128	2387	2927	7.36	0.4	18.39	RGI60-12.00821	G042482E43317N	720	0.007409
**GL52_DN_2**	Caucasus Mountains	23.09.19	14:23	5.7	Terskol	43.2753	42.5128	2387	2927	7.36	0.4	18.39	RGI60-12.00821	G042482E43317N	720	0.015689
**GL52_DN_3**	Caucasus Mountains	23.09.19	14:23	5.7	Terskol	43.2753	42.5128	2387	2927	7.36	0.4	18.39	RGI60-12.00821	G042482E43317N	720	0.016240667
**GL53_UP_1**	Caucasus Mountains	26.09.19	13:30	7.3	Garabashi	43.2846	42.4788	3059	953	4.11	0.69	5.93	RGI50-12.00161	G042467E43312N	761	0.015046667
**GL53_UP_2**	Caucasus Mountains	26.09.19	13:30	7.3	Garabashi	43.2846	42.4788	3059	953	4.11	0.69	5.93	RGI50-12.00161	G042467E43312N	761	0.032398
**GL53_UP_3**	Caucasus Mountains	26.09.19	13:30	7.3	Garabashi	43.2846	42.4788	3059	953	4.11	0.69	5.93	RGI50-12.00161	G042467E43312N	761	0.03111
**GL53_DN_1**	Caucasus Mountains	24.09.19	15:57	6.3	Garabashi	43.2677	42.4881	2400	2983	4.14	0.42	9.93	RGI50-12.00161	G042467E43312N	761	0.100529
**GL53_DN_2**	Caucasus Mountains	24.09.19	15:57	6.3	Garabashi	43.2677	42.4881	2400	2983	4.14	0.42	9.93	RGI50-12.00161	G042467E43312N	761	0.011651333
**GL53_DN_3**	Caucasus Mountains	24.09.19	15:57	6.3	Garabashi	43.2677	42.4881	2400	2983	4.14	0.42	9.93	RGI50-12.00161	G042467E43312N	761	0.024013
**GL54_UP_1**	Caucasus Mountains	25.09.19	12:19	2.4	Bolshoy Azau	43.2721	42.4468	2561	927	21.8	0.77	28.14	RGI50-12.00080	G042422E43310N	701	0.003411
**GL54_UP_2**	Caucasus Mountains	25.09.19	12:19	2.4	Bolshoy Azau	43.2721	42.4468	2561	927	21.8	0.77	28.14	RGI50-12.00080	G042422E43310N	701	0.002961
**GL54_UP_3**	Caucasus Mountains	25.09.19	12:19	2.4	Bolshoy Azau	43.2721	42.4468	2561	927	21.8	0.77	28.14	RGI50-12.00080	G042422E43310N	701	0.004543333
**GL54_DN_1**	Caucasus Mountains	25.09.19	15:40	4.1	Bolshoy Azau	43.2654	42.4668	2394	2712	26.17	0.62	42.43	RGI50-12.00080	G042422E43310N	701	0.003893
**GL54_DN_2**	Caucasus Mountains	25.09.19	15:40	4.1	Bolshoy Azau	43.2654	42.4668	2394	2712	26.17	0.62	42.43	RGI50-12.00080	G042422E43310N	701	0.003521

**GL54_DN_3**	Caucasus Mountains	25.09.19	15:40	4.1	Bolshoy Azau	43.2654	42.4668	2394	2712	26.17	0.62	42.43	RGI50-12.00080	G042422E43310N	701	0.007455333
**GL55_UP_1**	Caucasus Mountains	29.09.19	12:01	2.3	Irik	43.3029	42.5498	2661	301	9.33	0.49	19.01	RGI50-12.00730	G042500E43332N	759	0.004309
**GL55_UP_2**	Caucasus Mountains	29.09.19	12:01	2.3	Irik	43.3029	42.5498	2661	301	9.33	0.49	19.01	RGI50-12.00730	G042500E43332N	759	0.003248
**GL55_UP_3**	Caucasus Mountains	29.09.19	12:01	2.3	Irik	43.3029	42.5498	2661	301	9.33	0.49	19.01	RGI50-12.00730	G042500E43332N	759	0.015098667
**GL55_DN_1**	Caucasus Mountains	29.09.19	15:37	6	Irik	43.292	42.5921	2361	3939	10.67	0.21	51.83	RGI50-12.00730	G042500E43332N	759	0.003822667
**GL55_DN_2**	Caucasus Mountains	29.09.19	15:37	6	Irik	43.292	42.5921	2361	3939	10.67	0.21	51.83	RGI50-12.00730	G042500E43332N	759	0.010331333
**GL55_DN_3**	Caucasus Mountains	29.09.19	15:37	6	Irik	43.292	42.5921	2361	3939	10.67	0.21	51.83	RGI50-12.00730	G042500E43332N	759	0.004982667
**GL56_UP_1**	Caucasus Mountains	02.10.19	10:42	4.7	Sopruju Sevenriy	43.2617	41.6102	2104	1028	2.94	0.65	4.56	RGI60-12.00474	G041589E43251N	NA	0.028401333
**GL56_UP_2**	Caucasus Mountains	02.10.19	10:42	4.7	Sopruju Sevenriy	43.2617	41.6102	2104	1028	2.94	0.65	4.56	RGI60-12.00474	G041589E43251N	NA	0.024273
**GL56_UP_3**	Caucasus Mountains	02.10.19	10:42	4.7	Sopruju Sevenriy	43.2617	41.6102	2104	1028	2.94	0.65	4.56	RGI60-12.00474	G041589E43251N	NA	0.017891667
**GL56_DN_1**	Caucasus Mountains	02.10.19	14:18	7.3	Sopruju Sevenriy	43.2683	41.6149	1848	1847	3.11	0.42	7.33	RGI60-12.00474	G041589E43251N	NA	0.047384333
**GL56_DN_2**	Caucasus Mountains	02.10.19	14:18	7.3	Sopruju Sevenriy	43.2683	41.6149	1848	1847	3.11	0.42	7.33	RGI60-12.00474	G041589E43251N	NA	0.051797333
**GL56_DN_3**	Caucasus Mountains	02.10.19	14:18	7.3	Sopruju Sevenriy	43.2683	41.6149	1848	1847	3.11	0.42	7.33	RGI60-12.00474	G041589E43251N	NA	0.032407667
**GL57_UP_1**	Caucasus Mountains	03.10.19	10:30	3.7	Amanauz	43.2622	41.6141	1966	785	7.17	0.51	14.05	RGI60-12.01112	G041628E43233N	NA	0.002440667
**GL57_UP_2**	Caucasus Mountains	03.10.19	10:30	3.7	Amanauz	43.2622	41.6141	1966	785	7.17	0.51	14.05	RGI60-12.01112	G041628E43233N	NA	0.000515333
**GL57_UP_3**	Caucasus Mountains	03.10.19	10:30	3.7	Amanauz	43.2622	41.6141	1966	785	7.17	0.51	14.05	RGI60-12.01112	G041628E43233N	NA	0.000662667
**GL57_DN_1**	Caucasus Mountains	03.10.19	14:27	4.6	Amanauz	43.2685	41.6151	1848	1489	10.36	0.43	24.3	RGI60-12.01112	G041628E43233N	NA	0.001191
**GL57_DN_2**	Caucasus Mountains	03.10.19	14:27	4.6	Amanauz	43.2685	41.6151	1848	1489	10.36	0.43	24.3	RGI60-12.01112	G041628E43233N	NA	0.001075667
**GL57_DN_3**	Caucasus Mountains	03.10.19	14:27	4.6	Amanauz	43.2685	41.6151	1848	1489	10.36	0.43	24.3	RGI60-12.01112	G041628E43233N	NA	0.001462

**GL58_UP_1**	Caucasus Mountains	04.10.19	10:30	0.6	Ptish	43.2356	41.6929	2347	87	1.35	0.58	2.33	RGI60-12.00063	G041694E43227N	NA	0.000681033
**GL58_UP_2**	Caucasus Mountains	04.10.19	10:30	0.6	Ptish	43.2356	41.6929	2347	87	1.35	0.58	2.33	RGI60-12.00063	G041694E43227N	NA	7.09E-05
**GL58_UP_3**	Caucasus Mountains	04.10.19	10:30	0.6	Ptish	43.2356	41.6929	2347	87	1.35	0.58	2.33	RGI60-12.00063	G041694E43227N	NA	2.6E-05
**GL58_DN_1**	Caucasus Mountains	04.10.19	13:54	5.4	Ptish	43.2512	41.6916	2113	1825	4.12	0.3	13.9	RGI60-12.00063	G041694E43227N	NA	0.00325
**GL58_DN_2**	Caucasus Mountains	04.10.19	13:54	5.4	Ptish	43.2512	41.6916	2113	1825	4.12	0.3	13.9	RGI60-12.00063	G041694E43227N	NA	0.006871333
**GL58_DN_3**	Caucasus Mountains	04.10.19	13:54	5.4	Ptish	43.2512	41.6916	2113	1825	4.12	0.3	13.9	RGI60-12.00063	G041694E43227N	NA	0.004995333
**GL59_UP_1**	Caucasus Mountains	05.10.19	12:36	3.5	"789"	43.2668	41.6772	1994	831	0.54	0.3	1.79	RGI60-12.00053	G041670E43254N	NA	0.003599333
**GL59_UP_2**	Caucasus Mountains	05.10.19	12:36	3.5	"789"	43.2668	41.6772	1994	831	0.54	0.3	1.79	RGI60-12.00053	G041670E43254N	NA	0.008434667
**GL59_UP_3**	Caucasus Mountains	05.10.19	12:36	3.5	"789"	43.2668	41.6772	1994	831	0.54	0.3	1.79	RGI60-12.00053	G041670E43254N	NA	0.001406667
**GL59_DN_1**	Caucasus Mountains	05.10.19	15:19	3.7	"789"	43.2682	41.6782	1927	1006	0.54	0.3	1.81	RGI60-12.00053	G041670E43254N	NA	0.001708667
**GL59_DN_2**	Caucasus Mountains	05.10.19	15:19	3.7	"789"	43.2682	41.6782	1927	1006	0.54	0.3	1.81	RGI60-12.00053	G041670E43254N	NA	0.002143333
**GL59_DN_3**	Caucasus Mountains	05.10.19	15:19	3.7	"789"	43.2682	41.6782	1927	1006	0.54	0.3	1.81	RGI60-12.00053	G041670E43254N	NA	0.001192667
**GL60_UP_1**	Caucasus Mountains	08.10.19	10:40	3.9	Dvuyazichniy	43.2983	41.5478	2109	1507	2.06	0.44	4.64	RGI60-12.00842	G041519E43297N	NA	0.000625
**GL60_UP_2**	Caucasus Mountains	08.10.19	10:40	3.9	Dvuyazichniy	43.2983	41.5478	2109	1507	2.06	0.44	4.64	RGI60-12.00842	G041519E43297N	NA	0.001834333
**GL60_UP_3**	Caucasus Mountains	08.10.19	10:40	3.9	Dvuyazichniy	43.2983	41.5478	2109	1507	2.06	0.44	4.64	RGI60-12.00842	G041519E43297N	NA	0.003705333
**GL60_DN_1**	Caucasus Mountains	08.10.19	14:23	5.1	Dvuyazichniy	43.2989	41.5569	1920	2244	2.06	0.43	4.84	RGI60-12.00842	G041519E43297N	NA	0.002003333
**GL60_DN_2**	Caucasus Mountains	08.10.19	14:23	5.1	Dvuyazichniy	43.2989	41.5569	1920	2244	2.06	0.43	4.84	RGI60-12.00842	G041519E43297N	NA	0.001317
**GL60_DN_3**	Caucasus Mountains	08.10.19	14:23	5.1	Dvuyazichniy	43.2989	41.5569	1920	2244	2.06	0.43	4.84	RGI60-12.00842	G041519E43297N	NA	0.00278
**GL61_UP_1**	Ecuadorian Andes	08.02.20	11:20	3.8	"San Marco's"	0.0402	-77.9969	4698	223	0.74	0.73	1.01	RGI60-16.01343	G282003E00032N	NA	0.001404667

**GL61_UP_2**	Ecuadorian Andes	08.02.20	11:20	3.8	"San Marco's"	0.0402	-77.9969	4698	223	0.74	0.73	1.01	RGI60-16.01343	G282003E00032N	NA	0.002374333
**GL61_UP_3**	Ecuadorian Andes	08.02.20	11:20	3.8	"San Marco's"	0.0402	-77.9969	4698	223	0.74	0.73	1.01	RGI60-16.01343	G282003E00032N	NA	0.002784
**GL61_DN_1**	Ecuadorian Andes	08.02.20	14:52	3.1	"San Marco's"	0.043	-77.9998	4620	675	0.74	0.59	1.25	RGI60-16.01343	G282003E00032N	NA	0.004181
**GL61_DN_2**	Ecuadorian Andes	08.02.20	14:52	3.1	"San Marco's"	0.043	-77.9998	4620	675	0.74	0.59	1.25	RGI60-16.01343	G282003E00032N	NA	0.031464333
**GL61_DN_3**	Ecuadorian Andes	08.02.20	14:52	3.1	"San Marco's"	0.043	-77.9998	4620	675	0.74	0.59	1.25	RGI60-16.01343	G282003E00032N	NA	0.023910667
**GL62_UP_1**	Ecuadorian Andes	09.02.20	11:21	0.9	"Laguna Verde West"	0.0167	-78.0065	4861	31	0.75	0.85	0.88	RGI60-16.01345	G282007E00013N	NA	0.002679
**GL62_UP_2**	Ecuadorian Andes	09.02.20	11:21	0.9	"Laguna Verde West"	0.0167	-78.0065	4861	31	0.75	0.85	0.88	RGI60-16.01345	G282007E00013N	NA	0.000598
**GL62_UP_3**	Ecuadorian Andes	09.02.20	11:21	0.9	"Laguna Verde West"	0.0167	-78.0065	4861	31	0.75	0.85	0.88	RGI60-16.01345	G282007E00013N	NA	0.000417333
**GL62_DN_1**	Ecuadorian Andes	09.02.20	14:07	1.4	"Laguna Verde West"	0.0157	-78.0079	4780	221	0.75	0.8	0.95	RGI60-16.01345	G282007E00013N	NA	0.001253667
**GL62_DN_2**	Ecuadorian Andes	09.02.20	14:07	1.4	"Laguna Verde West"	0.0157	-78.0079	4780	221	0.75	0.8	0.95	RGI60-16.01345	G282007E00013N	NA	0.000647
**GL62_DN_3**	Ecuadorian Andes	09.02.20	14:07	1.4	"Laguna Verde West"	0.0157	-78.0079	4780	221	0.75	0.8	0.95	RGI60-16.01345	G282007E00013N	NA	0.001658
**GL63_UP_1**	Ecuadorian Andes	10.02.20	12:13	1.4	Hermoso	0.0107	-78.0068	4661	45	0.8	0.96	0.83	RGI60-16.01345	G282007E00013N	NA	0.000932667
**GL63_UP_2**	Ecuadorian Andes	10.02.20	12:13	1.4	Hermoso	0.0107	-78.0068	4661	45	0.8	0.96	0.83	RGI60-16.01345	G282007E00013N	NA	0.000636
**GL63_UP_3**	Ecuadorian Andes	10.02.20	12:13	1.4	Hermoso	0.0107	-78.0068	4661	45	0.8	0.96	0.83	RGI60-16.01345	G282007E00013N	NA	0.000401667
**GL63_DN_1**	Ecuadorian Andes	10.02.20	15:17	0.7	Hermoso	0.0104	-78.0068	4647	83	0.8	0.96	0.83	RGI60-16.01345	G282007E00013N	NA	0.000299
**GL63_DN_2**	Ecuadorian Andes	10.02.20	15:17	0.7	Hermoso	0.0104	-78.0068	4647	83	0.8	0.96	0.83	RGI60-16.01345	G282007E00013N	NA	0.002395
**GL63_DN_3**	Ecuadorian Andes	10.02.20	15:17	0.7	Hermoso	0.0104	-78.0068	4647	83	0.8	0.96	0.83	RGI60-16.01345	G282007E00013N	NA	0.002051333
**GL64_UP_1**	Ecuadorian Andes	11.02.20	11:28	0.5	"Laguna Verde East"	0.0161	-78.0055	4894	37	0.75	0.86	0.88	RGI60-16.01345	G282007E00013N	NA	0.002033333
**GL64_UP_2**	Ecuadorian Andes	11.02.20	11:28	0.5	"Laguna Verde East"	0.0161	-78.0055	4894	37	0.75	0.86	0.88	RGI60-16.01345	G282007E00013N	NA	0.004424

**GL64_UP_3**	Ecuadorian Andes	11.02.20	11:28	0.5	"Laguna Verde East"	0.0161	-78.0055	4894	37	0.75	0.86	0.88	RGI60-16.01345	G282007E00013N	NA	0.004349333
**GL64_DN_1**	Ecuadorian Andes	11.02.20	14:44	5.7	"Laguna Verde East"	0.0148	-78.0072	4801	269	0.75	0.79	0.95	RGI60-16.01345	G282007E00013N	NA	0.009882667
**GL64_DN_2**	Ecuadorian Andes	11.02.20	14:44	5.7	"Laguna Verde East"	0.0148	-78.0072	4801	269	0.75	0.79	0.95	RGI60-16.01345	G282007E00013N	NA	0.007440333
**GL64_DN_3**	Ecuadorian Andes	11.02.20	14:44	5.7	"Laguna Verde East"	0.0148	-78.0072	4801	269	0.75	0.79	0.95	RGI60-16.01345	G282007E00013N	NA	0.009888
**GL65_UP_1**	Ecuadorian Andes	13.02.20	12:00	0.4	Antisana 12/ Los Crespos	-0.4941	-78.1587	4746	48	1.34	0.96	1.4	RGI60-16.01339	G281855E00490S	NA	0.000964
**GL65_UP_2**	Ecuadorian Andes	13.02.20	12:00	0.4	Antisana 12/ Los Crespos	-0.4941	-78.1587	4746	48	1.34	0.96	1.4	RGI60-16.01339	G281855E00490S	NA	0.001482333
**GL65_UP_3**	Ecuadorian Andes	13.02.20	12:00	0.4	Antisana 12/ Los Crespos	-0.4941	-78.1587	4746	48	1.34	0.96	1.4	RGI60-16.01339	G281855E00490S	NA	0.003670667
**GL65_DN_1**	Ecuadorian Andes	13.02.20	14:30	0.9	Antisana 12/ Los Crespos	-0.4942	-78.1588	4739	70	1.34	0.96	1.41	RGI60-16.01339	G281855E00490S	NA	0.007244667
**GL65_DN_2**	Ecuadorian Andes	13.02.20	14:30	0.9	Antisana 12/ Los Crespos	-0.4942	-78.1588	4739	70	1.34	0.96	1.41	RGI60-16.01339	G281855E00490S	NA	0.008705
**GL65_DN_3**	Ecuadorian Andes	13.02.20	14:30	0.9	Antisana 12/ Los Crespos	-0.4942	-78.1588	4739	70	1.34	0.96	1.41	RGI60-16.01339	G281855E00490S	NA	0.004693
**GL66_UP_1**	Ecuadorian Andes	14.02.20	12:05	4.5	Antisana 15 beta	-0.4724	-78.1511	4872	92	0.28	0.94	0.29	RGI60-16.01339	G281855E00490S	1624	0.003842667
**GL66_UP_2**	Ecuadorian Andes	14.02.20	12:05	4.5	Antisana 15 beta	-0.4724	-78.1511	4872	92	0.28	0.94	0.29	RGI60-16.01339	G281855E00490S	1624	0.003515333
**GL66_UP_3**	Ecuadorian Andes	14.02.20	12:05	4.5	Antisana 15 beta	-0.4724	-78.1511	4872	92	0.28	0.94	0.29	RGI60-16.01339	G281855E00490S	1624	0.002125333
**GL66_DN_1**	Ecuadorian Andes	14.02.20	15:05	5.4	Antisana 15 beta	-0.4716	-78.1549	4783	526	0.28	0.62	0.45	RGI60-16.01339	G281855E00490S	1624	0.01021
**GL66_DN_2**	Ecuadorian Andes	14.02.20	15:05	5.4	Antisana 15 beta	-0.4716	-78.1549	4783	526	0.28	0.62	0.45	RGI60-16.01339	G281855E00490S	1624	0.008704333
**GL66_DN_3**	Ecuadorian Andes	14.02.20	15:05	5.4	Antisana 15 beta	-0.4716	-78.1549	4783	526	0.28	0.62	0.45	RGI60-16.01339	G281855E00490S	1624	0.005958
**GL67_UP_1**	Ecuadorian Andes	15.02.20	11:44	5.5	Antisana 15 alpha	-0.4739	-78.1543	4832	77	0.28	0.81	0.35	RGI60-16.01339	G281855E00490S	1624	0.000462667
**GL67_UP_2**	Ecuadorian Andes	15.02.20	11:44	5.5	Antisana 15 alpha	-0.4739	-78.1543	4832	77	0.28	0.81	0.35	RGI60-16.01339	G281855E00490S	1624	9.97E-06
**GL67_UP_3**	Ecuadorian Andes	15.02.20	11:44	5.5	Antisana 15 alpha	-0.4739	-78.1543	4832	77	0.28	0.81	0.35	RGI60-16.01339	G281855E00490S	1624	0.000292667

**GL67_DN_1**	Ecuadorian Andes	15.02.20	14:15	5	Antisana 15 alpha	-0.4718	-78.1555	4782	344	0.28	0.69	0.41	RGI60-16.01339	G281855E00490S	1624	0.000913667
**GL67_DN_2**	Ecuadorian Andes	15.02.20	14:15	5	Antisana 15 alpha	-0.4718	-78.1555	4782	344	0.28	0.69	0.41	RGI60-16.01339	G281855E00490S	1624	0.001760667
**GL67_DN_3**	Ecuadorian Andes	15.02.20	14:15	5	Antisana 15 alpha	-0.4718	-78.1555	4782	344	0.28	0.69	0.41	RGI60-16.01339	G281855E00490S	1624	0.001248333
**GL68_UP_1**	Ecuadorian Andes	16.02.20	10:30	3.2	Antisana 14	-0.4778	-78.1582	4791	73	0.42	0.78	0.54	RGI60-16.01339	G281855E00490S	NA	0.000663333
**GL68_UP_2**	Ecuadorian Andes	16.02.20	10:30	3.2	Antisana 14	-0.4778	-78.1582	4791	73	0.42	0.78	0.54	RGI60-16.01339	G281855E00490S	NA	0.001859
**GL68_UP_3**	Ecuadorian Andes	16.02.20	10:30	3.2	Antisana 14	-0.4778	-78.1582	4791	73	0.42	0.78	0.54	RGI60-16.01339	G281855E00490S	NA	0.000298333
**GL68_DN_1**	Ecuadorian Andes	16.02.20	14:09	0.9	Antisana 14	-0.4773	-78.1592	4778	198	0.42	0.73	0.58	RGI60-16.01339	G281855E00490S	NA	0.020656667
**GL68_DN_2**	Ecuadorian Andes	16.02.20	14:09	0.9	Antisana 14	-0.4773	-78.1592	4778	198	0.42	0.73	0.58	RGI60-16.01339	G281855E00490S	NA	0.012124
**GL68_DN_3**	Ecuadorian Andes	16.02.20	14:09	0.9	Antisana 14	-0.4773	-78.1592	4778	198	0.42	0.73	0.58	RGI60-16.01339	G281855E00490S	NA	0.003749667
**GL69_UP_1**	Ecuadorian Andes	20.02.20	12:10	0.9	"Cotopaxi 1. east of refuge"	-0.6672	-78.4341	4913	42	0.54	0.95	0.57	RGI60-16.02944	G281572E00688S	3300	0.003741
**GL69_UP_2**	Ecuadorian Andes	20.02.20	12:10	0.9	"Cotopaxi 1. east of refuge"	-0.6672	-78.4341	4913	42	0.54	0.95	0.57	RGI60-16.02944	G281572E00688S	3300	0.007126667
**GL69_UP_3**	Ecuadorian Andes	20.02.20	12:10	0.9	"Cotopaxi 1. east of refuge"	-0.6672	-78.4341	4913	42	0.54	0.95	0.57	RGI60-16.02944	G281572E00688S	3300	0.007157667
**GL69_DN_1**	Ecuadorian Andes	20.02.20	14:47	4.3	"Cotopaxi 1. east of refuge"	-0.6656	-78.4332	4827	239	0.54	0.88	0.62	RGI60-16.02944	G281572E00688S	3300	0.008302667
**GL69_DN_2**	Ecuadorian Andes	20.02.20	14:47	4.3	"Cotopaxi 1. east of refuge"	-0.6656	-78.4332	4827	239	0.54	0.88	0.62	RGI60-16.02944	G281572E00688S	3300	0.004635667
**GL69_DN_3**	Ecuadorian Andes	20.02.20	14:47	4.3	"Cotopaxi 1. east of refuge"	-0.6656	-78.4332	4827	239	0.54	0.88	0.62	RGI60-16.02944	G281572E00688S	3300	0.007130667
**GL70_UP_1**	Ecuadorian Andes	21.02.20	10:50	0.4	Cotopaxi Glaciar Baja	-0.6681	-78.4405	5048	11	0.19	0.88	0.22	RGI60-16.02943	G281559E00671S	3300	0.00948
**GL70_UP_2**	Ecuadorian Andes	21.02.20	10:50	0.4	Cotopaxi Glaciar Baja	-0.6681	-78.4405	5048	11	0.19	0.88	0.22	RGI60-16.02943	G281559E00671S	3300	0.006292
**GL70_UP_3**	Ecuadorian Andes	21.02.20	10:50	0.4	Cotopaxi Glaciar Baja	-0.6681	-78.4405	5048	11	0.19	0.88	0.22	RGI60-16.02943	G281559E00671S	3300	0.004313
**GL70_DN_1**	Ecuadorian Andes	21.02.20	14:03	3	Cotopaxi Glaciar Baja	-0.6674	-78.4408	4987	98	0.19	0.82	0.23	RGI60-16.02943	G281559E00671S	3300	0.004134667

**GL70_DN_2**	Ecuadorian Andes	21.02.20	14:03	3	Cotopaxi Glaciar Baja	-0.6674	-78.4408	4987	98	0.19	0.82	0.23	RGI60-16.02943	G281559E00671S	3300	0.004719
**GL70_DN_3**	Ecuadorian Andes	21.02.20	14:03	3	Cotopaxi Glaciar Baja	-0.6674	-78.4408	4987	98	0.19	0.82	0.23	RGI60-16.02943	G281559E00671S	3300	0.006210333
**GL71_UP_1**	Ecuadorian Andes	22.02.20	10:35	4	"Cotopaxi 2. east of refuge"	-0.6673	-78.4316	4867	78	0.54	0.91	0.6	RGI60-16.02944	G281572E00688S	3300	0.000302
**GL71_UP_2**	Ecuadorian Andes	22.02.20	10:35	4	"Cotopaxi 2. east of refuge"	-0.6673	-78.4316	4867	78	0.54	0.91	0.6	RGI60-16.02944	G281572E00688S	3300	0.000880667
**GL71_UP_3**	Ecuadorian Andes	22.02.20	10:35	4	"Cotopaxi 2. east of refuge"	-0.6673	-78.4316	4867	78	0.54	0.91	0.6	RGI60-16.02944	G281572E00688S	3300	0.00011097
**GL71_DN_1**	Ecuadorian Andes	22.02.20	13:31	7.9	"Cotopaxi 2. east of refuge"	-0.666	-78.4306	4803	253	0.54	0.85	0.63	RGI60-16.02944	G281572E00688S	3300	0.000954
**GL71_DN_2**	Ecuadorian Andes	22.02.20	13:31	7.9	"Cotopaxi 2. east of refuge"	-0.666	-78.4306	4803	253	0.54	0.85	0.63	RGI60-16.02944	G281572E00688S	3300	0.001220667
**GL71_DN_3**	Ecuadorian Andes	22.02.20	13:31	7.9	"Cotopaxi 2. east of refuge"	-0.666	-78.4306	4803	253	0.54	0.85	0.63	RGI60-16.02944	G281572E00688S	3300	0.000777667
**GL72_UP_1**	Ecuadorian Andes	23.02.20	10:31	7.1	"Cotopaxi 3. east of refuge"	-0.6663	-78.4263	4757	86	0.34	0.91	0.37	RGI60-16.02944	G281572E00688S	3300	0.003733667
**GL72_UP_2**	Ecuadorian Andes	23.02.20	10:31	7.1	"Cotopaxi 3. east of refuge"	-0.6663	-78.4263	4757	86	0.34	0.91	0.37	RGI60-16.02944	G281572E00688S	3300	0.002689333
**GL72_UP_3**	Ecuadorian Andes	23.02.20	10:31	7.1	"Cotopaxi 3. east of refuge"	-0.6663	-78.4263	4757	86	0.34	0.91	0.37	RGI60-16.02944	G281572E00688S	3300	0.003858667
**GL72_DN_1**	Ecuadorian Andes	23.02.20	13:48	2.3	"Cotopaxi 3. east of refuge"	-0.6658	-78.4262	4746	141	0.34	0.85	0.4	RGI60-16.02944	G281572E00688S	3300	0.001158
**GL72_DN_2**	Ecuadorian Andes	23.02.20	13:48	2.3	"Cotopaxi 3. east of refuge"	-0.6658	-78.4262	4746	141	0.34	0.85	0.4	RGI60-16.02944	G281572E00688S	3300	0.003364333
**GL72_DN_3**	Ecuadorian Andes	23.02.20	13:48	2.3	"Cotopaxi 3. east of refuge"	-0.6658	-78.4262	4746	141	0.34	0.85	0.4	RGI60-16.02944	G281572E00688S	3300	0.001222
**GL73_UP_1**	Ecuadorian Andes	27.02.20	11:25	0.8	"Chimborazo Rock Glacier"	-1.4612	-78.7769	4541	13	0.82	0.35	2.38	RGI60-16.01311	G281198E01465S	NA	0.312053333
**GL73_UP_2**	Ecuadorian Andes	27.02.20	11:25	0.8	"Chimborazo Rock Glacier"	-1.4612	-78.7769	4541	13	0.82	0.35	2.38	RGI60-16.01311	G281198E01465S	NA	0.034551333
**GL73_UP_3**	Ecuadorian Andes	27.02.20	11:25	0.8	"Chimborazo Rock Glacier"	-1.4612	-78.7769	4541	13	0.82	0.35	2.38	RGI60-16.01311	G281198E01465S	NA	0.027755667
**GL73_DN_1**	Ecuadorian Andes	27.02.20	14:05	3.3	"Chimborazo Rock Glacier"	-1.4605	-78.773	4471	453	0.82	0.33	2.48	RGI60-16.01311	G281198E01465S	NA	0.109495667
**GL73_DN_2**	Ecuadorian Andes	27.02.20	14:05	3.3	"Chimborazo Rock Glacier"	-1.4605	-78.773	4471	453	0.82	0.33	2.48	RGI60-16.01311	G281198E01465S	NA	0.160981333

**GL73_DN_3**	Ecuadorian Andes	27.02.20	14:05	3.3	"Chimborazo Rock Glacier"	-1.4605	-78.773	4471	453	0.82	0.33	2.48	RGI60-16.01311	G281198E01465S	NA	0.122942333
**GL74_UP_1**	Ecuadorian Andes	29.02.20	11:00	1.5	"Carihuarazo"	-1.4051	-78.7559	4764	6	0.01	0.12	0.07	RGI60-16.01319	G281243E01403S	NA	0.062067667
**GL74_UP_2**	Ecuadorian Andes	29.02.20	11:00	1.5	"Carihuarazo"	-1.4051	-78.7559	4764	6	0.01	0.12	0.07	RGI60-16.01319	G281243E01403S	NA	0.261167667
**GL74_UP_3**	Ecuadorian Andes	29.02.20	11:00	1.5	"Carihuarazo"	-1.4051	-78.7559	4764	6	0.01	0.12	0.07	RGI60-16.01319	G281243E01403S	NA	0.090745333
**GL74_DN_1**	Ecuadorian Andes	29.02.20	13:54	4.2	"Carihuarazo"	-1.4062	-78.7566	4725	156	0.01	0.07	0.11	RGI60-16.01319	G281243E01403S	NA	0.075800333
**GL74_DN_2**	Ecuadorian Andes	29.02.20	13:54	4.2	"Carihuarazo"	-1.4062	-78.7566	4725	156	0.01	0.07	0.11	RGI60-16.01319	G281243E01403S	NA	0.134821
**GL74_DN_3**	Ecuadorian Andes	29.02.20	13:54	4.2	"Carihuarazo"	-1.4062	-78.7566	4725	156	0.01	0.07	0.11	RGI60-16.01319	G281243E01403S	NA	0.129302
**GL75_UP_1**	Ecuadorian Andes	01.03.20	11:34	0	"Chimborazo North"	-1.4462	-78.804	4936	20	0.96	0.77	1.25	RGI60-16.01308	G281194E01453S	NA	0.065831333
**GL75_UP_2**	Ecuadorian Andes	01.03.20	11:34	0	"Chimborazo North"	-1.4462	-78.804	4936	20	0.96	0.77	1.25	RGI60-16.01308	G281194E01453S	NA	0.102642
**GL75_UP_3**	Ecuadorian Andes	01.03.20	11:34	0	"Chimborazo North"	-1.4462	-78.804	4936	20	0.96	0.77	1.25	RGI60-16.01308	G281194E01453S	NA	0.051213333
**GL75_DN_1**	Ecuadorian Andes	01.03.20	14:07	2.9	"Chimborazo North"	-1.4456	-78.8039	4935	84	0.96	0.75	1.28	RGI60-16.01308	G281194E01453S	NA	0.074768
**GL75_DN_2**	Ecuadorian Andes	01.03.20	14:07	2.9	"Chimborazo North"	-1.4456	-78.8039	4935	84	0.96	0.75	1.28	RGI60-16.01308	G281194E01453S	NA	0.043155
**GL75_DN_3**	Ecuadorian Andes	01.03.20	14:07	2.9	"Chimborazo North"	-1.4456	-78.8039	4935	84	0.96	0.75	1.28	RGI60-16.01308	G281194E01453S	NA	0.073302667
**GL76_UP_1**	European Alps	02.07.20	10:19	1.1	Trift VS	46.1358	7.9866	2868	33	1.55	0.67	2.3	RGI60-11.02540	G008002E46138N	5435	0.002816667
**GL76_UP_2**	European Alps	02.07.20	10:19	1.1	Trift VS	46.1358	7.9866	2868	33	1.55	0.67	2.3	RGI60-11.02540	G008002E46138N	5435	0.004185
**GL76_UP_3**	European Alps	02.07.20	10:19	1.1	Trift VS	46.1358	7.9866	2868	33	1.55	0.67	2.3	RGI60-11.02540	G008002E46138N	5435	0.001846
**GL76_DN_1**	European Alps	02.07.20	13:13	2.4	Trift VS	46.1361	7.9823	2788	367	1.55	0.64	2.42	RGI60-11.02540	G008002E46138N	5435	0.001256
**GL76_DN_2**	European Alps	02.07.20	13:13	2.4	Trift VS	46.1361	7.9823	2788	367	1.55	0.64	2.42	RGI60-11.02540	G008002E46138N	5435	0.001905667
**GL76_DN_3**	European Alps	02.07.20	13:13	2.4	Trift VS	46.1361	7.9823	2788	367	1.55	0.64	2.42	RGI60-11.02540	G008002E46138N	5435	0.001520667

**GL77_UP_1**	European Alps	04.07.20	11:37	1.8	Forno	46.3352	9.7018	2254	31	5.98	0.44	13.66	RGI60-11.02245	G009697E46305N	396	0.007086333
**GL77_UP_2**	European Alps	04.07.20	11:37	1.8	Forno	46.3352	9.7018	2254	31	5.98	0.44	13.66	RGI60-11.02245	G009697E46305N	396	0.031984333
**GL77_UP_3**	European Alps	04.07.20	11:37	1.8	Forno	46.3352	9.7018	2254	31	5.98	0.44	13.66	RGI60-11.02245	G009697E46305N	396	0.00589
**GL77_DN_1**	European Alps	04.07.20	14:31	7.9	Forno	46.3431	9.7006	2223	914	5.98	0.37	16.15	RGI60-11.02245	G009697E46305N	396	0.026104333
**GL77_DN_2**	European Alps	04.07.20	14:31	7.9	Forno	46.3431	9.7006	2223	914	5.98	0.37	16.15	RGI60-11.02245	G009697E46305N	396	0.048142667
**GL77_DN_3**	European Alps	04.07.20	14:31	7.9	Forno	46.3431	9.7006	2223	914	5.98	0.37	16.15	RGI60-11.02245	G009697E46305N	396	0.158187667
**GL78_UP_1**	European Alps	05.07.20	10:40	0.3	Albigna	46.3131	9.6464	2176	58	2.6	0.37	7.04	RGI60-11.02285	G009641E46297N	1674	0
**GL78_UP_2**	European Alps	05.07.20	10:40	0.3	Albigna	46.3131	9.6464	2176	58	2.6	0.37	7.04	RGI60-11.02285	G009641E46297N	1674	0.0001463
**GL78_UP_3**	European Alps	05.07.20	10:40	0.3	Albigna	46.3131	9.6464	2176	58	2.6	0.37	7.04	RGI60-11.02285	G009641E46297N	1674	0.002778333
**GL78_DN_1**	European Alps	05.07.20	13:20	1.4	Albigna	46.3151	9.6468	2165	282	2.6	0.36	7.24	RGI60-11.02285	G009641E46297N	1674	8.93E-05
**GL78_DN_2**	European Alps	05.07.20	13:20	1.4	Albigna	46.3151	9.6468	2165	282	2.6	0.36	7.24	RGI60-11.02285	G009641E46297N	1674	0.00054316
**GL78_DN_3**	European Alps	05.07.20	13:20	1.4	Albigna	46.3151	9.6468	2165	282	2.6	0.36	7.24	RGI60-11.02285	G009641E46297N	1674	1.83E-06
**GL79_UP_1**	European Alps	06.07.20	09:49	0.1	Morteratsch	46.4197	9.9338	2176	2	15.77	0.62	25.24	RGI60-11.01946	G009641E46297N	1673	0.007321333
**GL79_UP_2**	European Alps	06.07.20	09:49	0.1	Morteratsch	46.4197	9.9338	2176	2	15.77	0.62	25.24	RGI60-11.01946	G009641E46297N	1673	0.020916
**GL79_UP_3**	European Alps	06.07.20	09:49	0.1	Morteratsch	46.4197	9.9338	2176	2	15.77	0.62	25.24	RGI60-11.01946	G009641E46297N	1673	0.014758667
**GL79_DN_1**	European Alps	06.07.20	13:15	3.3	Morteratsch	46.4227	9.9335	2063	336	15.77	0.61	25.71	RGI60-11.01946	G009641E46297N	1673	0.038753
**GL79_DN_2**	European Alps	06.07.20	13:15	3.3	Morteratsch	46.4227	9.9335	2063	336	15.77	0.61	25.71	RGI60-11.01946	G009641E46297N	1673	0.004444667
**GL79_DN_3**	European Alps	06.07.20	13:15	3.3	Morteratsch	46.4227	9.9335	2063	336	15.77	0.61	25.71	RGI60-11.01946	G009641E46297N	1673	0.004879333
**GL80_UP_1**	European Alps	07.07.20	10:53	3.2	Roseg	46.385	9.8419	2276	119	3.34	0.75	4.43	RGI60-11.02119	G009860E46370N	406	0.009599

**GL80_UP_2**	European Alps	07.07.20	10:53	3.2	Roseg	46.385	9.8419	2276	119	3.34	0.75	4.43	RGI60-11.02119	G009860E46370N	406	0.010347333
**GL80_UP_3**	European Alps	07.07.20	10:53	3.2	Roseg	46.385	9.8419	2276	119	3.34	0.75	4.43	RGI60-11.02119	G009860E46370N	406	0.016285667
**GL80_DN_1**	European Alps	07.07.20	13:48	5.8	Roseg	46.3909	9.8443	2161	800	3.34	0.28	11.82	RGI60-11.02119	G009860E46370N	406	0.001927333
**GL80_DN_2**	European Alps	07.07.20	13:48	5.8	Roseg	46.3909	9.8443	2161	800	3.34	0.28	11.82	RGI60-11.02119	G009860E46370N	406	0.013211333
**GL80_DN_3**	European Alps	07.07.20	13:48	5.8	Roseg	46.3909	9.8443	2161	800	3.34	0.28	11.82	RGI60-11.02119	G009860E46370N	406	0.010058333
**GL81_UP_1**	European Alps	08.07.20	11:30	0.8	Tschierva	46.4035	9.8695	2326	69	6.11	0.6	10.19	RGI60-11.02051	G009886E46384N	405 (4597)	0.000838
**GL81_UP_2**	European Alps	08.07.20	11:30	0.8	Tschierva	46.4035	9.8695	2326	69	6.11	0.6	10.19	RGI60-11.02051	G009886E46384N	405 (4597)	0.004274
**GL81_UP_3**	European Alps	08.07.20	11:30	0.8	Tschierva	46.4035	9.8695	2326	69	6.11	0.6	10.19	RGI60-11.02051	G009886E46384N	405 (4597)	0.001894333
**GL81_DN_1**	European Alps	08.07.20	15:20	4.8	Tschierva	46.4122	9.8576	2098	1402	6.11	0.55	11.14	RGI60-11.02051	G009886E46384N	405 (4597)	0.005237667
**GL81_DN_2**	European Alps	08.07.20	15:20	4.8	Tschierva	46.4122	9.8576	2098	1402	6.11	0.55	11.14	RGI60-11.02051	G009886E46384N	405 (4597)	0.029032333
**GL81_DN_3**	European Alps	08.07.20	15:20	4.8	Tschierva	46.4122	9.8576	2098	1402	6.11	0.55	11.14	RGI60-11.02051	G009886E46384N	405 (4597)	0.006469
**GL82_UP_1**	European Alps	10.07.20	09:45	1	Silvretta	46.8559	10.0569	2474	61	2.36	0.66	3.56	RGI60-11.00804	G010084E46850N	408	0.039153
**GL82_UP_2**	European Alps	10.07.20	09:45	1	Silvretta	46.8559	10.0569	2474	61	2.36	0.66	3.56	RGI60-11.00804	G010084E46850N	408	0.022635
**GL82_UP_3**	European Alps	10.07.20	09:45	1	Silvretta	46.8559	10.0569	2474	61	2.36	0.66	3.56	RGI60-11.00804	G010084E46850N	408	0.004618
**GL82_DN_1**	European Alps	10.07.20	12:28	0.8	Silvretta	46.8545	10.0542	2430	319	2.36	0.61	3.88	RGI60-11.00804	G010084E46850N	408	0.003154
**GL82_DN_2**	European Alps	10.07.20	12:28	0.8	Silvretta	46.8545	10.0542	2430	319	2.36	0.61	3.88	RGI60-11.00804	G010084E46850N	408	0.016642
**GL82_DN_3**	European Alps	10.07.20	12:28	0.8	Silvretta	46.8545	10.0542	2430	319	2.36	0.61	3.88	RGI60-11.00804	G010084E46850N	408	0.003450667
**GL83_UP_1**	European Alps	12.07.20	10:50	0.5	Hintereis F.	46.8169	10.7994	2489	331	7.79	0.49	15.97	RGI60-11.00897	G010758E46800N	491	0.001213333
**GL83_UP_2**	European Alps	12.07.20	10:50	0.5	Hintereis F.	46.8169	10.7994	2489	331	7.79	0.49	15.97	RGI60-11.00897	G010758E46800N	491	0.000235

**GL83_UP_3**	European Alps	12.07.20	10:50	0.5	Hintereis F.	46.8169	10.7994	2489	331	7.79	0.49	15.97	RGI60-11.00897	G010758E46800N	491	0.0003233
**GL83_DN_1**	European Alps	12.07.20	14:00	1.7	Hintereis F.	46.8199	10.8056	2410	914	7.88	0.44	17.83	RGI60-11.00897	G010758E46800N	491	0.000197667
**GL83_DN_2**	European Alps	12.07.20	14:00	1.7	Hintereis F.	46.8199	10.8056	2410	914	7.88	0.44	17.83	RGI60-11.00897	G010758E46800N	491	0.000439333
**GL83_DN_3**	European Alps	12.07.20	14:00	1.7	Hintereis F.	46.8199	10.8056	2410	914	7.88	0.44	17.83	RGI60-11.00897	G010758E46800N	491	0.001820667
**GL84_UP_1**	European Alps	13.07.20	11:32	1.3	Niederjoch F.	46.7752	10.8593	2930	514	0.39	0.4	0.96	RGI60-11.00992	G010867E46769N	516	0.002591667
**GL84_UP_2**	European Alps	13.07.20	11:32	1.3	Niederjoch F.	46.7752	10.8593	2930	514	0.39	0.4	0.96	RGI60-11.00992	G010867E46769N	516	0.002442
**GL84_UP_3**	European Alps	13.07.20	11:32	1.3	Niederjoch F.	46.7752	10.8593	2930	514	0.39	0.4	0.96	RGI60-11.00992	G010867E46769N	516	0.001356667
**GL84_DN_1**	European Alps	13.07.20	14:44	2.3	Niederjoch F.	46.7792	10.8603	2829	965	0.39	0.3	1.3	RGI60-11.00992	G010867E46769N	516	0.000424333
**GL84_DN_2**	European Alps	13.07.20	14:44	2.3	Niederjoch F.	46.7792	10.8603	2829	965	0.39	0.3	1.3	RGI60-11.00992	G010867E46769N	516	0.000803667
**GL84_DN_3**	European Alps	13.07.20	14:44	2.3	Niederjoch F.	46.7792	10.8603	2829	965	0.39	0.3	1.3	RGI60-11.00992	G010867E46769N	516	0.000364333
**GL85_UP_1**	European Alps	14.07.20	10:10	0.1	Tiefenbach F.	46.9147	10.9339	2929	84	0.31	0.84	0.37	RGI60-11.00674	G010927E46919N	NA	0.030283333
**GL85_UP_2**	European Alps	14.07.20	10:10	0.1	Tiefenbach F.	46.9147	10.9339	2929	84	0.31	0.84	0.37	RGI60-11.00674	G010927E46919N	NA	0.024696333
**GL85_UP_3**	European Alps	14.07.20	10:10	0.1	Tiefenbach F.	46.9147	10.9339	2929	84	0.31	0.84	0.37	RGI60-11.00674	G010927E46919N	NA	0.033631333
**GL85_DN_1**	European Alps	14.07.20	12:24	3.9	Tiefenbach F.	46.9149	10.9356	2870	215	0.32	0.75	0.42	RGI60-11.00674	G010927E46919N	NA	0.011996
**GL85_DN_2**	European Alps	14.07.20	12:24	3.9	Tiefenbach F.	46.9149	10.9356	2870	215	0.32	0.75	0.42	RGI60-11.00674	G010927E46919N	NA	0.017136333
**GL85_DN_3**	European Alps	14.07.20	12:24	3.9	Tiefenbach F.	46.9149	10.9356	2870	215	0.32	0.75	0.42	RGI60-11.00674	G010927E46919N	NA	0.010643333
**GL86_UP_1**	Scandinavian Mountains	07.08.20	12:07	2.7	Westbreen	69.4734	20.0001	393	554	5	0.54	9.29	RGI60-08.00335	G019954E69491N	NA	0.000423667
**GL86_UP_2**	Scandinavian Mountains	07.08.20	12:07	2.7	Westbreen	69.4734	20.0001	393	554	5	0.54	9.29	RGI60-08.00335	G019954E69491N	NA	0.003290667
**GL86_UP_3**	Scandinavian Mountains	07.08.20	12:07	2.7	Westbreen	69.4734	20.0001	393	554	5	0.54	9.29	RGI60-08.00335	G019954E69491N	NA	0.001005333

**GL86_DN_1**	Scandinavian Mountains	07.08.20	14:48	5.4	Westbreen	69.4723	20.0078	351	883	5	0.53	9.46	RGI60-08.00335	G019954E69491N	NA	0.007845333
**GL86_DN_2**	Scandinavian Mountains	07.08.20	14:48	5.4	Westbreen	69.4723	20.0078	351	883	5	0.53	9.46	RGI60-08.00335	G019954E69491N	NA	0.009432333
**GL86_DN_3**	Scandinavian Mountains	07.08.20	14:48	5.4	Westbreen	69.4723	20.0078	351	883	5	0.53	9.46	RGI60-08.00335	G019954E69491N	NA	0.003227
**GL87_UP_1**	Scandinavian Mountains	08.08.20	11:30	2.1	Midbreen	69.4603	19.9521	517	46	3.18	0.57	5.54	RGI60-08.03209	G019923E69469N	NA	0.007644667
**GL87_UP_2**	Scandinavian Mountains	08.08.20	11:30	2.1	Midbreen	69.4603	19.9521	517	46	3.18	0.57	5.54	RGI60-08.03209	G019923E69469N	NA	0.020138
**GL87_UP_3**	Scandinavian Mountains	08.08.20	11:30	2.1	Midbreen	69.4603	19.9521	517	46	3.18	0.57	5.54	RGI60-08.03209	G019923E69469N	NA	0.004865
**GL87_DN_1**	Scandinavian Mountains	08.08.20	15:06	2.7	Midbreen	69.4584	19.9596	400	415	3.22	0.55	5.88	RGI60-08.03209	G019923E69469N	NA	0.017859667
**GL87_DN_2**	Scandinavian Mountains	08.08.20	15:06	2.7	Midbreen	69.4584	19.9596	400	415	3.22	0.55	5.88	RGI60-08.03209	G019923E69469N	NA	0.005084667
**GL87_DN_3**	Scandinavian Mountains	08.08.20	15:06	2.7	Midbreen	69.4584	19.9596	400	415	3.22	0.55	5.88	RGI60-08.03209	G019923E69469N	NA	0.017699
**GL88_UP_1**	Scandinavian Mountains	09.08.20	12:00	0.9	"Southeast of Fugldalsvatnet"	69.4854	19.8359	446	57	1.1	0.36	3.1	RGI60-08.03199	G019849E69478N	NA	0.000217333
**GL88_UP_2**	Scandinavian Mountains	09.08.20	12:00	0.9	"Southeast of Fugldalsvatnet"	69.4854	19.8359	446	57	1.1	0.36	3.1	RGI60-08.03199	G019849E69478N	NA	0
**GL88_UP_3**	Scandinavian Mountains	09.08.20	12:00	0.9	"Southeast of Fugldalsvatnet"	69.4854	19.8359	446	57	1.1	0.36	3.1	RGI60-08.03199	G019849E69478N	NA	0
**GL88_DN_1**	Scandinavian Mountains	09.08.20	14:00	2.7	"Southeast of Fugldalsvatnet"	69.4869	19.8298	385	351	1.1	0.33	3.35	RGI60-08.03199	G019849E69478N	NA	0.000236
**GL88_DN_2**	Scandinavian Mountains	09.08.20	14:00	2.7	"Southeast of Fugldalsvatnet"	69.4869	19.8298	385	351	1.1	0.33	3.35	RGI60-08.03199	G019849E69478N	NA	0.000246333
**GL88_DN_3**	Scandinavian Mountains	09.08.20	14:00	2.7	"Southeast of Fugldalsvatnet"	69.4869	19.8298	385	351	1.1	0.33	3.35	RGI60-08.03199	G019849E69478N	NA	0.000818667
**GL89_UP_1**	Scandinavian Mountains	12.08.20	11:30	0.6	"Northwest of Blaaisen"	69.4748	19.7763	946	128	0.21	0.24	0.89	RGI60-08.03193	G019786E69474N	NA	0.003163
**GL89_UP_2**	Scandinavian Mountains	12.08.20	11:30	0.6	"Northwest of Blaaisen"	69.4748	19.7763	946	128	0.21	0.24	0.89	RGI60-08.03193	G019786E69474N	NA	0.002521667
**GL89_UP_3**	Scandinavian Mountains	12.08.20	11:30	0.6	"Northwest of Blaaisen"	69.4748	19.7763	946	128	0.21	0.24	0.89	RGI60-08.03193	G019786E69474N	NA	0.002125
**GL89_DN_1**	Scandinavian Mountains	12.08.20	14:30	3.9	"Northwest of Blaaisen"	69.4749	19.7736	939	233	0.21	0.23	0.94	RGI60-08.03193	G019786E69474N	NA	0.001274333

**GL89_DN_2**	Scandinavian Mountains	12.08.20	14:30	3.9	"Northwest of Blaaisen"	69.4749	19.7736	939	233	0.21	0.23	0.94	RGI60-08.03193	G019786E69474N	NA	0.000557333
**GL89_DN_3**	Scandinavian Mountains	12.08.20	14:30	3.9	"Northwest of Blaaisen"	69.4749	19.7736	939	233	0.21	0.23	0.94	RGI60-08.03193	G019786E69474N	NA	0.000632333
**GL90_UP_1**	Scandinavian Mountains	20.08.20	09:55	0.7	Tuftebreen	61.6586	7.1539	859	54	6.43	0.8	8.05	RGI60-08.01125	G007087E61677N	3352	0.008286
**GL90_UP_2**	Scandinavian Mountains	20.08.20	09:55	0.7	Tuftebreen	61.6586	7.1539	859	54	6.43	0.8	8.05	RGI60-08.01125	G007087E61677N	3352	0.009953333
**GL90_UP_3**	Scandinavian Mountains	20.08.20	09:55	0.7	Tuftebreen	61.6586	7.1539	859	54	6.43	0.8	8.05	RGI60-08.01125	G007087E61677N	3352	0.000706
**GL90_DN_1**	Scandinavian Mountains	20.08.20	12:40	1.5	Tuftebreen	61.6565	7.1551	792	303	6.43	0.78	8.24	RGI60-08.01125	G007087E61677N	3352	0.046184333
**GL90_DN_2**	Scandinavian Mountains	20.08.20	12:40	1.5	Tuftebreen	61.6565	7.1551	792	303	6.43	0.78	8.24	RGI60-08.01125	G007087E61677N	3352	0.021440333
**GL90_DN_3**	Scandinavian Mountains	20.08.20	12:40	1.5	Tuftebreen	61.6565	7.1551	792	303	6.43	0.78	8.24	RGI60-08.01125	G007087E61677N	3352	0.062398
**GL91_UP_1**	Scandinavian Mountains	21.08.20	09:46	1	Fabergstolbreen	61.7124	7.2921	779	26	17.49	0.89	19.69	RGI60-08.01133	G007202E61739N	289	0.009305333
**GL91_UP_2**	Scandinavian Mountains	21.08.20	09:46	1	Fabergstolbreen	61.7124	7.2921	779	26	17.49	0.89	19.69	RGI60-08.01133	G007202E61739N	289	0.007920667
**GL91_UP_3**	Scandinavian Mountains	21.08.20	09:46	1	Fabergstolbreen	61.7124	7.2921	779	26	17.49	0.89	19.69	RGI60-08.01133	G007202E61739N	289	0.007206333
**GL91_DN_1**	Scandinavian Mountains	21.08.20	12:32	5.6	Fabergstolbreen	61.7132	7.2963	716	262	17.5	0.89	19.74	RGI60-08.01133	G007202E61739N	289	0.097552
**GL91_DN_2**	Scandinavian Mountains	21.08.20	12:32	5.6	Fabergstolbreen	61.7132	7.2963	716	262	17.5	0.89	19.74	RGI60-08.01133	G007202E61739N	289	0.026044
**GL91_DN_3**	Scandinavian Mountains	21.08.20	12:32	5.6	Fabergstolbreen	61.7132	7.2963	716	262	17.5	0.89	19.74	RGI60-08.01133	G007202E61739N	289	0.007865333
**GL92_UP_1**	Scandinavian Mountains	22.08.20	09:50	2.9	Nigardsbreen	61.6774	7.2073	363	483	44.45	0.82	54.3	RGI60-08.01126	G007099E61715N	290	0.011690333
**GL92_UP_2**	Scandinavian Mountains	22.08.20	09:50	2.9	Nigardsbreen	61.6774	7.2073	363	483	44.45	0.82	54.3	RGI60-08.01126	G007099E61715N	290	0.032721667
**GL92_UP_3**	Scandinavian Mountains	22.08.20	09:50	2.9	Nigardsbreen	61.6774	7.2073	363	483	44.45	0.82	54.3	RGI60-08.01126	G007099E61715N	290	0.042679667
**GL92_DN_1**	Scandinavian Mountains	22.08.20	12:11	2.2	Nigardsbreen	61.676	7.2118	275	767	45.01	0.79	57.14	RGI60-08.01126	G007099E61715N	290	0.039196333
**GL92_DN_2**	Scandinavian Mountains	22.08.20	12:11	2.2	Nigardsbreen	61.676	7.2118	275	767	45.01	0.79	57.14	RGI60-08.01126	G007099E61715N	290	0.006247667

**GL92_DN_3**	Scandinavian Mountains	22.08.20	12:11	2.2	Nigardsbreen	61.676	7.2118	275	767	45.01	0.79	57.14	RGI60-08.01126	G007099E61715N	290	0.016971
**GL93_UP_1**	Scandinavian Mountains	23.08.20	10:02	0.5	Boverbreen	61.5564	8.0515	1447	1	0.76	0.76	1.01	RGI60-08.02144	G008095E61549N	2298	0.000924333
**GL93_UP_2**	Scandinavian Mountains	23.08.20	10:02	0.5	Boverbreen	61.5564	8.0515	1447	1	0.76	0.76	1.01	RGI60-08.02144	G008095E61549N	2298	0.001550667
**GL93_UP_3**	Scandinavian Mountains	23.08.20	10:02	0.5	Boverbreen	61.5564	8.0515	1447	1	0.76	0.76	1.01	RGI60-08.02144	G008095E61549N	2298	0.021861
**GL93_DN_1**	Scandinavian Mountains	23.08.20	12:38	2.9	Boverbreen	61.5571	8.0435	1368	431	0.78	0.63	1.24	RGI60-08.02144	G008095E61549N	2298	0.02064
**GL93_DN_2**	Scandinavian Mountains	23.08.20	12:38	2.9	Boverbreen	61.5571	8.0435	1368	431	0.78	0.63	1.24	RGI60-08.02144	G008095E61549N	2298	0.003937667
**GL93_DN_3**	Scandinavian Mountains	23.08.20	12:38	2.9	Boverbreen	61.5571	8.0435	1368	431	0.78	0.63	1.24	RGI60-08.02144	G008095E61549N	2298	0.016128333
**GL94_UP_1**	Scandinavian Mountains	24.08.20	09:57	0.6	Storbreen	61.5818	8.1625	1441	17	2.61	0.77	3.39	RGI60-08.00312	G008132E61573N	302	0.001236333
**GL94_UP_2**	Scandinavian Mountains	24.08.20	09:57	0.6	Storbreen	61.5818	8.1625	1441	17	2.61	0.77	3.39	RGI60-08.00312	G008132E61573N	302	0.000897667
**GL94_UP_3**	Scandinavian Mountains	24.08.20	09:57	0.6	Storbreen	61.5818	8.1625	1441	17	2.61	0.77	3.39	RGI60-08.00312	G008132E61573N	302	0.010917
**GL94_DN_1**	Scandinavian Mountains	24.08.20	12:22	0.6	Storbreen	61.5841	8.1654	1365	312	2.61	0.76	3.44	RGI60-08.00312	G008132E61573N	302	0.000459333
**GL94_DN_2**	Scandinavian Mountains	24.08.20	12:22	0.6	Storbreen	61.5841	8.1654	1365	312	2.61	0.76	3.44	RGI60-08.00312	G008132E61573N	302	0.001558
**GL94_DN_3**	Scandinavian Mountains	24.08.20	12:22	0.6	Storbreen	61.5841	8.1654	1365	312	2.61	0.76	3.44	RGI60-08.00312	G008132E61573N	302	0.001092333
**GL95_UP_1**	Scandinavian Mountains	25.08.20	10:46	0.5	Storjuvbreen	61.6653	8.2987	1396	42	4.35	0.54	8.06	RGI60-08.00860	G008289E61636N	2308	0.002201
**GL95_UP_2**	Scandinavian Mountains	25.08.20	10:46	0.5	Storjuvbreen	61.6653	8.2987	1396	42	4.35	0.54	8.06	RGI60-08.00860	G008289E61636N	2308	0.001295
**GL95_UP_3**	Scandinavian Mountains	25.08.20	10:46	0.5	Storjuvbreen	61.6653	8.2987	1396	42	4.35	0.54	8.06	RGI60-08.00860	G008289E61636N	2308	0.001654333
**GL95_DN_1**	Scandinavian Mountains	25.08.20	12:50	1	Storjuvbreen	61.6679	8.2997	1357	331	4.35	0.47	9.29	RGI60-08.00860	G008289E61636N	2308	0.000996667
**GL95_DN_2**	Scandinavian Mountains	25.08.20	12:50	1	Storjuvbreen	61.6679	8.2997	1357	331	4.35	0.47	9.29	RGI60-08.00860	G008289E61636N	2308	0.001747667
**GL95_DN_3**	Scandinavian Mountains	25.08.20	12:50	1	Storjuvbreen	61.6679	8.2997	1357	331	4.35	0.47	9.29	RGI60-08.00860	G008289E61636N	2308	0.000819

**GL96_UP_1**	European Alps	15.09.20	11:00	0.2	Miage	45.7883	6.8885	1812	72	11.13	0.46	24.07	RGI60-11.03005	G006846E45813N	613	0.001888
**GL96_UP_2**	European Alps	15.09.20	11:00	0.2	Miage	45.7883	6.8885	1812	72	11.13	0.46	24.07	RGI60-11.03005	G006846E45813N	613	0.001498267
**GL96_UP_3**	European Alps	15.09.20	11:00	0.2	Miage	45.7883	6.8885	1812	72	11.13	0.46	24.07	RGI60-11.03005	G006846E45813N	613	0.000114667
**GL96_DN_1**	European Alps	15.09.20	14:00	0.5	Miage	45.7894	6.891	1766	304	11.15	0.46	24.19	RGI60-11.03005	G006846E45813N	613	0.001952
**GL96_DN_2**	European Alps	15.09.20	14:00	0.5	Miage	45.7894	6.891	1766	304	11.15	0.46	24.19	RGI60-11.03005	G006846E45813N	613	0
**GL96_DN_3**	European Alps	15.09.20	14:00	0.5	Miage	45.7894	6.891	1766	304	11.15	0.46	24.19	RGI60-11.03005	G006846E45813N	613	0
**GL97_UP_1**	European Alps	16.09.20	12:36	0.6	Très la tête	45.7888	6.7484	2037	85	7.54	0.46	16.44	RGI60-11.03651	G006784E45784N	1314	0.000509
**GL97_UP_2**	European Alps	16.09.20	12:36	0.6	Très la tête	45.7888	6.7484	2037	85	7.54	0.46	16.44	RGI60-11.03651	G006784E45784N	1314	0.003220667
**GL97_UP_3**	European Alps	16.09.20	12:36	0.6	Très la tête	45.7888	6.7484	2037	85	7.54	0.46	16.44	RGI60-11.03651	G006784E45784N	1314	0.001741
**GL97_DN_1**	European Alps	16.09.20	15:00	0.9	Très la tête	45.7892	6.747	2014	201	7.54	0.45	16.64	RGI60-11.03651	G006784E45784N	1314	0.003143
**GL97_DN_2**	European Alps	16.09.20	15:00	0.9	Très la tête	45.7892	6.747	2014	201	7.54	0.45	16.64	RGI60-11.03651	G006784E45784N	1314	0.005717333
**GL97_DN_3**	European Alps	16.09.20	15:00	0.9	Très la tête	45.7892	6.747	2014	201	7.54	0.45	16.64	RGI60-11.03651	G006784E45784N	1314	0.000925667
**GL98_UP_1**	European Alps	17.09.20	10:44	1.3	Mer de glace	45.9357	6.9223	1591	64	36.91	0.54	68.59	RGI60-11.03643	G006934E45883N	353	0.041361
**GL98_UP_2**	European Alps	17.09.20	10:44	1.3	Mer de glace	45.9357	6.9223	1591	64	36.91	0.54	68.59	RGI60-11.03643	G006934E45883N	353	0.079373
**GL98_UP_3**	European Alps	17.09.20	10:44	1.3	Mer de glace	45.9357	6.9223	1591	64	36.91	0.54	68.59	RGI60-11.03643	G006934E45883N	353	0.014773
**GL98_DN_1**	European Alps	17.09.20	13:29	2.5	Mer de glace	45.9375	6.9224	1525	265	36.91	0.53	69.24	RGI60-11.03643	G006934E45883N	353	0.082808333
**GL98_DN_2**	European Alps	17.09.20	13:29	2.5	Mer de glace	45.9375	6.9224	1525	265	36.91	0.53	69.24	RGI60-11.03643	G006934E45883N	353	0.077471667
**GL98_DN_3**	European Alps	17.09.20	13:29	2.5	Mer de glace	45.9375	6.9224	1525	265	36.91	0.53	69.24	RGI60-11.03643	G006934E45883N	353	0.074099333
**GL99_UP_1**	European Alps	18.09.20	09:40	0	Pélerins	45.8973	6.8842	2244	82	0.94	0.38	2.5	RGI60-11.03389	G006889E45892N	NA	0.055245

**GL99_UP_2**	European Alps	18.09.20	09:40	0	Pélerins	45.8973	6.8842	2244	82	0.94	0.38	2.5	RGI60-11.03389	G006889E45892N	NA	0.022719
**GL99_UP_3**	European Alps	18.09.20	09:40	0	Pélerins	45.8973	6.8842	2244	82	0.94	0.38	2.5	RGI60-11.03389	G006889E45892N	NA	0.055635
**GL99_DN_1**	European Alps	18.09.20	12:18	0.6	Pélerins	45.8982	6.8827	2205	238	0.94	0.37	2.54	RGI60-11.03389	G006889E45892N	NA	0.024062667
**GL99_DN_2**	European Alps	18.09.20	12:18	0.6	Pélerins	45.8982	6.8827	2205	238	0.94	0.37	2.54	RGI60-11.03389	G006889E45892N	NA	0.012273
**GL99_DN_3**	European Alps	18.09.20	12:18	0.6	Pélerins	45.8982	6.8827	2205	238	0.94	0.37	2.54	RGI60-11.03389	G006889E45892N	NA	0.108285
**GL100_UP_1**	European Alps	29.09.20	12:17	1.2	Chardon	44.8843	6.2934	2301	101	1.22	0.27	4.46	RGI60-11.03817	G006302E44881N	NA	0.001672
**GL100_UP_2**	European Alps	29.09.20	12:17	1.2	Chardon	44.8843	6.2934	2301	101	1.22	0.27	4.46	RGI60-11.03817	G006302E44881N	NA	0.002788333
**GL100_UP_3**	European Alps	29.09.20	12:17	1.2	Chardon	44.8843	6.2934	2301	101	1.22	0.27	4.46	RGI60-11.03817	G006302E44881N	NA	0.001655333
**GL100_DN_1**	European Alps	29.09.20	15:00	2.2	Chardon	44.8863	6.2941	2234	336	1.24	0.25	4.96	RGI60-11.03817	G006302E44881N	NA	0.008251
**GL100_DN_2**	European Alps	29.09.20	15:00	2.2	Chardon	44.8863	6.2941	2234	336	1.24	0.25	4.96	RGI60-11.03817	G006302E44881N	NA	0.003288333
**GL100_DN_3**	European Alps	29.09.20	15:00	2.2	Chardon	44.8863	6.2941	2234	336	1.24	0.25	4.96	RGI60-11.03817	G006302E44881N	NA	0.004957
**GL101_UP_1**	European Alps	30.09.20	11:59	0.4	Des étages	44.8985	6.2624	2440	58	0.83	0.3	2.75	RGI60-11.03694	G006263E44892N	NA	0.000373667
**GL101_UP_2**	European Alps	30.09.20	11:59	0.4	Des étages	44.8985	6.2624	2440	58	0.83	0.3	2.75	RGI60-11.03694	G006263E44892N	NA	2.43E-05
**GL101_UP_3**	European Alps	30.09.20	11:59	0.4	Des étages	44.8985	6.2624	2440	58	0.83	0.3	2.75	RGI60-11.03694	G006263E44892N	NA	0.000553767
**GL101_DN_1**	European Alps	30.09.20	15:30	5.4	Des étages	44.9148	6.2582	2008	1899	1.25	0.13	9.66	RGI60-11.03694	G006263E44892N	NA	0.038685
**GL101_DN_2**	European Alps	30.09.20	15:30	5.4	Des étages	44.9148	6.2582	2008	1899	1.25	0.13	9.66	RGI60-11.03694	G006263E44892N	NA	0.028025
**GL101_DN_3**	European Alps	30.09.20	15:30	5.4	Des étages	44.9148	6.2582	2008	1899	1.25	0.13	9.66	RGI60-11.03694	G006263E44892N	NA	0.015758
**GL102_UP_1**	European Alps	01.10.20	11:50	0	Bonne Pierre	44.9358	6.3183	2436	24	1.95	0.31	6.32	RGI60-11.03810	G006341E44929N	NA	0.000621667
**GL102_UP_2**	European Alps	01.10.20	11:50	0	Bonne Pierre	44.9358	6.3183	2436	24	1.95	0.31	6.32	RGI60-11.03810	G006341E44929N	NA	0.000840667

**GL102_UP_3**	European Alps	01.10.20	11:50	0	Bonne Pierre	44.9358	6.3183	2436	24	1.95	0.31	6.32	RGI60-11.03810	G006341E44929N	NA	0.001958333
**GL102_DN_1**	European Alps	01.10.20	15:36	3.9	Bonne Pierre	44.9387	6.2986	1880	1612	1.96	0.21	9.44	RGI60-11.03810	G006341E44929N	NA	0.056078333
**GL102_DN_2**	European Alps	01.10.20	15:36	3.9	Bonne Pierre	44.9387	6.2986	1880	1612	1.96	0.21	9.44	RGI60-11.03810	G006341E44929N	NA	0.055441333
**GL102_DN_3**	European Alps	01.10.20	15:36	3.9	Bonne Pierre	44.9387	6.2986	1880	1612	1.96	0.21	9.44	RGI60-11.03810	G006341E44929N	NA	0.028549
**GL103_UP_1**	Himalayas	17.03.21	11:16	0.2	Lirung	28.2298	85.56236	4062	432	5.52	0.34	16.301251	RGI60-15.04045	G085556E28239N	NA	0.044590333
**GL103_UP_2**	Himalayas	17.03.21	11:16	0.2	Lirung	28.2298	85.56236	4062	432	5.52	0.34	16.301251	RGI60-15.04045	G085556E28239N	NA	0.094442667
**GL103_UP_3**	Himalayas	17.03.21	11:16	0.2	Lirung	28.2298	85.56236	4062	432	5.52	0.34	16.301251	RGI60-15.04045	G085556E28239N	NA	0.054166
**GL103_DN_1**	Himalayas	17.03.21	13:50	5.6	Lirung	28.22356	85.56226	3989	1568	5.52	0.29	18.71892	RGI60-15.04045	G085556E28239N	NA	0.082834
**GL103_DN_2**	Himalayas	17.03.21	13:50	5.6	Lirung	28.22356	85.56226	3989	1568	5.52	0.29	18.71892	RGI60-15.04045	G085556E28239N	NA	0.064969
**GL103_DN_3**	Himalayas	17.03.21	13:50	5.6	Lirung	28.22356	85.56226	3989	1568	5.52	0.29	18.71892	RGI60-15.04045	G085556E28239N	NA	0.024272333
**GL104_UP_1**	Himalayas	18.03.21	10:20	0	Kyimoshung	28.23401	85.57678	4369	103	3.97	0.68	5.805793	RGI60-15.04075	G085573E28258N	3907	0.004992333
**GL104_UP_2**	Himalayas	18.03.21	10:20	0	Kyimoshung	28.23401	85.57678	4369	103	3.97	0.68	5.805793	RGI60-15.04075	G085573E28258N	3907	0.009870333
**GL104_UP_3**	Himalayas	18.03.21	10:20	0	Kyimoshung	28.23401	85.57678	4369	103	3.97	0.68	5.805793	RGI60-15.04075	G085573E28258N	3907	0.003667333
**GL104_DN_1**	Himalayas	18.03.21	13:15	3.4	Kyimoshung	28.23195	85.57248	4197	943	3.97	0.67	5.926477	RGI60-15.04075	G085573E28258N	3907	0.017308667
**GL104_DN_2**	Himalayas	18.03.21	13:15	3.4	Kyimoshung	28.23195	85.57248	4197	943	3.97	0.67	5.926477	RGI60-15.04075	G085573E28258N	3907	0.018543
**GL104_DN_3**	Himalayas	18.03.21	13:15	3.4	Kyimoshung	28.23195	85.57248	4197	943	3.97	0.67	5.926477	RGI60-15.04075	G085573E28258N	3907	0.025178
**GL105_UP_1**	Himalayas	21.03.21	11:07	0	Langtang	28.23321	85.69581	4488	904	48.08	0.46	105.5177	RGI60-15.04121	G085670E28312N	NA	0.011783333
**GL105_UP_2**	Himalayas	21.03.21	11:07	0	Langtang	28.23321	85.69581	4488	904	48.08	0.46	105.5177	RGI60-15.04121	G085670E28312N	NA	0.000891667
**GL105_UP_3**	Himalayas	21.03.21	11:07	0	Langtang	28.23321	85.69581	4488	904	48.08	0.46	105.5177	RGI60-15.04121	G085670E28312N	NA	0.003096333

**GL105_DN_1**	Himalayas	21.03.21	13:44	0.4	Langtang	28.23187	85.69202	4442	803	48.08	0.43	111.1479	RGI60-15.04121	G085670E28312N	NA	0.005982667
**GL105_DN_2**	Himalayas	21.03.21	13:44	0.4	Langtang	28.23187	85.69202	4442	803	48.08	0.43	111.1479	RGI60-15.04121	G085670E28312N	NA	0.003256333
**GL105_DN_3**	Himalayas	21.03.21	13:44	0.4	Langtang	28.23187	85.69202	4442	803	48.08	0.43	111.1479	RGI60-15.04121	G085670E28312N	NA	0.004253667
**GL106_UP_1**	Himalayas	22.03.21	09:35	0.6	Langshisha	28.19602	85.69055	4426	60	21.3	0.41	51.90574	RGI60-15.04176	G085747E28200N	NA	0.011100333
**GL106_UP_2**	Himalayas	22.03.21	09:35	0.6	Langshisha	28.19602	85.69055	4426	60	21.3	0.41	51.90574	RGI60-15.04176	G085747E28200N	NA	0.001817333
**GL106_UP_3**	Himalayas	22.03.21	09:35	0.6	Langshisha	28.19602	85.69055	4426	60	21.3	0.41	51.90574	RGI60-15.04176	G085747E28200N	NA	0.001688333
**GL106_DN_1**	Himalayas	22.03.21	13:00	4	Langshisha	28.20647	85.67937	4234	600	21.3	0.38	55.45015	RGI60-15.04176	G085747E28200N	NA	0.087935667
**GL106_DN_2**	Himalayas	22.03.21	13:00	4	Langshisha	28.20647	85.67937	4234	600	21.3	0.38	55.45015	RGI60-15.04176	G085747E28200N	NA	0.149300667
**GL106_DN_3**	Himalayas	22.03.21	13:00	4	Langshisha	28.20647	85.67937	4234	600	21.3	0.38	55.45015	RGI60-15.04176	G085747E28200N	NA	0.036236
**GL107_UP_1**	Himalayas	23.03.21	09:00	0.1	Shalbachum	28.21388	85.66135	4255	71	12.96	0.45	29.08072	RGI60-15.04119	G085645E28262N	NA	0.001244667
**GL107_UP_2**	Himalayas	23.03.21	09:00	0.1	Shalbachum	28.21388	85.66135	4255	71	12.96	0.45	29.08072	RGI60-15.04119	G085645E28262N	NA	0.012450333
**GL107_UP_3**	Himalayas	23.03.21	09:00	0.1	Shalbachum	28.21388	85.66135	4255	71	12.96	0.45	29.08072	RGI60-15.04119	G085645E28262N	NA	0.006788667
**GL107_DN_1**	Himalayas	23.03.21	11:39	2.1	Shalbachum	28.20881	85.66161	4179	1252	12.96	0.44	29.22578	RGI60-15.04119	G085645E28262N	NA	0.049512333
**GL107_DN_2**	Himalayas	23.03.21	11:39	2.1	Shalbachum	28.20881	85.66161	4179	1252	12.96	0.44	29.22578	RGI60-15.04119	G085645E28262N	NA	0.02347
**GL107_DN_3**	Himalayas	23.03.21	11:39	2.1	Shalbachum	28.20881	85.66161	4179	1252	12.96	0.44	29.22578	RGI60-15.04119	G085645E28262N	NA	0.027450333
**GL108_UP_1**	Himalayas	04.04.21	09:45	1.4	Gangapurna	28.65324	84.00706	3630	45	26.18	0.49	53.06787	RGI60-15.04768	G083997E28607N	NA	0.000354
**GL108_UP_2**	Himalayas	04.04.21	09:45	1.4	Gangapurna	28.65324	84.00706	3630	45	26.18	0.49	53.06787	RGI60-15.04768	G083997E28607N	NA	0
**GL108_UP_3**	Himalayas	04.04.21	09:45	1.4	Gangapurna	28.65324	84.00706	3630	45	26.18	0.49	53.06787	RGI60-15.04768	G083997E28607N	NA	0
**GL108_DN_1**	Himalayas	04.04.21	12:24	7.2	Gangapurna	28.6595	84.01666	3508	637	26.18	0.48	54.45003	RGI60-15.04768	G083997E28607N	NA	0.002453667

**GL108_DN_2**	Himalayas	04.04.21	12:24	7.2	Gangapurna	28.6595	84.01666	3508	637	26.18	0.48	54.45003	RGI60-15.04768	G083997E28607N	NA	0.001791333
**GL108_DN_3**	Himalayas	04.04.21	12:24	7.2	Gangapurna	28.6595	84.01666	3508	637	26.18	0.48	54.45003	RGI60-15.04768	G083997E28607N	NA	0.001015667
**GL109_UP_1**	Himalayas	05.04.21	09:03	1.1	Bhakra	28.64621	84.04721	3543	146	1.25	0.21	6.000976	RGI60-15.04770	G084033E28623N	NA	0.0010355
**GL109_UP_2**	Himalayas	05.04.21	09:03	1.1	Bhakra	28.64621	84.04721	3543	146	1.25	0.21	6.000976	RGI60-15.04770	G084033E28623N	NA	0.003879667
**GL109_UP_3**	Himalayas	05.04.21	09:03	1.1	Bhakra	28.64621	84.04721	3543	146	1.25	0.21	6.000976	RGI60-15.04770	G084033E28623N	NA	0.001128667
**GL109_DN_1**	Himalayas	05.04.21	12:00	5.1	Bhakra	28.64778	84.04729	3496	496	1.25	0.2	6.160531	RGI60-15.04770	G084033E28623N	NA	0.000978333
**GL109_DN_2**	Himalayas	05.04.21	12:00	5.1	Bhakra	28.64778	84.04729	3496	496	1.25	0.2	6.160531	RGI60-15.04770	G084033E28623N	NA	0.000355
**GL109_DN_3**	Himalayas	05.04.21	12:00	5.1	Bhakra	28.64778	84.04729	3496	496	1.25	0.2	6.160531	RGI60-15.04770	G084033E28623N	NA	0.0003109
**GL110_UP_1**	Himalayas	07.04.21	11:00	0.2	Chulu West	28.74877	83.98823	4829	69	1.79	0.58	3.084533	RGI60-15.04495	G084014E28747N	NA	0.000544333
**GL110_UP_2**	Himalayas	07.04.21	11:00	0.2	Chulu West	28.74877	83.98823	4829	69	1.79	0.58	3.084533	RGI60-15.04495	G084014E28747N	NA	3.12E-05
**GL110_UP_3**	Himalayas	07.04.21	11:00	0.2	Chulu West	28.74877	83.98823	4829	69	1.79	0.58	3.084533	RGI60-15.04495	G084014E28747N	NA	0.000325667
**GL110_DN_1**	Himalayas	07.04.21	13:03	2.1	Chulu West	28.74698	83.9882	4762	640	1.79	0.57	3.156641	RGI60-15.04495	G084014E28747N	NA	0
**GL110_DN_2**	Himalayas	07.04.21	13:03	2.1	Chulu West	28.74698	83.9882	4762	640	1.79	0.57	3.156641	RGI60-15.04495	G084014E28747N	NA	0
**GL110_DN_3**	Himalayas	07.04.21	13:03	2.1	Chulu West	28.74698	83.9882	4762	640	1.79	0.57	3.156641	RGI60-15.04495	G084014E28747N	NA	0
**GL111_UP_1**	Himalayas	09.04.21	13:00	2.1	"above TP high camp"	28.77912	83.95431	5093	188	1.52	0.47	3.250183	RGI60-15.04449	G083940E28774N	NA	0.000378333
**GL111_UP_2**	Himalayas	09.04.21	13:00	2.1	"above TP high camp"	28.77912	83.95431	5093	188	1.52	0.47	3.250183	RGI60-15.04449	G083940E28774N	NA	0.000163333
**GL111_UP_3**	Himalayas	09.04.21	13:00	2.1	"above TP high camp"	28.77912	83.95431	5093	188	1.52	0.47	3.250183	RGI60-15.04449	G083940E28774N	NA	0.001254667
**GL111_DN_1**	Himalayas	09.04.21	10:30	0	"above TP high camp"	28.78457	83.96045	5003	595	1.52	0.37	4.059321	RGI60-15.04449	G083940E28774N	NA	0.006333667
**GL111_DN_2**	Himalayas	09.04.21	10:30	0	"above TP high camp"	28.78457	83.96045	5003	595	1.52	0.37	4.059321	RGI60-15.04449	G083940E28774N	NA	0.002515333

**GL111_DN_3**	Himalayas	09.04.21	10:30	0	"above TP high camp"	28.78457	83.96045	5003	595	1.52	0.37	4.059321	RGI60-15.04449	G083940E28774N	NA	0.017456
**GL112_UP_1**	Himalayas	10.04.21	10:50	3.9	Purpung Himal	28.78145	83.99191	4931	375	1.24	0.45	2.756896	RGI60-15.04748	G084004E28793N	NA	0.003057333
**GL112_UP_2**	Himalayas	10.04.21	10:50	3.9	Purpung Himal	28.78145	83.99191	4931	375	1.24	0.45	2.756896	RGI60-15.04748	G084004E28793N	NA	0.014051
**GL112_UP_3**	Himalayas	10.04.21	10:50	3.9	Purpung Himal	28.78145	83.99191	4931	375	1.24	0.45	2.756896	RGI60-15.04748	G084004E28793N	NA	0.002277667
**GL112_DN_1**	Himalayas	10.04.21	13:30	0.4	Purpung Himal	28.78178	83.98731	4856	2320	1.24	0.43	2.867726	RGI60-15.04748	G084004E28793N	NA	3.53E-05
**GL112_DN_2**	Himalayas	10.04.21	13:30	0.4	Purpung Himal	28.78178	83.98731	4856	2320	1.24	0.43	2.867726	RGI60-15.04748	G084004E28793N	NA	0.000190533
**GL112_DN_3**	Himalayas	10.04.21	13:30	0.4	Purpung Himal	28.78178	83.98731	4856	2320	1.24	0.43	2.867726	RGI60-15.04748	G084004E28793N	NA	7.8E-06
**GL113_UP_1**	Himalayas	11.04.21	10:17	0.3	"Icefall Glacier"	28.78933	83.97688	4893	189	2.13	0.58	3.661281	RGI60-15.04731	G083992E28799N	NA	0
**GL113_UP_2**	Himalayas	11.04.21	10:17	0.3	"Icefall Glacier"	28.78933	83.97688	4893	189	2.13	0.58	3.661281	RGI60-15.04731	G083992E28799N	NA	0
**GL113_UP_3**	Himalayas	11.04.21	10:17	0.3	"Icefall Glacier"	28.78933	83.97688	4893	189	2.13	0.58	3.661281	RGI60-15.04731	G083992E28799N	NA	0
**GL113_DN_1**	Himalayas	11.04.21	13:05	0.3	"Icefall Glacier"	28.78656	83.97519	4758	778	2.13	0.55	3.906737	RGI60-15.04731	G083992E28799N	NA	7.47E-05
**GL113_DN_2**	Himalayas	11.04.21	13:05	0.3	"Icefall Glacier"	28.78656	83.97519	4758	778	2.13	0.55	3.906737	RGI60-15.04731	G083992E28799N	NA	0
**GL113_DN_3**	Himalayas	11.04.21	13:05	0.3	"Icefall Glacier"	28.78656	83.97519	4758	778	2.13	0.55	3.906737	RGI60-15.04731	G083992E28799N	NA	0
**GL114_UP_1**	Himalayas	12.04.21	09:46	1.9	Chulu Northwest	28.77362	83.99507	4884	42	2.34	0.73	3.204314	RGI60-15.04760	G084014E28763N	NA	0.000432333
**GL114_UP_2**	Himalayas	12.04.21	09:46	1.9	Chulu Northwest	28.77362	83.99507	4884	42	2.34	0.73	3.204314	RGI60-15.04760	G084014E28763N	NA	4.43E-05
**GL114_UP_3**	Himalayas	12.04.21	09:46	1.9	Chulu Northwest	28.77362	83.99507	4884	42	2.34	0.73	3.204314	RGI60-15.04760	G084014E28763N	NA	0.000865
**GL114_DN_1**	Himalayas	12.04.21	12:05	2.6	Chulu Northwest	28.77479	83.99102	4864	257	2.34	0.66	3.523876	RGI60-15.04760	G084014E28763N	NA	3.55E-05
**GL114_DN_2**	Himalayas	12.04.21	12:05	2.6	Chulu Northwest	28.77479	83.99102	4864	257	2.34	0.66	3.523876	RGI60-15.04760	G084014E28763N	NA	9.24E-05
**GL114_DN_3**	Himalayas	12.04.21	12:05	2.6	Chulu Northwest	28.77479	83.99102	4864	257	2.34	0.66	3.523876	RGI60-15.04760	G084014E28763N	NA	1.63E-05

**GL115_UP_1**	Himalayas	26.04.21	10:30	0.7	Nare	27.8352	86.82957	4528	681	6.5	0.28	23.54772	RGI60-15.03572	G086868E27821N	NA	0.039261
**GL115_UP_2**	Himalayas	26.04.21	10:30	0.7	Nare	27.8352	86.82957	4528	681	6.5	0.28	23.54772	RGI60-15.03572	G086868E27821N	NA	0.088076
**GL115_UP_3**	Himalayas	26.04.21	10:30	0.7	Nare	27.8352	86.82957	4528	681	6.5	0.28	23.54772	RGI60-15.03572	G086868E27821N	NA	0.047085
**GL115_DN_1**	Himalayas	26.04.21	14:15	7.6	Nare	27.84686	86.82095	4425	706	6.5	0.24	26.56523	RGI60-15.03572	G086868E27821N	NA	0.196845333
**GL115_DN_2**	Himalayas	26.04.21	14:15	7.6	Nare	27.84686	86.82095	4425	706	6.5	0.24	26.56523	RGI60-15.03572	G086868E27821N	NA	0.202751
**GL115_DN_3**	Himalayas	26.04.21	14:15	7.6	Nare	27.84686	86.82095	4425	706	6.5	0.24	26.56523	RGI60-15.03572	G086868E27821N	NA	0.312559
**GL116_UP_1**	Himalayas	28.04.21	09:45	0.7	Lhotse	27.90124	86.87882	4815	842	10.44	0.46	22.91007	RGI60-15.03742	G086915E27927N	3463	0.059679667
**GL116_UP_2**	Himalayas	28.04.21	09:45	0.7	Lhotse	27.90124	86.87882	4815	842	10.44	0.46	22.91007	RGI60-15.03742	G086915E27927N	3463	0.011919
**GL116_UP_3**	Himalayas	28.04.21	09:45	0.7	Lhotse	27.90124	86.87882	4815	842	10.44	0.46	22.91007	RGI60-15.03742	G086915E27927N	3463	0.030139
**GL116_DN_1**	Himalayas	28.04.21	12:14	2.3	Lhotse	27.90361	86.8726	4732	1736	10.44	0.45	23.2138	RGI60-15.03742	G086915E27927N	3463	0.012565
**GL116_DN_2**	Himalayas	28.04.21	12:14	2.3	Lhotse	27.90361	86.8726	4732	1736	10.44	0.45	23.2138	RGI60-15.03742	G086915E27927N	3463	0.01666
**GL116_DN_3**	Himalayas	28.04.21	12:14	2.3	Lhotse	27.90361	86.8726	4732	1736	10.44	0.45	23.2138	RGI60-15.03742	G086915E27927N	3463	0.018477667
**GL117_UP_1**	Himalayas	29.04.21	09:30	1.5	Nuptse	27.9115	86.86557	4973	523	4.95	0.35	14.35736	RGI60-15.03735	G086868E27946N	3466	0.011039667
**GL117_UP_2**	Himalayas	29.04.21	09:30	1.5	Nuptse	27.9115	86.86557	4973	523	4.95	0.35	14.35736	RGI60-15.03735	G086868E27946N	3466	0.012797667
**GL117_UP_3**	Himalayas	29.04.21	09:30	1.5	Nuptse	27.9115	86.86557	4973	523	4.95	0.35	14.35736	RGI60-15.03735	G086868E27946N	3466	0.019824
**GL117_DN_1**	Himalayas	29.04.21	12:41	1.1	Nuptse	27.91094	86.86544	4966	2464	4.95	0.34	14.38407	RGI60-15.03735	G086868E27946N	3466	0.015428
**GL117_DN_2**	Himalayas	29.04.21	12:41	1.1	Nuptse	27.91094	86.86544	4966	2464	4.95	0.34	14.38407	RGI60-15.03735	G086868E27946N	3466	0.006176333
**GL117_DN_3**	Himalayas	29.04.21	12:41	1.1	Nuptse	27.91094	86.86544	4966	2464	4.95	0.34	14.38407	RGI60-15.03735	G086868E27946N	3466	0.00928
**GL118_UP_1**	Himalayas	30.04.21	10:00	1.1	Lhotse Nup	27.91156	86.88319	4960	910	2.84	0.26	10.89911	RGI60-15.03731	G086891E27940N	3464	0.018514667

**GL118_UP_2**	Himalayas	30.04.21	10:00	1.1	Lhotse Nup	27.91156	86.88319	4960	910	2.84	0.26	10.89911	RGI60-15.03731	G086891E27940N	3464	0.009944667
**GL118_UP_3**	Himalayas	30.04.21	10:00	1.1	Lhotse Nup	27.91156	86.88319	4960	910	2.84	0.26	10.89911	RGI60-15.03731	G086891E27940N	3464	0.005551
**GL118_DN_1**	Himalayas	30.04.21	13:08	6.4	Lhotse Nup	27.90567	86.87194	4735	1086	2.84	0.23	12.47434	RGI60-15.03731	G086891E27940N	3464	0.012404667
**GL118_DN_2**	Himalayas	30.04.21	13:08	6.4	Lhotse Nup	27.90567	86.87194	4735	1086	2.84	0.23	12.47434	RGI60-15.03731	G086891E27940N	3464	0.011356
**GL118_DN_3**	Himalayas	30.04.21	13:08	6.4	Lhotse Nup	27.90567	86.87194	4735	1086	2.84	0.23	12.47434	RGI60-15.03731	G086891E27940N	3464	0.015435
**GL119_UP_1**	Himalayas	02.05.21	09:50	4.6	Cholo	27.91355	86.80015	4391	219	1.67	0.35	4.787129	RGI60-15.03739	G086786E27909N	NA	0.287251333
**GL119_UP_2**	Himalayas	02.05.21	09:50	4.6	Cholo	27.91355	86.80015	4391	219	1.67	0.35	4.787129	RGI60-15.03739	G086786E27909N	NA	0.199292667
**GL119_UP_3**	Himalayas	02.05.21	09:50	4.6	Cholo	27.91355	86.80015	4391	219	1.67	0.35	4.787129	RGI60-15.03739	G086786E27909N	NA	0.546088333
**GL119_DN_1**	Himalayas	02.05.21	11:57	5.4	Cholo	27.9122	86.80112	4367	660	1.67	0.3	5.490192	RGI60-15.03739	G086786E27909N	NA	0.223868667
**GL119_DN_2**	Himalayas	02.05.21	11:57	5.4	Cholo	27.9122	86.80112	4367	660	1.67	0.3	5.490192	RGI60-15.03739	G086786E27909N	NA	0.922683667
**GL119_DN_3**	Himalayas	02.05.21	11:57	5.4	Cholo	27.9122	86.80112	4367	660	1.67	0.3	5.490192	RGI60-15.03739	G086786E27909N	NA	0.412089333
**GL120_UP_1**	Pamir & Tien Shan	19.07.21	08:00	0.6	Aksai	42.5326	74.5273	3323	5	6.34	0.42	14.98732	RGI60-13.11414	G074544E42510N	NA	0.003914667
**GL120_UP_2**	Pamir & Tien Shan	19.07.21	08:00	0.6	Aksai	42.5326	74.5273	3323	5	6.34	0.42	14.98732	RGI60-13.11414	G074544E42510N	NA	0.005873667
**GL120_UP_3**	Pamir & Tien Shan	19.07.21	08:00	0.6	Aksai	42.5326	74.5273	3323	5	6.34	0.42	14.98732	RGI60-13.11414	G074544E42510N	NA	0.007104667
**GL120_DN_1**	Pamir & Tien Shan	19.07.21	09:00	0.8	Aksai	42.5328	74.527	3319	42	6.34	0.42	14.99195	RGI60-13.11414	G074544E42510N	NA	0.001663333
**GL120_DN_2**	Pamir & Tien Shan	19.07.21	09:00	0.8	Aksai	42.5328	74.527	3319	42	6.34	0.42	14.99195	RGI60-13.11414	G074544E42510N	NA	0.002118667
**GL120_DN_3**	Pamir & Tien Shan	19.07.21	09:00	0.8	Aksai	42.5328	74.527	3319	42	6.34	0.42	14.99195	RGI60-13.11414	G074544E42510N	NA	0.001614
**GL121_UP_1**	Pamir & Tien Shan	20.07.21	12:49	1.7	Top Karagay	42.5056	74.5048	3334	78	4.69	0.35	13.39017	RGI60-13.11604	G074547E42486N	NA	0.001456
**GL121_UP_2**	Pamir & Tien Shan	20.07.21	12:49	1.7	Top Karagay	42.5056	74.5048	3334	78	4.69	0.35	13.39017	RGI60-13.11604	G074547E42486N	NA	0.007044667

**GL121_UP_3**	Pamir & Tien Shan	20.07.21	12:49	1.7	Top Karagay	42.5056	74.5048	3334	78	4.69	0.35	13.39017	RGI60-13.11604	G074547E42486N	NA	0.002287
**GL121_DN_1**	Pamir & Tien Shan	20.07.21	14:15	3.4	Top Karagay	42.5095	74.5014	3267	615	4.69	0.34	13.82693	RGI60-13.11604	G074547E42486N	NA	0.016487
**GL121_DN_2**	Pamir & Tien Shan	20.07.21	14:15	3.4	Top Karagay	42.5095	74.5014	3267	615	4.69	0.34	13.82693	RGI60-13.11604	G074547E42486N	NA	0.057017333
**GL121_DN_3**	Pamir & Tien Shan	20.07.21	14:15	3.4	Top Karagay	42.5095	74.5014	3267	615	4.69	0.34	13.82693	RGI60-13.11604	G074547E42486N	NA	0.036856667
**GL122_UP_1**	Pamir & Tien Shan	21.07.21	12:20	0.3	Golubin	42.4755	74.4829	3371	3	5.33	0.64	8.278834	RGI60-13.11609	G074498E42454N	753	0.001283
**GL122_UP_2**	Pamir & Tien Shan	21.07.21	12:20	0.3	Golubin	42.4755	74.4829	3371	3	5.33	0.64	8.278834	RGI60-13.11609	G074498E42454N	753	0.015938333
**GL122_UP_3**	Pamir & Tien Shan	21.07.21	12:20	0.3	Golubin	42.4755	74.4829	3371	3	5.33	0.64	8.278834	RGI60-13.11609	G074498E42454N	753	0.016486
**GL122_DN_1**	Pamir & Tien Shan	21.07.21	13:30	0.7	Golubin	42.4778	74.4816	3288	284	5.33	0.61	8.700598	RGI60-13.11609	G074498E42454N	753	0.027756667
**GL122_DN_2**	Pamir & Tien Shan	21.07.21	13:30	0.7	Golubin	42.4778	74.4816	3288	284	5.33	0.61	8.700598	RGI60-13.11609	G074498E42454N	753	0.025972667
**GL122_DN_3**	Pamir & Tien Shan	21.07.21	13:30	0.7	Golubin	42.4778	74.4816	3288	284	5.33	0.61	8.700598	RGI60-13.11609	G074498E42454N	753	0.055380333
**GL123_UP_1**	Pamir & Tien Shan	22.07.21	10:30	0.85	Tuyuk	42.473	74.512	3409	6	4.92	0.7	7.001914	RGI60-13.11606	G074530E42461N	NA	0.004229
**GL123_UP_2**	Pamir & Tien Shan	22.07.21	10:30	0.85	Tuyuk	42.473	74.512	3409	6	4.92	0.7	7.001914	RGI60-13.11606	G074530E42461N	NA	0.074928
**GL123_UP_3**	Pamir & Tien Shan	22.07.21	10:30	0.85	Tuyuk	42.473	74.512	3409	6	4.92	0.7	7.001914	RGI60-13.11606	G074530E42461N	NA	0.335348667
**GL123_DN_1**	Pamir & Tien Shan	22.07.21	12:17	7.5	Tuyuk	42.4806	74.4998	3241	1347	4.92	0.58	8.431433	RGI60-13.11606	G074530E42461N	NA	0.017113
**GL123_DN_2**	Pamir & Tien Shan	22.07.21	12:17	7.5	Tuyuk	42.4806	74.4998	3241	1347	4.92	0.58	8.431433	RGI60-13.11606	G074530E42461N	NA	0.028460667
**GL123_DN_3**	Pamir & Tien Shan	22.07.21	12:17	7.5	Tuyuk	42.4806	74.4998	3241	1347	4.92	0.58	8.431433	RGI60-13.11606	G074530E42461N	NA	0.013025667
**GL124_UP_1**	Pamir & Tien Shan	23.07.21	13:42	0.5	Tokragula	42.4383	74.4595	3514	10	1.48	0.74	2.013706	RGI60-13.11419	G074466E42432N	NA	0.099491
**GL124_UP_2**	Pamir & Tien Shan	23.07.21	13:42	0.5	Tokragula	42.4383	74.4595	3514	10	1.48	0.74	2.013706	RGI60-13.11419	G074466E42432N	NA	0.094062333
**GL124_UP_3**	Pamir & Tien Shan	23.07.21	13:42	0.5	Tokragula	42.4383	74.4595	3514	10	1.48	0.74	2.013706	RGI60-13.11419	G074466E42432N	NA	0.229163333

**GL124_DN_1**	Pamir & Tien Shan	23.07.21	16:33	6.8	Tokragula	42.4439	74.4549	3401	765	3.12	0.68	4.560607	RGI60-13.11419	G074466E42432N	NA	0.029155
**GL124_DN_2**	Pamir & Tien Shan	23.07.21	16:33	6.8	Tokragula	42.4439	74.4549	3401	765	3.12	0.68	4.560607	RGI60-13.11419	G074466E42432N	NA	0.018827333
**GL124_DN_3**	Pamir & Tien Shan	23.07.21	16:33	6.8	Tokragula	42.4439	74.4549	3401	765	3.12	0.68	4.560607	RGI60-13.11419	G074466E42432N	NA	0.017412
**GL125_UP_1**	Pamir & Tien Shan	24.07.21	11:30	0.2	Tokragula northeast	42.4399	74.4641	3593	2	1.67	0.68	2.439279	RGI60-13.11643	G074478E42443N	NA	0.017055333
**GL125_UP_2**	Pamir & Tien Shan	24.07.21	11:30	0.2	Tokragula northeast	42.4399	74.4641	3593	2	1.67	0.68	2.439279	RGI60-13.11643	G074478E42443N	NA	0.014963667
**GL125_UP_3**	Pamir & Tien Shan	24.07.21	11:30	0.2	Tokragula northeast	42.4399	74.4641	3593	2	1.67	0.68	2.439279	RGI60-13.11643	G074478E42443N	NA	0.030972
**GL125_DN_1**	Pamir & Tien Shan	24.07.21	14:30	3.1	Tokragula northeast	42.4444	74.4555	3394	940	1.73	0.52	3.342944	RGI60-13.11643	G074478E42443N	NA	0.084932333
**GL125_DN_2**	Pamir & Tien Shan	24.07.21	14:30	3.1	Tokragula northeast	42.4444	74.4555	3394	940	1.73	0.52	3.342944	RGI60-13.11643	G074478E42443N	NA	0.073832333
**GL125_DN_3**	Pamir & Tien Shan	24.07.21	14:30	3.1	Tokragula northeast	42.4444	74.4555	3394	940	1.73	0.52	3.342944	RGI60-13.11643	G074478E42443N	NA	0.056049
**GL126_UP_1**	Pamir & Tien Shan	31.07.21	11:25	0	Petrovski	39.4713	72.8967	4020	15	1.15	0.73	1.570673	RGI60-13.13094	G072890E39466N	NA	0.001614
**GL126_UP_2**	Pamir & Tien Shan	31.07.21	11:25	0	Petrovski	39.4713	72.8967	4020	15	1.15	0.73	1.570673	RGI60-13.13094	G072890E39466N	NA	0.007047
**GL126_UP_3**	Pamir & Tien Shan	31.07.21	11:25	0	Petrovski	39.4713	72.8967	4020	15	1.15	0.73	1.570673	RGI60-13.13094	G072890E39466N	NA	0.001697
**GL126_DN_1**	Pamir & Tien Shan	31.07.21	15:00	4	Petrovski	39.4818	72.8957	3824	1280	1.15	0.38	3.005052	RGI60-13.13094	G072890E39466N	NA	0.003406667
**GL126_DN_2**	Pamir & Tien Shan	31.07.21	15:00	4	Petrovski	39.4818	72.8957	3824	1280	1.15	0.38	3.005052	RGI60-13.13094	G072890E39466N	NA	0.001350333
**GL126_DN_3**	Pamir & Tien Shan	31.07.21	15:00	4	Petrovski	39.4818	72.8957	3824	1280	1.15	0.38	3.005052	RGI60-13.13094	G072890E39466N	NA	0.007343667
**GL127_UP_1**	Pamir & Tien Shan	01.08.21	11:45	0.25	North of Lenin	39.4419	72.8983	3970	1192	1.03	0.26	4.031847	RGI60-13.13098	G072884E39433N	NA	0.041700333
**GL127_UP_2**	Pamir & Tien Shan	01.08.21	11:45	0.25	North of Lenin	39.4419	72.8983	3970	1192	1.03	0.26	4.031847	RGI60-13.13098	G072884E39433N	NA	0.030052667
**GL127_UP_3**	Pamir & Tien Shan	01.08.21	11:45	0.25	North of Lenin	39.4419	72.8983	3970	1192	1.03	0.26	4.031847	RGI60-13.13098	G072884E39433N	NA	0.022966667
**GL127_DN_1**	Pamir & Tien Shan	01.08.21	14:15	4.4	North of Lenin	39.4461	72.9048	3901	1947	1.03	0.22	4.715211	RGI60-13.13098	G072884E39433N	NA	0

**GL127_DN_2**	Pamir & Tien Shan	01.08.21	14:15	4.4	North of Lenin	39.4461	72.9048	3901	1947	1.03	0.22	4.715211	RGI60-13.13098	G072884E39433N	NA	0
**GL127_DN_3**	Pamir & Tien Shan	01.08.21	14:15	4.4	North of Lenin	39.4461	72.9048	3901	1947	1.03	0.22	4.715211	RGI60-13.13098	G072884E39433N	NA	0
**GL128_UP_1**	Pamir & Tien Shan	02.08.21	11:30	1.2	No. 197a	39.4424	72.9367	3878	186	7.59	0.56	13.44629	RGI60-13.13252	G072967E39424N	NA	0.000496
**GL128_UP_2**	Pamir & Tien Shan	02.08.21	11:30	1.2	No. 197a	39.4424	72.9367	3878	186	7.59	0.56	13.44629	RGI60-13.13252	G072967E39424N	NA	0
**GL128_UP_3**	Pamir & Tien Shan	02.08.21	11:30	1.2	No. 197a	39.4424	72.9367	3878	186	7.59	0.56	13.44629	RGI60-13.13252	G072967E39424N	NA	0
**GL128_DN_1**	Pamir & Tien Shan	02.08.21	14:00	2.9	No. 197a	39.4459	72.9292	3777	1078	8.89	0.56	15.92118	RGI60-13.13252	G072967E39424N	NA	0
**GL128_DN_2**	Pamir & Tien Shan	02.08.21	14:00	2.9	No. 197a	39.4459	72.9292	3777	1078	8.89	0.56	15.92118	RGI60-13.13252	G072967E39424N	NA	0.000106333
**GL128_DN_3**	Pamir & Tien Shan	02.08.21	14:00	2.9	No. 197a	39.4459	72.9292	3777	1078	8.89	0.56	15.92118	RGI60-13.13252	G072967E39424N	NA	0
**GL129_UP_1**	Pamir & Tien Shan	04.08.21	09:30	0.4	"No. 199a Lenin"	39.4441	72.9168	3764	539	19.43	0.7	27.72235	RGI60-13.13251	G072928E39397N	NA	0
**GL129_UP_2**	Pamir & Tien Shan	04.08.21	09:30	0.4	"No. 199a Lenin"	39.4441	72.9168	3764	539	19.43	0.7	27.72235	RGI60-13.13251	G072928E39397N	NA	0
**GL129_UP_3**	Pamir & Tien Shan	04.08.21	09:30	0.4	"No. 199a Lenin"	39.4441	72.9168	3764	539	19.43	0.7	27.72235	RGI60-13.13251	G072928E39397N	NA	0
**GL129_DN_1**	Pamir & Tien Shan	04.08.21	11:50	0.9	"No. 199a Lenin"	39.4498	72.9135	3733	1255	55.46	0.69	79.79871	RGI60-13.13251	G072928E39397N	NA	0
**GL129_DN_2**	Pamir & Tien Shan	04.08.21	11:50	0.9	"No. 199a Lenin"	39.4498	72.9135	3733	1255	55.46	0.69	79.79871	RGI60-13.13251	G072928E39397N	NA	0
**GL129_DN_3**	Pamir & Tien Shan	04.08.21	11:50	0.9	"No. 199a Lenin"	39.4498	72.9135	3733	1255	55.46	0.69	79.79871	RGI60-13.13251	G072928E39397N	NA	0
**GL130_UP_1**	Pamir & Tien Shan	11.08.21	11:05	4.7	Bordu	41.8245	78.1545	3838	41	5.1	0.7	7.292166	RGI60-13.08054	G078175E41813N	829	0.035548333
**GL130_UP_2**	Pamir & Tien Shan	11.08.21	11:05	4.7	Bordu	41.8245	78.1545	3838	41	5.1	0.7	7.292166	RGI60-13.08054	G078175E41813N	829	0.010590333
**GL130_UP_3**	Pamir & Tien Shan	11.08.21	11:05	4.7	Bordu	41.8245	78.1545	3838	41	5.1	0.7	7.292166	RGI60-13.08054	G078175E41813N	829	0.114911333
**GL130_DN_1**	Pamir & Tien Shan	11.08.21	14:23	3	Bordu	41.8313	78.1378	3721	1644	5.11	0.53	9.627045	RGI60-13.08054	G078175E41813N	829	0.003843
**GL130_DN_2**	Pamir & Tien Shan	11.08.21	14:23	3	Bordu	41.8313	78.1378	3721	1644	5.11	0.53	9.627045	RGI60-13.08054	G078175E41813N	829	0.044563667

**GL130_DN_3**	Pamir & Tien Shan	11.08.21	14:23	3	Bordu	41.8313	78.1378	3721	1644	5.11	0.53	9.627045	RGI60-13.08054	G078175E41813N	829	0.013144333
**GL131_UP_1**	Pamir & Tien Shan	12.08.21	11:15	1.1	No. 354	41.8091	78.1402	3813	101	6.55	0.62	10.58635	RGI60-13.07064	G078164E41793N	3889	0.164037
**GL131_UP_2**	Pamir & Tien Shan	12.08.21	11:15	1.1	No. 354	41.8091	78.1402	3813	101	6.55	0.62	10.58635	RGI60-13.07064	G078164E41793N	3889	0.313821667
**GL131_UP_3**	Pamir & Tien Shan	12.08.21	11:15	1.1	No. 354	41.8091	78.1402	3813	101	6.55	0.62	10.58635	RGI60-13.07064	G078164E41793N	3889	0.228770667
**GL131_DN_1**	Pamir & Tien Shan	12.08.21	14:15	1.5	No. 354	41.811	78.1244	3686	1484	6.55	0.55	11.93788	RGI60-13.07064	G078164E41793N	3889	0.173064667
**GL131_DN_2**	Pamir & Tien Shan	12.08.21	14:15	1.5	No. 354	41.811	78.1244	3686	1484	6.55	0.55	11.93788	RGI60-13.07064	G078164E41793N	3889	0.012503667
**GL131_DN_3**	Pamir & Tien Shan	12.08.21	14:15	1.5	No. 354	41.811	78.1244	3686	1484	6.55	0.55	11.93788	RGI60-13.07064	G078164E41793N	3889	0.075638667
**GL132_UP_1**	Pamir & Tien Shan	13.08.21	11:31	1.5	"Southwest of 354"	41.8009	78.1335	3869	97	1.83	0.57	3.190431	RGI60-13.07137	G078141E41787N	NA	0.099369667
**GL132_UP_2**	Pamir & Tien Shan	13.08.21	11:31	1.5	"Southwest of 354"	41.8009	78.1335	3869	97	1.83	0.57	3.190431	RGI60-13.07137	G078141E41787N	NA	0.102625333
**GL132_UP_3**	Pamir & Tien Shan	13.08.21	11:31	1.5	"Southwest of 354"	41.8009	78.1335	3869	97	1.83	0.57	3.190431	RGI60-13.07137	G078141E41787N	NA	0.067785
**GL132_DN_1**	Pamir & Tien Shan	13.08.21	14:15	2.5	"Southwest of 354"	41.8034	78.1293	3822	560	1.83	0.5	3.628939	RGI60-13.07137	G078141E41787N	NA	0.238129333
**GL132_DN_2**	Pamir & Tien Shan	13.08.21	14:15	2.5	"Southwest of 354"	41.8034	78.1293	3822	560	1.83	0.5	3.628939	RGI60-13.07137	G078141E41787N	NA	0.115881333
**GL132_DN_3**	Pamir & Tien Shan	13.08.21	14:15	2.5	"Southwest of 354"	41.8034	78.1293	3822	560	1.83	0.5	3.628939	RGI60-13.07137	G078141E41787N	NA	0.339004333
**GL133_UP_1**	Pamir & Tien Shan	14.08.21	11:00	0.1	"Road 1"	41.8879	77.6879	3899	1	0.52	0.69	0.746546	RGI60-13.06829	G077684E41883N	NA	2.392561667
**GL133_UP_2**	Pamir & Tien Shan	14.08.21	11:00	0.1	"Road 1"	41.8879	77.6879	3899	1	0.52	0.69	0.746546	RGI60-13.06829	G077684E41883N	NA	1.289180667
**GL133_UP_3**	Pamir & Tien Shan	14.08.21	11:00	0.1	"Road 1"	41.8879	77.6879	3899	1	0.52	0.69	0.746546	RGI60-13.06829	G077684E41883N	NA	1.225635
**GL133_DN_1**	Pamir & Tien Shan	14.08.21	14:00	4	"Road 1"	41.8901	77.6894	3796	278	0.52	0.59	0.883257	RGI60-13.06829	G077684E41883N	NA	0.384059
**GL133_DN_2**	Pamir & Tien Shan	14.08.21	14:00	4	"Road 1"	41.8901	77.6894	3796	278	0.52	0.59	0.883257	RGI60-13.06829	G077684E41883N	NA	0.455440333
**GL133_DN_3**	Pamir & Tien Shan	14.08.21	14:00	4	"Road 1"	41.8901	77.6894	3796	278	0.52	0.59	0.883257	RGI60-13.06829	G077684E41883N	NA	0.186902333

**GL134_UP_1**	Pamir & Tien Shan	16.08.21	10:35	0	"North of road"	41.9099	77.715	4041	0	1.01	0.58	1.726232	RGI60-13.07981	G077719E41916N	NA	0.239702667
**GL134_UP_2**	Pamir & Tien Shan	16.08.21	10:35	0	"North of road"	41.9099	77.715	4041	0	1.01	0.58	1.726232	RGI60-13.07981	G077719E41916N	NA	0.661688333
**GL134_UP_3**	Pamir & Tien Shan	16.08.21	10:35	0	"North of road"	41.9099	77.715	4041	0	1.01	0.58	1.726232	RGI60-13.07981	G077719E41916N	NA	0.479725667
**GL134_DN_1**	Pamir & Tien Shan	16.08.21	13:00	1	"North of road"	41.9076	77.7135	3965	283	1.03	0.44	2.35	RGI60-13.07981	G077719E41916N	NA	0.098721667
**GL134_DN_2**	Pamir & Tien Shan	16.08.21	13:00	1	"North of road"	41.9076	77.7135	3965	283	1.03	0.44	2.35	RGI60-13.07981	G077719E41916N	NA	0.025398333
**GL134_DN_3**	Pamir & Tien Shan	16.08.21	13:00	1	"North of road"	41.9076	77.7135	3965	283	1.03	0.44	2.35	RGI60-13.07981	G077719E41916N	NA	0.073909333
**GL135_UP_1**	Pamir & Tien Shan	17.08.21	13:46	0.4	West Suek/ Batysh Sook	41.7985	77.7495	3925	9	0.99	0.63	1.56763	RGI60-13.06974	G077749E41787N	781	1.035258667
**GL135_UP_2**	Pamir & Tien Shan	17.08.21	13:46	0.4	West Suek/ Batysh Sook	41.7985	77.7495	3925	9	0.99	0.63	1.56763	RGI60-13.06974	G077749E41787N	781	0.437503667
**GL135_UP_3**	Pamir & Tien Shan	17.08.21	13:46	0.4	West Suek/ Batysh Sook	41.7985	77.7495	3925	9	0.99	0.63	1.56763	RGI60-13.06974	G077749E41787N	781	0.455414
**GL135_DN_1**	Pamir & Tien Shan	17.08.21	15:45	0.6	West Suek/ Batysh Sook	41.7995	77.75	3900	133	0.99	0.58	1.701388	RGI60-13.06974	G077749E41787N	781	1.122175
**GL135_DN_2**	Pamir & Tien Shan	17.08.21	15:45	0.6	West Suek/ Batysh Sook	41.7995	77.75	3900	133	0.99	0.58	1.701388	RGI60-13.06974	G077749E41787N	781	0.836211667
**GL135_DN_3**	Pamir & Tien Shan	17.08.21	15:45	0.6	West Suek/ Batysh Sook	41.7995	77.75	3900	133	0.99	0.58	1.701388	RGI60-13.06974	G077749E41787N	781	0.366038
**GL136_UP_1**	Pamir & Tien Shan	18.08.21	10:07	0	"2nd west of West Suek"	41.7929	77.7189	3950	4	1.46	0.53	2.767202	RGI60-13.06972	G077722E41781N	NA	0.167194667
**GL136_UP_2**	Pamir & Tien Shan	18.08.21	10:07	0	"2nd west of West Suek"	41.7929	77.7189	3950	4	1.46	0.53	2.767202	RGI60-13.06972	G077722E41781N	NA	0.108157
**GL136_UP_3**	Pamir & Tien Shan	18.08.21	10:07	0	"2nd west of West Suek"	41.7929	77.7189	3950	4	1.46	0.53	2.767202	RGI60-13.06972	G077722E41781N	NA	0.117958333
**GL136_DN_1**	Pamir & Tien Shan	18.08.21	12:40	0.2	"2nd west of West Suek"	41.7943	77.7181	3926	169	1.46	0.5	2.94	RGI60-13.06972	G077722E41781N	NA	0.235712333
**GL136_DN_2**	Pamir & Tien Shan	18.08.21	12:40	0.2	"2nd west of West Suek"	41.7943	77.7181	3926	169	1.46	0.5	2.94	RGI60-13.06972	G077722E41781N	NA	0.473734
**GL136_DN_3**	Pamir & Tien Shan	18.08.21	12:40	0.2	"2nd west of West Suek"	41.7943	77.7181	3926	169	1.46	0.5	2.94	RGI60-13.06972	G077722E41781N	NA	0.473645667
**GL137_UP_1**	Pamir & Tien Shan	19.08.21	11:20	0.2	"West of West Suek"	41.7925	77.7353	3958	2	0.88	0.42	2.089553	RGI60-13.06973	G077735E41783N	NA	0.044339667

**GL137_UP_2**	Pamir & Tien Shan	19.08.21	11:20	0.2	"West of West Suek"	41.7925	77.7353	3958	2	0.88	0.42	2.089553	RGI60-13.06973	G077735E41783N	NA	0.019687667
**GL137_UP_3**	Pamir & Tien Shan	19.08.21	11:20	0.2	"West of West Suek"	41.7925	77.7353	3958	2	0.88	0.42	2.089553	RGI60-13.06973	G077735E41783N	NA	0.059792
**GL137_DN_1**	Pamir & Tien Shan	19.08.21	14:43	3	"West of West Suek"	41.8019	77.7369	3874	1060	0.88	0.25	3.54	RGI60-13.06973	G077735E41783N	NA	0.867560333
**GL137_DN_2**	Pamir & Tien Shan	19.08.21	14:43	3	"West of West Suek"	41.8019	77.7369	3874	1060	0.88	0.25	3.54	RGI60-13.06973	G077735E41783N	NA	2.145108667
**GL137_DN_3**	Pamir & Tien Shan	19.08.21	14:43	3	"West of West Suek"	41.8019	77.7369	3874	1060	0.88	0.25	3.54	RGI60-13.06973	G077735E41783N	NA	1.116446333
**GL138_UP_1**	Pamir & Tien Shan	20.08.21	10:15	0	East Suek	41.7878	77.7697	4007	2	0.42	0.41	1.024245	RGI60-13.06975	G077771E41782N	NA	0.710286667
**GL138_UP_2**	Pamir & Tien Shan	20.08.21	10:15	0	East Suek	41.7878	77.7697	4007	2	0.42	0.41	1.024245	RGI60-13.06975	G077771E41782N	NA	0.768329333
**GL138_UP_3**	Pamir & Tien Shan	20.08.21	10:15	0	East Suek	41.7878	77.7697	4007	2	0.42	0.41	1.024245	RGI60-13.06975	G077771E41782N	NA	1.753443333
**GL138_DN_1**	Pamir & Tien Shan	20.08.21	13:05	0.8	East Suek	41.7926	77.7672	3909	586	0.42	0.26	1.61	RGI60-13.06975	G077771E41782N	NA	0.093014
**GL138_DN_2**	Pamir & Tien Shan	20.08.21	13:05	0.8	East Suek	41.7926	77.7672	3909	586	0.42	0.26	1.61	RGI60-13.06975	G077771E41782N	NA	0.038396333
**GL138_DN_3**	Pamir & Tien Shan	20.08.21	13:05	0.8	East Suek	41.7926	77.7672	3909	586	0.42	0.26	1.61	RGI60-13.06975	G077771E41782N	NA	0.051638333
**GL139_UP_1**	Pamir & Tien Shan	21.08.21	11:00	1.9	"Ski slope"	41.8182	77.7974	4014	59	0.14	0.78	0.17303	RGI60-13.06980	G077800E41816N	NA	0.193144
**GL139_UP_2**	Pamir & Tien Shan	21.08.21	11:00	1.9	"Ski slope"	41.8182	77.7974	4014	59	0.14	0.78	0.17303	RGI60-13.06980	G077800E41816N	NA	0.124498667
**GL139_UP_3**	Pamir & Tien Shan	21.08.21	11:00	1.9	"Ski slope"	41.8182	77.7974	4014	59	0.14	0.78	0.17303	RGI60-13.06980	G077800E41816N	NA	0.155599333
**GL139_DN_1**	Pamir & Tien Shan	21.08.21	13:55	11.5	"Ski slope"	41.8245	77.7927	3813	887	0.14	0.35	0.39	RGI60-13.06980	G077800E41816N	NA	0.025215
**GL139_DN_2**	Pamir & Tien Shan	21.08.21	13:55	11.5	"Ski slope"	41.8245	77.7927	3813	887	0.14	0.35	0.39	RGI60-13.06980	G077800E41816N	NA	0.028071667
**GL139_DN_3**	Pamir & Tien Shan	21.08.21	13:55	11.5	"Ski slope"	41.8245	77.7927	3813	887	0.14	0.35	0.39	RGI60-13.06980	G077800E41816N	NA	0.036034
**GL140_UP_1**	Rwenzori Mountains	03.12.21	11:36	0.4	Stanley 003	0.3758	29.8785	4724	43	0.03	0.91	0.04	RGI50-16.01631	G029875E00377N	NA	0.540149667
**GL140_UP_2**	Rwenzori Mountains	03.12.21	11:36	0.4	Stanley 003	0.3758	29.8785	4724	43	0.03	0.91	0.04	RGI50-16.01631	G029875E00377N	NA	0.374533

**GL140_UP_3**	Rwenzori Mountains	03.12.21	11:36	0.4	Stanley 003	0.3758	29.8785	4724	43	0.03	0.91	0.04	RGI50-16.01631	G029875E00377N	NA	0.231231667
**GL140_DN_1**	Rwenzori Mountains	03.12.21	13:00	0.8	Stanley 003	0.3755	29.8788	4722	86	0.03	0.91	0.04	RGI50-16.01631	G029875E00377N	NA	1.611483
**GL140_DN_2**	Rwenzori Mountains	03.12.21	13:00	0.8	Stanley 003	0.3755	29.8788	4722	86	0.03	0.91	0.04	RGI50-16.01631	G029875E00377N	NA	1.620655
**GL140_DN_3**	Rwenzori Mountains	03.12.21	13:00	0.8	Stanley 003	0.3755	29.8788	4722	86	0.03	0.91	0.04	RGI50-16.01631	G029875E00377N	NA	1.670730333
**GL141_UP_1**	Chilean Andes	25.01.22	12:27	3	Ventisquiero Yelcho	-43.2891	-72.4689	378	563	4.46	0.64	6.96	RGI60-17.11003	G287495E43295S	NA	0.001099
**GL141_UP_2**	Chilean Andes	25.01.22	12:27	3	Ventisquiero Yelcho	-43.2891	-72.4689	378	563	4.46	0.64	6.96	RGI60-17.11003	G287495E43295S	NA	0.005604333
**GL141_UP_3**	Chilean Andes	25.01.22	12:27	3	Ventisquiero Yelcho	-43.2891	-72.4689	378	563	4.46	0.64	6.96	RGI60-17.11003	G287495E43295S	NA	0.006102
**GL141_DN_1**	Chilean Andes	25.01.22	16:06	4.5	Ventisquiero Yelcho	-43.2863	-72.4634	312	1165	8.85	0.55	16.22	RGI60-17.11003	G287495E43295S	NA	0.006117333
**GL141_DN_2**	Chilean Andes	25.01.22	16:06	4.5	Ventisquiero Yelcho	-43.2863	-72.4634	312	1165	8.85	0.55	16.22	RGI60-17.11003	G287495E43295S	NA	0.004674333
**GL141_DN_3**	Chilean Andes	25.01.22	16:06	4.5	Ventisquiero Yelcho	-43.2863	-72.4634	312	1165	8.85	0.55	16.22	RGI60-17.11003	G287495E43295S	NA	0.003198
**GL142_UP_1**	Chilean Andes	26.01.22	13:00	6.6	Amarillo	-42.8641	-72.4453	498	493	13.22	0.76	17.44	RGI60-17.11392	G287544E42827S	4462	0.000113667
**GL142_UP_2**	Chilean Andes	26.01.22	13:00	6.6	Amarillo	-42.8641	-72.4453	498	493	13.22	0.76	17.44	RGI60-17.11392	G287544E42827S	4462	0
**GL142_UP_3**	Chilean Andes	26.01.22	13:00	6.6	Amarillo	-42.8641	-72.4453	498	493	13.22	0.76	17.44	RGI60-17.11392	G287544E42827S	4462	0
**GL142_DN_1**	Chilean Andes	26.01.22	16:15	7.5	Amarillo	-42.8825	-72.4498	430	2676	13.22	0.63	21	RGI60-17.11392	G287544E42827S	4462	0.000654
**GL142_DN_2**	Chilean Andes	26.01.22	16:15	7.5	Amarillo	-42.8825	-72.4498	430	2676	13.22	0.63	21	RGI60-17.11392	G287544E42827S	4462	0.000937
**GL142_DN_3**	Chilean Andes	26.01.22	16:15	7.5	Amarillo	-42.8825	-72.4498	430	2676	13.22	0.63	21	RGI60-17.11392	G287544E42827S	4462	0.000610667
**GL143_UP_1**	Chilean Andes	30.01.22	10:23	0	"Mocho 1 middle"	-39.9282	-72.0061	1876	3	0.1	0.97	0.1	RGI60-17.12442	G287988E39929S	NA	0.000156333
**GL143_UP_2**	Chilean Andes	30.01.22	10:23	0	"Mocho 1 middle"	-39.9282	-72.0061	1876	3	0.1	0.97	0.1	RGI60-17.12442	G287988E39929S	NA	0.000290333
**GL143_UP_3**	Chilean Andes	30.01.22	10:23	0	"Mocho 1 middle"	-39.9282	-72.0061	1876	3	0.1	0.97	0.1	RGI60-17.12442	G287988E39929S	NA	0.000279333

**GL143_DN_1**	Chilean Andes	30.01.22	13:50	6.7	"Mocho 1 middle"	-39.9261	-72	1740	616	0.1	0.58	0.17	RGI60-17.12442	G287988E39929S	NA	0.003037667
**GL143_DN_2**	Chilean Andes	30.01.22	13:50	6.7	"Mocho 1 middle"	-39.9261	-72	1740	616	0.1	0.58	0.17	RGI60-17.12442	G287988E39929S	NA	0.001444
**GL143_DN_3**	Chilean Andes	30.01.22	13:50	6.7	"Mocho 1 middle"	-39.9261	-72	1740	616	0.1	0.58	0.17	RGI60-17.12442	G287988E39929S	NA	0.001137667
**GL144_UP_1**	Chilean Andes	31.01.22	10:32	1.5	"Mocho 2 south"	-39.9305	-72.0029	1825	30	0.06	0.88	0.06	RGI60-17.12442	G287988E39929S	NA	0.000143867
**GL144_UP_2**	Chilean Andes	31.01.22	10:32	1.5	"Mocho 2 south"	-39.9305	-72.0029	1825	30	0.06	0.88	0.06	RGI60-17.12442	G287988E39929S	NA	2.43E-05
**GL144_UP_3**	Chilean Andes	31.01.22	10:32	1.5	"Mocho 2 south"	-39.9305	-72.0029	1825	30	0.06	0.88	0.06	RGI60-17.12442	G287988E39929S	NA	9.31E-05
**GL144_DN_1**	Chilean Andes	31.01.22	12:55	6.2	"Mocho 2 south"	-39.9288	-71.9997	1738	383	0.08	0.51	0.15	RGI60-17.12442	G287988E39929S	NA	0.000347
**GL144_DN_2**	Chilean Andes	31.01.22	12:55	6.2	"Mocho 2 south"	-39.9288	-71.9997	1738	383	0.08	0.51	0.15	RGI60-17.12442	G287988E39929S	NA	0.000418667
**GL144_DN_3**	Chilean Andes	31.01.22	12:55	6.2	"Mocho 2 south"	-39.9288	-71.9997	1738	383	0.08	0.51	0.15	RGI60-17.12442	G287988E39929S	NA	0.0001897
**GL145_UP_1**	Chilean Andes	01.02.22	10:30	0.7	"Mocho 3 north"	-39.9252	-72.0079	1854	22	0.16	0.84	0.19	RGI60-17.12442	G287988E39929S	NA	1.73E-05
**GL145_UP_2**	Chilean Andes	01.02.22	10:30	0.7	"Mocho 3 north"	-39.9252	-72.0079	1854	22	0.16	0.84	0.19	RGI60-17.12442	G287988E39929S	NA	1.34E-05
**GL145_UP_3**	Chilean Andes	01.02.22	10:30	0.7	"Mocho 3 north"	-39.9252	-72.0079	1854	22	0.16	0.84	0.19	RGI60-17.12442	G287988E39929S	NA	7.6E-05
**GL145_DN_1**	Chilean Andes	01.02.22	14:14	11.1	"Mocho 3 north"	-39.9244	-72.0064	1775	184	0.16	0.77	0.21	RGI60-17.12442	G287988E39929S	NA	0.000835
**GL145_DN_2**	Chilean Andes	01.02.22	14:14	11.1	"Mocho 3 north"	-39.9244	-72.0064	1775	184	0.16	0.77	0.21	RGI60-17.12442	G287988E39929S	NA	0.000769
**GL145_DN_3**	Chilean Andes	01.02.22	14:14	11.1	"Mocho 3 north"	-39.9244	-72.0064	1775	184	0.16	0.77	0.21	RGI60-17.12442	G287988E39929S	NA	0.000469667
**GL146_UP_1**	Chilean Andes	04.02.22	12:22	3.1	Pichillancahue	-39.4407	-71.8884	1817	25	0.05	0.99	0.05	RGI60-17.12517	G288107E39448S	NA	0.000707333
**GL146_UP_2**	Chilean Andes	04.02.22	12:22	3.1	Pichillancahue	-39.4407	-71.8884	1817	25	0.05	0.99	0.05	RGI60-17.12517	G288107E39448S	NA	9E-05
**GL146_UP_3**	Chilean Andes	04.02.22	12:22	3.1	Pichillancahue	-39.4407	-71.8884	1817	25	0.05	0.99	0.05	RGI60-17.12517	G288107E39448S	NA	1.4E-05
**GL146_DN_1**	Chilean Andes	04.02.22	14:45	6.1	Pichillancahue	-39.4394	-71.879	1740	905	0.22	0.62	0.35	RGI60-17.12517	G288107E39448S	NA	9.5E-05

**GL146_DN_2**	Chilean Andes	04.02.22	14:45	6.1	Pichillancahue	-39.4394	-71.879	1740	905	0.22	0.62	0.35	RGI60-17.12517	G288107E39448S	NA	0
**GL146_DN_3**	Chilean Andes	04.02.22	14:45	6.1	Pichillancahue	-39.4394	-71.879	1740	905	0.22	0.62	0.35	RGI60-17.12517	G288107E39448S	NA	0.000197667
**GL147_UP_1**	Chilean Andes	05.02.22	12:00	0	Turbio	-39.4315	-71.8797	1748	6	0.22	1	0.23	RGI60-17.12535	G288088E39427S	4413	0
**GL147_UP_2**	Chilean Andes	05.02.22	12:00	0	Turbio	-39.4315	-71.8797	1748	6	0.22	1	0.23	RGI60-17.12535	G288088E39427S	4413	0
**GL147_UP_3**	Chilean Andes	05.02.22	12:00	0	Turbio	-39.4315	-71.8797	1748	6	0.22	1	0.23	RGI60-17.12535	G288088E39427S	4413	0
**GL147_DN_1**	Chilean Andes	05.02.22	15:30	1.7	Turbio	-39.4271	-71.8786	1703	521	0.39	0.31	1.26	RGI60-17.12535	G288088E39427S	4413	0
**GL147_DN_2**	Chilean Andes	05.02.22	15:30	1.7	Turbio	-39.4271	-71.8786	1703	521	0.39	0.31	1.26	RGI60-17.12535	G288088E39427S	4413	0
**GL147_DN_3**	Chilean Andes	05.02.22	15:30	1.7	Turbio	-39.4271	-71.8786	1703	521	0.39	0.31	1.26	RGI60-17.12535	G288088E39427S	4413	6.43E-05
**GL148_UP_1**	Chilean Andes	08.02.22	11:45	0.2	"Nevado Chillan 1 east"	-36.8385	-71.4148	2734	35	0.22	0.43	0.5	RGI60-17.13045	G288585E36836S	NA	0
**GL148_UP_2**	Chilean Andes	08.02.22	11:45	0.2	"Nevado Chillan 1 east"	-36.8385	-71.4148	2734	35	0.22	0.43	0.5	RGI60-17.13045	G288585E36836S	NA	0
**GL148_UP_3**	Chilean Andes	08.02.22	11:45	0.2	"Nevado Chillan 1 east"	-36.8385	-71.4148	2734	35	0.22	0.43	0.5	RGI60-17.13045	G288585E36836S	NA	0
**GL148_DN_1**	Chilean Andes	08.02.22	14:15	3.6	"Nevado Chillan 1 east"	-36.8405	-71.4166	2610	312	0.22	0.41	0.53	RGI60-17.13045	G288585E36836S	NA	0
**GL148_DN_2**	Chilean Andes	08.02.22	14:15	3.6	"Nevado Chillan 1 east"	-36.8405	-71.4166	2610	312	0.22	0.41	0.53	RGI60-17.13045	G288585E36836S	NA	0
**GL148_DN_3**	Chilean Andes	08.02.22	14:15	3.6	"Nevado Chillan 1 east"	-36.8405	-71.4166	2610	312	0.22	0.41	0.53	RGI60-17.13045	G288585E36836S	NA	0
**GL149_UP_1**	Chilean Andes	09.02.22	09:30	2.8	"Nevado Chillan 2 west"	-36.8451	-71.4253	2273	1034	0.16	0.21	0.75	RGI60-17.13045	G288585E36836S	NA	0
**GL149_UP_2**	Chilean Andes	09.02.22	09:30	2.8	"Nevado Chillan 2 west"	-36.8451	-71.4253	2273	1034	0.16	0.21	0.75	RGI60-17.13045	G288585E36836S	NA	0
**GL149_UP_3**	Chilean Andes	09.02.22	09:30	2.8	"Nevado Chillan 2 west"	-36.8451	-71.4253	2273	1034	0.16	0.21	0.75	RGI60-17.13045	G288585E36836S	NA	0
**GL149_DN_1**	Chilean Andes	09.02.22	12:05	8.6	"Nevado Chillan 2 west"	-36.8487	-71.4323	2176	1852	0.16	0.11	1.45	RGI60-17.13045	G288585E36836S	NA	4.1E-06
**GL149_DN_2**	Chilean Andes	09.02.22	12:05	8.6	"Nevado Chillan 2 west"	-36.8487	-71.4323	2176	1852	0.16	0.11	1.45	RGI60-17.13045	G288585E36836S	NA	0

**GL149_DN_3**	Chilean Andes	09.02.22	12:05	8.6	"Nevado Chillan 2 west"	-36.8487	-71.4323	2176	1852	0.16	0.11	1.45	RGI60-17.13045	G288585E36836S	NA	0
**GL150_UP_1**	Chilean Andes	11.02.22	09:22	0.1	Universidad	-34.7116	-70.3437	2524	30	25.63	0.52	48.85	RGI60-17.01218	G289664E34607S	2009	0.00039
**GL150_UP_2**	Chilean Andes	11.02.22	09:22	0.1	Universidad	-34.7116	-70.3437	2524	30	25.63	0.52	48.85	RGI60-17.01218	G289664E34607S	2009	2.03E-06
**GL150_UP_3**	Chilean Andes	11.02.22	09:22	0.1	Universidad	-34.7116	-70.3437	2524	30	25.63	0.52	48.85	RGI60-17.01218	G289664E34607S	2009	0
**GL150_DN_1**	Chilean Andes	11.02.22	12:30	6.4	Universidad	-34.7204	-70.3592	2417	2063	30.96	0.33	94.24	RGI60-17.01218	G289664E34607S	2009	0.010316667
**GL150_DN_2**	Chilean Andes	11.02.22	12:30	6.4	Universidad	-34.7204	-70.3592	2417	2063	30.96	0.33	94.24	RGI60-17.01218	G289664E34607S	2009	0.006671333
**GL150_DN_3**	Chilean Andes	11.02.22	12:30	6.4	Universidad	-34.7204	-70.3592	2417	2063	30.96	0.33	94.24	RGI60-17.01218	G289664E34607S	2009	0.003624667
**GL151_UP_1**	Chilean Andes	17.02.22	12:45	0.5	"El Morado"	-33.736	-70.0439	3227	58	9.29	0.4	23.19	RGI60-17.13710	G289967E33714S	NA	0
**GL151_UP_2**	Chilean Andes	17.02.22	12:45	0.5	"El Morado"	-33.736	-70.0439	3227	58	9.29	0.4	23.19	RGI60-17.13710	G289967E33714S	NA	0
**GL151_UP_3**	Chilean Andes	17.02.22	12:45	0.5	"El Morado"	-33.736	-70.0439	3227	58	9.29	0.4	23.19	RGI60-17.13710	G289967E33714S	NA	0
**GL151_DN_1**	Chilean Andes	17.02.22	16:50	5.4	"El Morado"	-33.7691	-70.0441	2731	3994	10.92	0.26	42.51	RGI60-17.13710	G289967E33714S	NA	0
**GL151_DN_2**	Chilean Andes	17.02.22	16:50	5.4	"El Morado"	-33.7691	-70.0441	2731	3994	10.92	0.26	42.51	RGI60-17.13710	G289967E33714S	NA	0
**GL151_DN_3**	Chilean Andes	17.02.22	16:50	5.4	"El Morado"	-33.7691	-70.0441	2731	3994	10.92	0.26	42.51	RGI60-17.13710	G289967E33714S	NA	3.11E-05
**GL152_UP_1**	Chilean Andes	18.02.22	12:50	1.8	"Colina"	-33.8605	-69.9229	2987	79	8.84	0.64	13.76	RGI60-17.13633	G290091E33826S	NA	0
**GL152_UP_2**	Chilean Andes	18.02.22	12:50	1.8	"Colina"	-33.8605	-69.9229	2987	79	8.84	0.64	13.76	RGI60-17.13633	G290091E33826S	NA	0
**GL152_UP_3**	Chilean Andes	18.02.22	12:50	1.8	"Colina"	-33.8605	-69.9229	2987	79	8.84	0.64	13.76	RGI60-17.13633	G290091E33826S	NA	0
**GL152_DN_1**	Chilean Andes	18.02.22	16:30	4.6	"Colina"	-33.8793	-69.9378	2716	2752	8.84	0.23	37.91	RGI60-17.13633	G290091E33826S	NA	0.0011785
**GL152_DN_2**	Chilean Andes	18.02.22	16:30	4.6	"Colina"	-33.8793	-69.9378	2716	2752	8.84	0.23	37.91	RGI60-17.13633	G290091E33826S	NA	0.0002435
**GL152_DN_3**	Chilean Andes	18.02.22	16:30	4.6	"Colina"	-33.8793	-69.9378	2716	2752	8.84	0.23	37.91	RGI60-17.13633	G290091E33826S	NA	0

**GL153_UP_1**	Chilean Andes	20.02.22	11:11	4.3	"El Morado cliffy"	-33.744	-70.055	3343	802	0.15	0.42	0.35	RGI60-17.13710	G289967E33714S	NA	0.026192
**GL153_UP_2**	Chilean Andes	20.02.22	11:11	4.3	"El Morado cliffy"	-33.744	-70.055	3343	802	0.15	0.42	0.35	RGI60-17.13710	G289967E33714S	NA	0.020358333
**GL153_UP_3**	Chilean Andes	20.02.22	11:11	4.3	"El Morado cliffy"	-33.744	-70.055	3343	802	0.15	0.42	0.35	RGI60-17.13710	G289967E33714S	NA	0.027149333
**GL153_DN_1**	Chilean Andes	20.02.22	14:17	7.2	"El Morado cliffy"	-33.744	-70.0527	3293	1014	0.15	0.41	0.36	RGI60-17.13710	G289967E33714S	NA	0.008420333
**GL153_DN_2**	Chilean Andes	20.02.22	14:17	7.2	"El Morado cliffy"	-33.744	-70.0527	3293	1014	0.15	0.41	0.36	RGI60-17.13710	G289967E33714S	NA	0.361250667
**GL153_DN_3**	Chilean Andes	20.02.22	14:17	7.2	"El Morado cliffy"	-33.744	-70.0527	3293	1014	0.15	0.41	0.36	RGI60-17.13710	G289967E33714S	NA	0.015333
**GL154_UP_1**	Chilean Andes	22.02.22	11:30	2.6	"Plomo West"	-33.5739	-69.9214	3495	3321	6.38	0.3	21.2	RGI60-17.13750	G290062E33534S	NA	0
**GL154_UP_2**	Chilean Andes	22.02.22	11:30	2.6	"Plomo West"	-33.5739	-69.9214	3495	3321	6.38	0.3	21.2	RGI60-17.13750	G290062E33534S	NA	0
**GL154_UP_3**	Chilean Andes	22.02.22	11:30	2.6	"Plomo West"	-33.5739	-69.9214	3495	3321	6.38	0.3	21.2	RGI60-17.13750	G290062E33534S	NA	0
**GL154_DN_1**	Chilean Andes	22.02.22	14:37	6	"Plomo West"	-33.5825	-69.9175	3359	4385	6.38	0.26	24.52	RGI60-17.13750	G290062E33534S	NA	0.004291333
**GL154_DN_2**	Chilean Andes	22.02.22	14:37	6	"Plomo West"	-33.5825	-69.9175	3359	4385	6.38	0.26	24.52	RGI60-17.13750	G290062E33534S	NA	0
**GL154_DN_3**	Chilean Andes	22.02.22	14:37	6	"Plomo West"	-33.5825	-69.9175	3359	4385	6.38	0.26	24.52	RGI60-17.13750	G290062E33534S	NA	0
**GL155_UP_1**	Chilean Andes	23.02.22	10:30	6.5	"Plomo rock glacier"	-33.6067	-69.9027	3169	254	7.47	0.31	23.91	RGI60-17.13720	G290109E33559S	NA	0.002478
**GL155_UP_2**	Chilean Andes	23.02.22	10:30	6.5	"Plomo rock glacier"	-33.6067	-69.9027	3169	254	7.47	0.31	23.91	RGI60-17.13720	G290109E33559S	NA	4.93E-05
**GL155_UP_3**	Chilean Andes	23.02.22	10:30	6.5	"Plomo rock glacier"	-33.6067	-69.9027	3169	254	7.47	0.31	23.91	RGI60-17.13720	G290109E33559S	NA	0.000494333
**GL155_DN_1**	Chilean Andes	23.02.22	13:25	3.7	"Plomo rock glacier"	-33.611	-69.9079	3003	963	7.47	0.31	24	RGI60-17.13720	G290109E33559S	NA	0.064509
**GL155_DN_2**	Chilean Andes	23.02.22	13:25	3.7	"Plomo rock glacier"	-33.611	-69.9079	3003	963	7.47	0.31	24	RGI60-17.13720	G290109E33559S	NA	0.497782
**GL155_DN_3**	Chilean Andes	23.02.22	13:25	3.7	"Plomo rock glacier"	-33.611	-69.9079	3003	963	7.47	0.31	24	RGI60-17.13720	G290109E33559S	NA	0.258341
**GL156_UP_1**	Alaska Range	21.06.22	12:43	0.3	Raven	61.066	-149.113	957	11	3.57	0.53	6.75	RGI60-01.08876	G210925E61062N	NA	0.002594667

**GL156_UP_2**	Alaska Range	21.06.22	12:43	0.3	Raven	61.066	-149.113	957	11	3.57	0.53	6.75	RGI60-01.08876	G210925E61062N	NA	0.010127667
**GL156_UP_3**	Alaska Range	21.06.22	12:43	0.3	Raven	61.066	-149.113	957	11	3.57	0.53	6.75	RGI60-01.08876	G210925E61062N	NA	0.002076
**GL156_DN_1**	Alaska Range	21.06.22	15:15	1.8	Raven	61.07	-149.12	870	618	3.58	0.46	7.79	RGI60-01.08876	G210925E61062N	NA	0
**GL156_DN_2**	Alaska Range	21.06.22	15:15	1.8	Raven	61.07	-149.12	870	618	3.58	0.46	7.79	RGI60-01.08876	G210925E61062N	NA	0
**GL156_DN_3**	Alaska Range	21.06.22	15:15	1.8	Raven	61.07	-149.12	870	618	3.58	0.46	7.79	RGI60-01.08876	G210925E61062N	NA	0
**GL157_UP_1**	Alaska Range	22.06.22	12:06	1.8	Leanard	60.7904	-148.725	233	806	3.14	0.43	7.29	RGI60-01.09507	G211285E60812N	173	0
**GL157_UP_2**	Alaska Range	22.06.22	12:06	1.8	Leanard	60.7904	-148.725	233	806	3.14	0.43	7.29	RGI60-01.09507	G211285E60812N	173	0.000747333
**GL157_UP_3**	Alaska Range	22.06.22	12:06	1.8	Leanard	60.7904	-148.725	233	806	3.14	0.43	7.29	RGI60-01.09507	G211285E60812N	173	0
**GL157_DN_1**	Alaska Range	22.06.22	15:56	2.5	Leanard	60.784	-148.717	0	2342	3.21	0.36	8.96	RGI60-01.09507	G211285E60812N	173	0.113876
**GL157_DN_2**	Alaska Range	22.06.22	15:56	2.5	Leanard	60.784	-148.717	0	2342	3.21	0.36	8.96	RGI60-01.09507	G211285E60812N	173	0.005568667
**GL157_DN_3**	Alaska Range	22.06.22	15:56	2.5	Leanard	60.784	-148.717	0	2342	3.21	0.36	8.96	RGI60-01.09507	G211285E60812N	173	0.140296
**GL158_UP_1**	Alaska Range	23.06.22	10:15	1	Porcupine	59.9992	-149.281	231	281	0.69	0.35	1.95	RGI60-01.08618	G210738E60001N	NA	0.007220667
**GL158_UP_2**	Alaska Range	23.06.22	10:15	1	Porcupine	59.9992	-149.281	231	281	0.69	0.35	1.95	RGI60-01.08618	G210738E60001N	NA	0.006928667
**GL158_UP_3**	Alaska Range	23.06.22	10:15	1	Porcupine	59.9992	-149.281	231	281	0.69	0.35	1.95	RGI60-01.08618	G210738E60001N	NA	0.149001333
**GL158_DN_1**	Alaska Range	23.06.22	13:55	2.6	Porcupine	59.9999	-149.292	46	911	0.69	0.22	3.15	RGI60-01.08618	G210738E60001N	NA	0.036385333
**GL158_DN_2**	Alaska Range	23.06.22	13:55	2.6	Porcupine	59.9999	-149.292	46	911	0.69	0.22	3.15	RGI60-01.08618	G210738E60001N	NA	0.222331667
**GL158_DN_3**	Alaska Range	23.06.22	13:55	2.6	Porcupine	59.9999	-149.292	46	911	0.69	0.22	3.15	RGI60-01.08618	G210738E60001N	NA	0.054498667
**GL159_UP_1**	Alaska Range	24.06.22	09:58	3.1	Byron	60.7588	-148.852	97	536	2.31	0.35	6.61	RGI60-01.09245	G211144E60742N	NA	0.001211333
**GL159_UP_2**	Alaska Range	24.06.22	09:58	3.1	Byron	60.7588	-148.852	97	536	2.31	0.35	6.61	RGI60-01.09245	G211144E60742N	NA	0.002807333

**GL159_UP_3**	Alaska Range	24.06.22	09:58	3.1	Byron	60.7588	-148.852	97	536	2.31	0.35	6.61	RGI60-01.09245	G211144E60742N	NA	0.016705
**GL159_DN_1**	Alaska Range	24.06.22	13:20	4.8	Byron	60.7677	-148.84	57	1738	2.35	0.27	8.59	RGI60-01.09245	G211144E60742N	NA	0.064446333
**GL159_DN_2**	Alaska Range	24.06.22	13:20	4.8	Byron	60.7677	-148.84	57	1738	2.35	0.27	8.59	RGI60-01.09245	G211144E60742N	NA	0.016383333
**GL159_DN_3**	Alaska Range	24.06.22	13:20	4.8	Byron	60.7677	-148.84	57	1738	2.35	0.27	8.59	RGI60-01.09245	G211144E60742N	NA	0.028002667
**GL160_UP_1**	Alaska Range	25.06.22	11:50	0.1	Milk	61.0454	-149.095	1123	1	1.99	0.78	2.53	RGI60-01.08875	G210925E61049N	NA	0
**GL160_UP_2**	Alaska Range	25.06.22	11:50	0.1	Milk	61.0454	-149.095	1123	1	1.99	0.78	2.53	RGI60-01.08875	G210925E61049N	NA	0
**GL160_UP_3**	Alaska Range	25.06.22	11:50	0.1	Milk	61.0454	-149.095	1123	1	1.99	0.78	2.53	RGI60-01.08875	G210925E61049N	NA	0
**GL160_DN_1**	Alaska Range	25.06.22	14:30	2.3	Milk	61.0433	-149.096	1062	240	1.99	0.76	2.62	RGI60-01.08875	G210925E61049N	NA	0
**GL160_DN_2**	Alaska Range	25.06.22	14:30	2.3	Milk	61.0433	-149.096	1062	240	1.99	0.76	2.62	RGI60-01.08875	G210925E61049N	NA	0
**GL160_DN_3**	Alaska Range	25.06.22	14:30	2.3	Milk	61.0433	-149.096	1062	240	1.99	0.76	2.62	RGI60-01.08875	G210925E61049N	NA	0
**GL161_UP_1**	Alaska Range	26.06.22	10:30	0.7	"South of Fourth of July Creek"	60.0866	-149.233	538	18	2.51	0.75	3.34	RGI60-01.08662	G210784E60074N	NA	0.005962
**GL161_UP_2**	Alaska Range	26.06.22	10:30	0.7	"South of Fourth of July Creek"	60.0866	-149.233	538	18	2.51	0.75	3.34	RGI60-01.08662	G210784E60074N	NA	0.001746333
**GL161_UP_3**	Alaska Range	26.06.22	10:30	0.7	"South of Fourth of July Creek"	60.0866	-149.233	538	18	2.51	0.75	3.34	RGI60-01.08662	G210784E60074N	NA	0.000263333
**GL161_DN_1**	Alaska Range	26.06.22	13:15	1.2	"South of Fourth of July Creek"	60.088	-149.235	481	236	2.79	0.69	4.05	RGI60-01.08662	G210784E60074N	NA	0.000925
**GL161_DN_2**	Alaska Range	26.06.22	13:15	1.2	"South of Fourth of July Creek"	60.088	-149.235	481	236	2.79	0.69	4.05	RGI60-01.08662	G210784E60074N	NA	0.004409
**GL161_DN_3**	Alaska Range	26.06.22	13:15	1.2	"South of Fourth of July Creek"	60.088	-149.235	481	236	2.79	0.69	4.05	RGI60-01.08662	G210784E60074N	NA	0.003523333
**GL162_UP_1**	Alaska Range	01.07.22	09:45	1.5	"Second west of Powell"	61.6413	-147.268	1520	33	4.91	0.54	9.11	RGI60-01.23597	G212739E61620N	NA	0
**GL162_UP_2**	Alaska Range	01.07.22	09:45	1.5	"Second west of Powell"	61.6413	-147.268	1520	33	4.91	0.54	9.11	RGI60-01.23597	G212739E61620N	NA	0
**GL162_UP_3**	Alaska Range	01.07.22	09:45	1.5	"Second west of Powell"	61.6413	-147.268	1520	33	4.91	0.54	9.11	RGI60-01.23597	G212739E61620N	NA	0

**GL162_DN_1**	Alaska Range	01.07.22	12:20	1.7	"Second west of Powell"	61.6433	-147.267	1476	272	4.92	0.51	9.59	RGI60-01.23597	G212739E61620N	NA	0
**GL162_DN_2**	Alaska Range	01.07.22	12:20	1.7	"Second west of Powell"	61.6433	-147.267	1476	272	4.92	0.51	9.59	RGI60-01.23597	G212739E61620N	NA	0
**GL162_DN_3**	Alaska Range	01.07.22	12:20	1.7	"Second west of Powell"	61.6433	-147.267	1476	272	4.92	0.51	9.59	RGI60-01.23597	G212739E61620N	NA	0
**GL163_UP_1**	Alaska Range	02.07.22	09:15	0.1	Powell	61.6704	-147.291	949	34	55.51	0.47	118.94	RGI60-01.10655	G212799E61568N	NA	0
**GL163_UP_2**	Alaska Range	02.07.22	09:15	0.1	Powell	61.6704	-147.291	949	34	55.51	0.47	118.94	RGI60-01.10655	G212799E61568N	NA	0
**GL163_UP_3**	Alaska Range	02.07.22	09:15	0.1	Powell	61.6704	-147.291	949	34	55.51	0.47	118.94	RGI60-01.10655	G212799E61568N	NA	0
**GL163_DN_1**	Alaska Range	02.07.22	12:15	0.9	Powell	61.6736	-147.295	858	607	55.52	0.46	119.42	RGI60-01.10655	G212799E61568N	NA	0
**GL163_DN_2**	Alaska Range	02.07.22	12:15	0.9	Powell	61.6736	-147.295	858	607	55.52	0.46	119.42	RGI60-01.10655	G212799E61568N	NA	0
**GL163_DN_3**	Alaska Range	02.07.22	12:15	0.9	Powell	61.6736	-147.295	858	607	55.52	0.46	119.42	RGI60-01.10655	G212799E61568N	NA	0
**GL164_UP_1**	Alaska Range	03.07.22	09:15	0.2	"Third west of Powell"	61.6701	-147.309	894	18	38.66	0.47	82.96	RGI60-01.10621	G212686E61576N	NA	0
**GL164_UP_2**	Alaska Range	03.07.22	09:15	0.2	"Third west of Powell"	61.6701	-147.309	894	18	38.66	0.47	82.96	RGI60-01.10621	G212686E61576N	NA	0
**GL164_UP_3**	Alaska Range	03.07.22	09:15	0.2	"Third west of Powell"	61.6701	-147.309	894	18	38.66	0.47	82.96	RGI60-01.10621	G212686E61576N	NA	0
**GL164_DN_1**	Alaska Range	03.07.22	12:00	1.3	"Third west of Powell"	61.6733	-147.31	848	377	38.66	0.46	83.62	RGI60-01.10621	G212686E61576N	NA	0
**GL164_DN_2**	Alaska Range	03.07.22	12:00	1.3	"Third west of Powell"	61.6733	-147.31	848	377	38.66	0.46	83.62	RGI60-01.10621	G212686E61576N	NA	0
**GL164_DN_3**	Alaska Range	03.07.22	12:00	1.3	"Third west of Powell"	61.6733	-147.31	848	377	38.66	0.46	83.62	RGI60-01.10621	G212686E61576N	NA	0
**GL165_UP_1**	Alaska Range	04.07.22	10:00	0.4	Nelchina	61.709	-147.074	758	832	323.45	0.56	579.92	RGI60-01.10683	G213074E61558N	NA	0
**GL165_UP_2**	Alaska Range	04.07.22	10:00	0.4	Nelchina	61.709	-147.074	758	832	323.45	0.56	579.92	RGI60-01.10683	G213074E61558N	NA	0
**GL165_UP_3**	Alaska Range	04.07.22	10:00	0.4	Nelchina	61.709	-147.074	758	832	323.45	0.56	579.92	RGI60-01.10683	G213074E61558N	NA	0
**GL165_DN_1**	Alaska Range	04.07.22	12:11	2	Nelchina	61.7156	-147.072	780	1640	323.53	0.56	580.75	RGI60-01.10683	G213074E61558N	NA	0

**GL165_DN_2**	Alaska Range	04.07.22	12:11	2	Nelchina	61.7156	-147.072	780	1640	323.53	0.56	580.75	RGI60-01.10683	G213074E61558N	NA	0
**GL165_DN_3**	Alaska Range	04.07.22	12:11	2	Nelchina	61.7156	-147.072	780	1640	323.53	0.56	580.75	RGI60-01.10683	G213074E61558N	NA	0
**GL166_UP_1**	Alaska Range	05.07.22	09:45	0.1	Sylvester	61.6779	-147.067	882	117	80.68	0.5	161.1	RGI60-01.23046	G212867E61496N	NA	0
**GL166_UP_2**	Alaska Range	05.07.22	09:45	0.1	Sylvester	61.6779	-147.067	882	117	80.68	0.5	161.1	RGI60-01.23046	G212867E61496N	NA	0
**GL166_UP_3**	Alaska Range	05.07.22	09:45	0.1	Sylvester	61.6779	-147.067	882	117	80.68	0.5	161.1	RGI60-01.23046	G212867E61496N	NA	0
**GL166_DN_1**	Alaska Range	05.07.22	12:00	0.4	Sylvester	61.683	-147.073	808	777	80.79	0.5	161.97	RGI60-01.23046	G212867E61496N	NA	0
**GL166_DN_2**	Alaska Range	05.07.22	12:00	0.4	Sylvester	61.683	-147.073	808	777	80.79	0.5	161.97	RGI60-01.23046	G212867E61496N	NA	0
**GL166_DN_3**	Alaska Range	05.07.22	12:00	0.4	Sylvester	61.683	-147.073	808	777	80.79	0.5	161.97	RGI60-01.23046	G212867E61496N	NA	0
**GL167_UP_1**	Alaska Range	06.07.22	11:19	2.4	Matanuska	61.7752	-147.762	472	559	322.32	0.53	604.91	RGI60-01.10557	G212412E61677N	NA	0.077877667
**GL167_UP_2**	Alaska Range	06.07.22	11:19	2.4	Matanuska	61.7752	-147.762	472	559	322.32	0.53	604.91	RGI60-01.10557	G212412E61677N	NA	0.097634667
**GL167_UP_3**	Alaska Range	06.07.22	11:19	2.4	Matanuska	61.7752	-147.762	472	559	322.32	0.53	604.91	RGI60-01.10557	G212412E61677N	NA	0.070152333
**GL167_DN_1**	Alaska Range	06.07.22	14:00	1.3	Matanuska	61.7877	-147.796	454	3090	322.41	0.53	610.21	RGI60-01.10557	G212412E61677N	NA	0.021069
**GL167_DN_2**	Alaska Range	06.07.22	14:00	1.3	Matanuska	61.7877	-147.796	454	3090	322.41	0.53	610.21	RGI60-01.10557	G212412E61677N	NA	0.005279333
**GL167_DN_3**	Alaska Range	06.07.22	14:00	1.3	Matanuska	61.7877	-147.796	454	3090	322.41	0.53	610.21	RGI60-01.10557	G212412E61677N	NA	0.019175
**GL168_UP_1**	Alaska Range	12.07.22	11:15	1.3	Rainbow Cirque	63.3208	-145.611	1496	9	0.63	0.78	0.8	RGI60-01.24172	G214393E63316N	NA	0.154746333
**GL168_UP_2**	Alaska Range	12.07.22	11:15	1.3	Rainbow Cirque	63.3208	-145.611	1496	9	0.63	0.78	0.8	RGI60-01.24172	G214393E63316N	NA	0.047866333
**GL168_UP_3**	Alaska Range	12.07.22	11:15	1.3	Rainbow Cirque	63.3208	-145.611	1496	9	0.63	0.78	0.8	RGI60-01.24172	G214393E63316N	NA	0.107327667
**GL168_DN_1**	Alaska Range	12.07.22	13:43	4.9	Rainbow Cirque	63.3247	-145.612	1373	464	0.85	0.52	1.64	RGI60-01.24172	G214393E63316N	NA	0.009750333
**GL168_DN_2**	Alaska Range	12.07.22	13:43	4.9	Rainbow Cirque	63.3247	-145.612	1373	464	0.85	0.52	1.64	RGI60-01.24172	G214393E63316N	NA	0.023281

**GL168_DN_3**	Alaska Range	12.07.22	13:43	4.9	Rainbow Cirque	63.3247	-145.612	1373	464	0.85	0.52	1.64	RGI60-01.24172	G214393E63316N	NA	0.020199
**GL169_UP_1**	Alaska Range	13.07.22	09:55	0.1	Castner	63.4044	-145.698	816	79	60.82	0.5	122.44	RGI60-01.00561	G214527E63453N	NA	0
**GL169_UP_2**	Alaska Range	13.07.22	09:55	0.1	Castner	63.4044	-145.698	816	79	60.82	0.5	122.44	RGI60-01.00561	G214527E63453N	NA	0
**GL169_UP_3**	Alaska Range	13.07.22	09:55	0.1	Castner	63.4044	-145.698	816	79	60.82	0.5	122.44	RGI60-01.00561	G214527E63453N	NA	0
**GL169_DN_1**	Alaska Range	13.07.22	12:30	0.3	Castner	63.4024	-145.714	772	959	60.85	0.49	124.05	RGI60-01.00561	G214527E63453N	NA	0
**GL169_DN_2**	Alaska Range	13.07.22	12:30	0.3	Castner	63.4024	-145.714	772	959	60.85	0.49	124.05	RGI60-01.00561	G214527E63453N	NA	0
**GL169_DN_3**	Alaska Range	13.07.22	12:30	0.3	Castner	63.4024	-145.714	772	959	60.85	0.49	124.05	RGI60-01.00561	G214527E63453N	NA	0
**GL170_UP_1**	Alaska Range	14.07.22	11:00	0.7	Gulkana	63.2551	-145.425	1300	26	10.86	0.66	16.52	RGI60-01.00570	G214573E63281N	90	0.000137333
**GL170_UP_2**	Alaska Range	14.07.22	11:00	0.7	Gulkana	63.2551	-145.425	1300	26	10.86	0.66	16.52	RGI60-01.00570	G214573E63281N	90	0.001123333
**GL170_UP_3**	Alaska Range	14.07.22	11:00	0.7	Gulkana	63.2551	-145.425	1300	26	10.86	0.66	16.52	RGI60-01.00570	G214573E63281N	90	0
**GL170_DN_1**	Alaska Range	14.07.22	14:22	2.1	Gulkana	63.2508	-145.445	1174	1122	11.64	0.64	18.32	RGI60-01.00570	G214573E63281N	90	0
**GL170_DN_2**	Alaska Range	14.07.22	14:22	2.1	Gulkana	63.2508	-145.445	1174	1122	11.64	0.64	18.32	RGI60-01.00570	G214573E63281N	90	0.001396
**GL170_DN_3**	Alaska Range	14.07.22	14:22	2.1	Gulkana	63.2508	-145.445	1174	1122	11.64	0.64	18.32	RGI60-01.00570	G214573E63281N	90	0.002061333

Supplementary Table 3 Leave-one-cluster-out tests output for the models of genome redundancy index. cluster = identifier of the phylogenetic cluster, p = p-value of the Wilcoxon signed rank test, median_effect = relative median difference in coefficient, padj = adjusted p-value, test = relationship that was tested.

cluster	p	median_effect	padj	test
**1**	0.017	0.285	1.000	KO redundancy ~ gl_dist
**2**	0.000	0.656	0.047	KO redundancy ~ gl_dist
**3**	0.002	0.435	0.853	KO redundancy ~ gl_dist
**4**	0.000	0.869	0.031	KO redundancy ~ gl_dist
**5**	0.001	0.555	0.410	KO redundancy ~ gl_dist
**6**	0.000	0.566	0.186	KO redundancy ~ gl_dist
**7**	0.001	0.513	0.263	KO redundancy ~ gl_dist
**8**	0.000	0.604	0.147	KO redundancy ~ gl_dist
**9**	0.002	0.395	0.943	KO redundancy ~ gl_dist

**10**	0.017	0.287	1.000	KO redundancy ~ gl_dist
**11**	0.102	0.289	1.000	KO redundancy ~ gl_dist
**12**	0.012	0.518	1.000	KO redundancy ~ gl_dist
**13**	0.327	0.115	1.000	KO redundancy ~ gl_dist
**14**	0.004	0.461	1.000	KO redundancy ~ gl_dist
**15**	0.000	0.934	0.047	KO redundancy ~ gl_dist
**16**	0.004	0.695	1.000	KO redundancy ~ gl_dist
**17**	0.010	0.476	1.000	KO redundancy ~ gl_dist
**18**	0.000	0.869	0.004	KO redundancy ~ gl_dist
**19**	0.018	0.486	1.000	KO redundancy ~ gl_dist
**20**	0.000	0.759	0.036	KO redundancy ~ gl_dist

**21**	0.033	0.322	1.000	KO redundancy ~ gl_dist
**22**	0.000	0.868	0.031	KO redundancy ~ gl_dist
**23**	0.004	0.373	1.000	KO redundancy ~ gl_dist
**24**	0.007	0.379	1.000	KO redundancy ~ gl_dist
**25**	0.005	0.552	1.000	KO redundancy ~ gl_dist
**26**	0.000	0.761	0.054	KO redundancy ~ gl_dist
**27**	0.002	0.428	0.696	KO redundancy ~ gl_dist
**28**	0.007	0.524	1.000	KO redundancy ~ gl_dist
**29**	0.000	0.808	0.079	KO redundancy ~ gl_dist
**30**	0.072	0.186	1.000	KO redundancy ~ gl_dist
**31**	0.012	0.454	1.000	KO redundancy ~ gl_dist

**32**	0.003	0.474	1.000	KO redundancy ~ gl_dist
**33**	0.005	0.513	1.000	KO redundancy ~ gl_dist
**34**	0.000	0.505	0.186	KO redundancy ~ gl_dist
**35**	0.001	0.499	0.457	KO redundancy ~ gl_dist
**36**	0.018	0.561	1.000	KO redundancy ~ gl_dist
**37**	0.012	0.343	1.000	KO redundancy ~ gl_dist
**38**	0.009	0.743	1.000	KO redundancy ~ gl_dist
**39**	0.000	0.711	0.090	KO redundancy ~ gl_dist
**40**	0.000	0.590	0.090	KO redundancy ~ gl_dist
**41**	0.001	0.591	0.209	KO redundancy ~ gl_dist
**42**	0.002	0.627	0.771	KO redundancy ~ gl_dist

**43**	0.001	0.557	0.263	KO redundancy ~ gl_dist
**44**	0.001	0.557	0.263	KO redundancy ~ gl_dist
**45**	0.000	0.768	0.011	KO redundancy ~ gl_dist
**46**	0.001	0.563	0.263	KO redundancy ~ gl_dist
**47**	0.001	0.678	0.235	KO redundancy ~ gl_dist
**48**	0.000	0.565	0.165	KO redundancy ~ gl_dist
**49**	0.000	0.668	0.047	KO redundancy ~ gl_dist
**50**	0.001	0.587	0.209	KO redundancy ~ gl_dist
**51**	0.001	0.566	0.209	KO redundancy ~ gl_dist
**52**	0.001	0.562	0.209	KO redundancy ~ gl_dist
**53**	0.001	0.567	0.209	KO redundancy ~ gl_dist

**54**	0.001	0.567	0.209	KO redundancy ~ gl_dist
**55**	0.001	0.552	0.457	KO redundancy ~ gl_dist
**56**	0.001	0.567	0.209	KO redundancy ~ gl_dist
**57**	0.001	0.567	0.209	KO redundancy ~ gl_dist
**58**	0.001	0.567	0.209	KO redundancy ~ gl_dist
**59**	0.000	0.642	0.102	KO redundancy ~ gl_dist
**60**	0.000	0.771	0.007	KO redundancy ~ gl_dist
**61**	0.001	0.561	0.294	KO redundancy ~ gl_dist
**62**	0.000	0.666	0.027	KO redundancy ~ gl_dist
**63**	0.000	0.465	0.165	KO redundancy ~ gl_dist
**64**	0.004	0.420	1.000	KO redundancy ~ gl_dist

**65**	0.000	0.492	0.102	KO redundancy ~ gl_dist
**66**	0.001	0.662	0.368	KO redundancy ~ gl_dist
**67**	0.001	0.468	0.457	KO redundancy ~ gl_dist
**68**	0.001	0.560	0.565	KO redundancy ~ gl_dist
**69**	0.035	0.212	1.000	KO redundancy ~ gl_dist
**70**	0.000	0.521	0.147	KO redundancy ~ gl_dist
**71**	0.000	0.527	0.165	KO redundancy ~ gl_dist
**72**	0.000	0.469	0.147	KO redundancy ~ gl_dist
**73**	0.001	0.436	0.457	KO redundancy ~ gl_dist
**74**	0.000	0.437	0.186	KO redundancy ~ gl_dist
**75**	0.001	0.544	0.368	KO redundancy ~ gl_dist

**76**	0.001	0.519	0.294	KO redundancy ~ gl_dist
**77**	0.000	0.599	0.186	KO redundancy ~ gl_dist
**78**	0.000	0.573	0.165	KO redundancy ~ gl_dist
**79**	0.001	0.575	0.209	KO redundancy ~ gl_dist
**80**	0.001	0.562	0.329	KO redundancy ~ gl_dist
**81**	0.001	0.563	0.329	KO redundancy ~ gl_dist
**82**	0.002	0.378	0.696	KO redundancy ~ gl_dist
**83**	0.000	0.499	0.186	KO redundancy ~ gl_dist
**84**	0.000	0.497	0.115	KO redundancy ~ gl_dist
**85**	0.001	0.492	0.294	KO redundancy ~ gl_dist
**86**	0.001	0.504	0.263	KO redundancy ~ gl_dist

**87**	0.002	0.457	0.696	KO redundancy ~ gl_dist
**88**	0.000	0.508	0.186	KO redundancy ~ gl_dist
**89**	0.001	0.496	0.368	KO redundancy ~ gl_dist
**90**	0.002	0.462	0.628	KO redundancy ~ gl_dist
**91**	0.003	0.394	1.000	KO redundancy ~ gl_dist
**92**	0.002	0.464	0.628	KO redundancy ~ gl_dist
**93**	0.002	0.458	0.696	KO redundancy ~ gl_dist
**94**	0.002	0.461	0.696	KO redundancy ~ gl_dist
**95**	0.002	0.461	0.696	KO redundancy ~ gl_dist
**96**	0.001	0.496	0.410	KO redundancy ~ gl_dist
**97**	0.002	0.468	0.943	KO redundancy ~ gl_dist

**98**	0.002	0.441	0.943	KO redundancy ~ gl_dist
**99**	0.003	0.495	1.000	KO redundancy ~ gl_dist
**100**	0.003	0.497	1.000	KO redundancy ~ gl_dist
**101**	0.017	0.285	1.000	KO redundancy ~ gl_dist
**102**	0.017	0.280	1.000	KO redundancy ~ gl_dist
**103**	0.003	0.501	1.000	KO redundancy ~ gl_dist
**104**	0.017	0.284	1.000	KO redundancy ~ gl_dist
**105**	0.002	0.481	0.628	KO redundancy ~ gl_dist
**106**	0.001	0.756	0.294	KO redundancy ~ gl_dist
**107**	0.014	0.439	1.000	KO redundancy ~ gl_dist
**108**	0.000	0.701	0.079	KO redundancy ~ gl_dist

**109**	0.015	0.283	1.000	KO redundancy ~ gl_dist
**110**	0.012	0.337	1.000	KO redundancy ~ gl_dist
**111**	0.001	0.629	0.294	KO redundancy ~ gl_dist
**112**	0.002	0.747	0.853	KO redundancy ~ gl_dist
**113**	0.000	0.720	0.090	KO redundancy ~ gl_dist
**114**	0.000	0.703	0.115	KO redundancy ~ gl_dist
**115**	0.001	0.659	0.294	KO redundancy ~ gl_dist
**116**	0.142	0.129	1.000	KO redundancy ~ gl_dist
**117**	0.001	0.574	0.368	KO redundancy ~ gl_dist
**118**	0.000	0.816	0.079	KO redundancy ~ gl_dist
**119**	0.000	0.717	0.054	KO redundancy ~ gl_dist

**120**	0.026	0.343	1.000	KO redundancy ~ gl_dist
**121**	0.026	0.344	1.000	KO redundancy ~ gl_dist
**122**	0.002	0.513	0.771	KO redundancy ~ gl_dist
**123**	0.000	0.634	0.061	KO redundancy ~ gl_dist
**124**	0.020	0.324	1.000	KO redundancy ~ gl_dist
**125**	0.020	0.323	1.000	KO redundancy ~ gl_dist
**126**	0.000	0.865	0.006	KO redundancy ~ gl_dist
**127**	0.000	0.887	0.130	KO redundancy ~ gl_dist
**128**	0.035	0.259	1.000	KO redundancy ~ gl_dist
**129**	0.035	0.259	1.000	KO redundancy ~ gl_dist
**130**	0.091	0.216	1.000	KO redundancy ~ gl_dist

**131**	0.002	0.503	0.943	KO redundancy ~ gl_dist
**132**	0.014	0.504	1.000	KO redundancy ~ gl_dist
**133**	0.000	0.965	0.001	KO redundancy ~ gl_dist
**134**	0.038	0.465	1.000	KO redundancy ~ gl_dist
**135**	0.052	0.276	1.000	KO redundancy ~ gl_dist
**136**	0.072	0.282	1.000	KO redundancy ~ gl_dist
**137**	0.002	0.630	0.696	KO redundancy ~ gl_dist
**138**	0.030	0.272	1.000	KO redundancy ~ gl_dist
**139**	0.030	0.272	1.000	KO redundancy ~ gl_dist
**140**	0.026	0.175	1.000	KO redundancy ~ gl_dist
**141**	0.026	0.176	1.000	KO redundancy ~ gl_dist

**142**	0.026	0.174	1.000	KO redundancy ~ gl_dist
**143**	0.026	0.178	1.000	KO redundancy ~ gl_dist
**144**	0.020	0.198	1.000	KO redundancy ~ gl_dist
**145**	0.000	0.994	0.024	KO redundancy ~ gl_dist
**146**	0.023	0.182	1.000	KO redundancy ~ gl_dist
**147**	0.000	1.022	0.021	KO redundancy ~ gl_dist
**148**	0.000	0.936	0.070	KO redundancy ~ gl_dist
**149**	0.023	0.181	1.000	KO redundancy ~ gl_dist
**150**	0.028	0.196	1.000	KO redundancy ~ gl_dist
**151**	0.028	0.196	1.000	KO redundancy ~ gl_dist
**152**	0.028	0.197	1.000	KO redundancy ~ gl_dist

**153**	0.000	0.944	0.041	KO redundancy ~ gl_dist
**154**	0.030	0.205	1.000	KO redundancy ~ gl_dist
**155**	0.002	0.695	0.628	KO redundancy ~ gl_dist
**156**	0.000	0.717	0.006	KO redundancy ~ gl_dist
**157**	0.040	0.216	1.000	KO redundancy ~ gl_dist
**158**	0.038	0.218	1.000	KO redundancy ~ gl_dist
**159**	0.040	0.217	1.000	KO redundancy ~ gl_dist
**160**	0.000	0.591	0.186	KO redundancy ~ gl_dist
**161**	0.028	0.209	1.000	KO redundancy ~ gl_dist
**162**	0.000	0.900	0.130	KO redundancy ~ gl_dist
**163**	0.052	0.156	1.000	KO redundancy ~ gl_dist

**164**	0.052	0.155	1.000	KO redundancy ~ gl_dist
**165**	0.052	0.156	1.000	KO redundancy ~ gl_dist
**166**	0.052	0.155	1.000	KO redundancy ~ gl_dist
**167**	0.056	0.147	1.000	KO redundancy ~ gl_dist
**168**	0.000	0.852	0.102	KO redundancy ~ gl_dist
**169**	0.001	0.546	0.329	KO redundancy ~ gl_dist
**170**	0.000	0.808	0.090	KO redundancy ~ gl_dist
**171**	0.018	0.283	1.000	KO redundancy ~ gl_dist
**172**	0.000	0.545	0.079	KO redundancy ~ gl_dist
**173**	0.018	0.283	1.000	KO redundancy ~ gl_dist
**174**	0.018	0.283	1.000	KO redundancy ~ gl_dist

**175**	0.018	0.283	1.000	KO redundancy ~ gl_dist
**176**	0.000	0.544	0.079	KO redundancy ~ gl_dist
**177**	0.018	0.283	1.000	KO redundancy ~ gl_dist
**178**	0.000	0.544	0.079	KO redundancy ~ gl_dist
**179**	0.000	0.793	0.061	KO redundancy ~ gl_dist
**180**	0.015	0.294	1.000	KO redundancy ~ gl_dist
**181**	0.015	0.294	1.000	KO redundancy ~ gl_dist
**182**	0.015	0.292	1.000	KO redundancy ~ gl_dist
**183**	0.015	0.294	1.000	KO redundancy ~ gl_dist
**184**	0.201	0.090	1.000	KO redundancy ~ gl_dist
**185**	0.000	0.589	0.090	KO redundancy ~ gl_dist

**186**	0.004	0.528	1.000	KO redundancy ~ gl_dist
**187**	0.001	0.595	0.263	KO redundancy ~ gl_dist
**188**	0.000	0.587	0.090	KO redundancy ~ gl_dist
**189**	0.000	0.769	0.165	KO redundancy ~ gl_dist
**190**	0.000	0.808	0.031	KO redundancy ~ gl_dist
**191**	0.001	0.530	0.565	KO redundancy ~ gl_dist
**192**	0.001	0.536	0.565	KO redundancy ~ gl_dist
**193**	0.000	0.424	0.102	KO redundancy ~ gl_dist
**194**	0.010	0.362	1.000	KO redundancy ~ gl_dist
**195**	0.000	0.661	0.165	KO redundancy ~ gl_dist
**196**	0.008	0.422	1.000	KO redundancy ~ gl_dist

**197**	0.000	0.550	0.079	KO redundancy ~ gl_dist
**198**	0.001	0.563	0.509	KO redundancy ~ gl_dist
**199**	0.000	0.589	0.070	KO redundancy ~ gl_dist
**200**	0.086	0.324	1.000	KO redundancy ~ gl_dist
**201**	0.007	0.718	1.000	KO redundancy ~ gl_dist
**202**	0.001	0.508	0.294	KO redundancy ~ gl_dist
**203**	0.009	0.613	1.000	KO redundancy ~ gl_dist
**204**	0.013	0.498	1.000	KO redundancy ~ gl_dist
**205**	0.035	0.194	1.000	KO redundancy ~ gl_dist
**206**	0.035	0.164	1.000	KO redundancy ~ gl_dist
**207**	0.002	0.712	0.696	KO redundancy ~ gl_dist

**208**	0.134	0.256	1.000	KO redundancy ~ gl_dist
**209**	0.012	0.582	1.000	KO redundancy ~ gl_dist
**210**	0.021	0.545	1.000	KO redundancy ~ gl_dist
**211**	0.002	0.498	0.696	KO redundancy ~ gl_dist
**212**	0.002	0.498	0.696	KO redundancy ~ gl_dist
**213**	0.021	0.546	1.000	KO redundancy ~ gl_dist
**214**	0.021	0.546	1.000	KO redundancy ~ gl_dist
**215**	0.000	0.654	0.090	KO redundancy ~ gl_dist
**216**	0.023	0.361	1.000	KO redundancy ~ gl_dist
**217**	0.000	0.820	0.079	KO redundancy ~ gl_dist
**218**	0.020	0.269	1.000	KO redundancy ~ gl_dist

**219**	0.002	0.527	0.943	KO redundancy ~ gl_dist
**220**	0.002	0.666	0.943	KO redundancy ~ gl_dist
**221**	0.001	0.685	0.509	KO redundancy ~ gl_dist
**222**	0.002	0.671	0.853	KO redundancy ~ gl_dist
**223**	0.002	0.669	0.853	KO redundancy ~ gl_dist
**224**	0.002	0.668	0.943	KO redundancy ~ gl_dist
**225**	0.108	0.304	1.000	KO redundancy ~ gl_dist
**226**	0.004	0.388	1.000	KO redundancy ~ gl_dist
**227**	0.000	0.748	0.102	KO redundancy ~ gl_dist
**228**	0.001	0.507	0.368	KO redundancy ~ gl_dist
**229**	0.000	0.911	0.079	KO redundancy ~ gl_dist

**230**	0.004	0.373	1.000	KO redundancy ~ gl_dist
**231**	0.004	0.374	1.000	KO redundancy ~ gl_dist
**232**	0.000	0.741	0.024	KO redundancy ~ gl_dist
**233**	0.030	0.263	1.000	KO redundancy ~ gl_dist
**234**	0.000	0.679	0.115	KO redundancy ~ gl_dist
**235**	0.021	0.232	1.000	KO redundancy ~ gl_dist
**236**	0.021	0.235	1.000	KO redundancy ~ gl_dist
**237**	0.001	0.680	0.410	KO redundancy ~ gl_dist
**238**	0.001	0.657	0.209	KO redundancy ~ gl_dist
**239**	0.040	0.231	1.000	KO redundancy ~ gl_dist
**240**	0.038	0.240	1.000	KO redundancy ~ gl_dist

**241**	0.038	0.238	1.000	KO redundancy ~ gl_dist
**242**	0.038	0.239	1.000	KO redundancy ~ gl_dist
**243**	0.038	0.239	1.000	KO redundancy ~ gl_dist
**244**	0.001	0.513	0.457	KO redundancy ~ gl_dist
**245**	0.000	0.870	0.018	KO redundancy ~ gl_dist
**246**	0.005	0.555	1.000	KO redundancy ~ gl_dist
**247**	0.005	0.555	1.000	KO redundancy ~ gl_dist
**248**	0.004	0.565	1.000	KO redundancy ~ gl_dist
**249**	0.012	0.271	1.000	KO redundancy ~ gl_dist
**250**	0.006	0.541	1.000	KO redundancy ~ gl_dist
**251**	0.033	0.236	1.000	KO redundancy ~ gl_dist

**252**	0.005	0.509	1.000	KO redundancy ~ gl_dist
**253**	0.005	0.509	1.000	KO redundancy ~ gl_dist
**254**	0.002	0.498	0.853	KO redundancy ~ gl_dist
**255**	0.002	0.472	0.628	KO redundancy ~ gl_dist
**256**	0.015	0.296	1.000	KO redundancy ~ gl_dist
**257**	0.002	0.560	0.771	KO redundancy ~ gl_dist
**258**	0.000	0.815	0.015	KO redundancy ~ gl_dist
**259**	0.014	0.300	1.000	KO redundancy ~ gl_dist
**260**	0.005	0.651	1.000	KO redundancy ~ gl_dist
**261**	0.002	0.544	0.628	KO redundancy ~ gl_dist
**262**	0.002	0.527	0.853	KO redundancy ~ gl_dist

**263**	0.002	0.671	0.943	KO redundancy ~ gl_dist
**264**	0.000	0.767	0.013	KO redundancy ~ gl_dist
**265**	0.001	0.497	0.209	KO redundancy ~ gl_dist
**266**	0.024	0.170	1.000	KO redundancy ~ gl_dist
**267**	0.010	0.375	1.000	KO redundancy ~ gl_dist
**268**	0.108	0.114	1.000	KO redundancy ~ gl_dist
**269**	0.023	0.431	1.000	KO redundancy ~ gl_dist
**270**	0.001	0.467	0.329	KO redundancy ~ gl_dist
**271**	0.000	0.529	0.061	KO redundancy ~ gl_dist
**272**	0.021	0.405	1.000	KO redundancy ~ gl_dist
**273**	0.000	0.611	0.102	KO redundancy ~ gl_dist

**274**	0.000	0.822	0.090	KO redundancy ~ gl_dist
**275**	0.001	0.613	0.294	KO redundancy ~ gl_dist
**276**	0.011	0.417	1.000	KO redundancy ~ gl_dist
**277**	0.001	0.664	0.209	KO redundancy ~ gl_dist
**278**	0.001	0.668	0.209	KO redundancy ~ gl_dist
**279**	0.001	0.486	0.294	KO redundancy ~ gl_dist
**280**	0.046	0.291	1.000	KO redundancy ~ gl_dist
**281**	0.012	0.491	1.000	KO redundancy ~ gl_dist
**282**	0.000	0.746	0.001	KO redundancy ~ gl_dist
**283**	0.002	0.576	0.771	KO redundancy ~ gl_dist
**284**	0.035	0.185	1.000	KO redundancy ~ gl_dist

**285**	0.004	0.638	1.000	KO redundancy ~ gl_dist
**286**	0.010	0.243	1.000	KO redundancy ~ gl_dist
**287**	0.009	0.251	1.000	KO redundancy ~ gl_dist
**288**	0.003	0.594	1.000	KO redundancy ~ gl_dist
**289**	0.000	0.587	0.027	KO redundancy ~ gl_dist
**290**	0.006	0.669	1.000	KO redundancy ~ gl_dist
**291**	0.026	0.339	1.000	KO redundancy ~ gl_dist
**292**	0.026	0.340	1.000	KO redundancy ~ gl_dist
**293**	0.026	0.339	1.000	KO redundancy ~ gl_dist
**294**	0.026	0.338	1.000	KO redundancy ~ gl_dist
**295**	0.000	0.545	0.015	KO redundancy ~ gl_dist

**296**	0.003	0.585	1.000	KO redundancy ~ gl_dist
**297**	0.000	0.731	0.006	KO redundancy ~ gl_dist
**298**	0.028	0.263	1.000	KO redundancy ~ gl_dist
**299**	0.000	0.626	0.090	KO redundancy ~ gl_dist
**300**	0.014	0.499	1.000	KO redundancy ~ gl_dist
**301**	0.015	0.509	1.000	KO redundancy ~ gl_dist
**302**	0.018	0.505	1.000	KO redundancy ~ gl_dist
**303**	0.072	0.231	1.000	KO redundancy ~ gl_dist
**304**	0.006	0.463	1.000	KO redundancy ~ gl_dist
**305**	0.091	0.311	1.000	KO redundancy ~ gl_dist
**306**	0.000	0.895	0.102	KO redundancy ~ gl_dist

**307**	0.142	0.136	1.000	KO redundancy ~ gl_dist
**308**	0.002	0.629	0.771	KO redundancy ~ gl_dist
**309**	0.007	0.637	1.000	KO redundancy ~ gl_dist
**310**	0.004	0.514	1.000	KO redundancy ~ gl_dist
**311**	0.017	0.415	1.000	KO redundancy ~ gl_dist
**312**	0.017	0.420	1.000	KO redundancy ~ gl_dist
**313**	0.005	0.593	1.000	KO redundancy ~ gl_dist
**314**	0.000	1.666	0.000	KO redundancy ~ gl_dist
**315**	0.004	0.481	1.000	KO redundancy ~ gl_dist
**316**	0.004	0.481	1.000	KO redundancy ~ gl_dist
**317**	0.004	0.543	1.000	KO redundancy ~ gl_dist

**318**	0.000	0.909	0.004	KO redundancy ~ gl_dist
**319**	0.003	0.471	1.000	KO redundancy ~ gl_dist
**320**	0.000	0.915	0.102	KO redundancy ~ gl_dist
**321**	0.004	0.429	1.000	KO redundancy ~ gl_dist
**322**	0.005	0.416	1.000	KO redundancy ~ gl_dist
**323**	0.003	0.476	1.000	KO redundancy ~ gl_dist
**324**	0.005	0.416	1.000	KO redundancy ~ gl_dist
**325**	0.005	0.415	1.000	KO redundancy ~ gl_dist
**326**	0.000	0.893	0.002	KO redundancy ~ gl_dist
**327**	0.002	0.482	0.943	KO redundancy ~ gl_dist
**328**	0.002	0.481	0.943	KO redundancy ~ gl_dist

**329**	0.000	0.639	0.024	KO redundancy ~ gl_dist
**330**	0.005	0.513	1.000	KO redundancy ~ gl_dist
**331**	0.000	0.889	0.008	KO redundancy ~ gl_dist
**332**	0.000	0.800	0.047	KO redundancy ~ gl_dist
**333**	0.020	0.312	1.000	KO redundancy ~ gl_dist
**334**	0.017	0.305	1.000	KO redundancy ~ gl_dist
**335**	0.001	0.635	0.209	KO redundancy ~ gl_dist
**336**	0.004	0.516	1.000	KO redundancy ~ gl_dist
**337**	0.010	0.758	1.000	KO redundancy ~ gl_dist
**338**	0.003	0.542	1.000	KO redundancy ~ gl_dist
**339**	0.000	0.506	0.186	KO redundancy ~ gl_dist

**340**	0.000	0.506	0.186	KO redundancy ~ gl_dist
**341**	0.000	0.888	0.061	KO redundancy ~ gl_dist
**342**	0.001	0.454	0.509	KO redundancy ~ gl_dist
**343**	0.001	0.470	0.565	KO redundancy ~ gl_dist
**344**	0.000	0.587	0.186	KO redundancy ~ gl_dist
**345**	0.002	0.370	0.943	KO redundancy ~ gl_dist
**346**	0.002	0.370	0.943	KO redundancy ~ gl_dist
**347**	0.002	0.371	0.943	KO redundancy ~ gl_dist
**348**	0.000	0.672	0.018	KO redundancy ~ gl_dist
**349**	0.002	0.507	0.771	KO redundancy ~ gl_dist
**350**	0.001	0.526	0.329	KO redundancy ~ gl_dist

**351**	0.011	0.501	1.000	KO redundancy ~ gl_dist
**352**	0.001	0.525	0.329	KO redundancy ~ gl_dist
**353**	0.010	0.511	1.000	KO redundancy ~ gl_dist
**354**	0.000	0.715	0.036	KO redundancy ~ gl_dist
**355**	0.289	0.034	1.000	KO redundancy ~ gl_dist
**356**	0.002	0.535	0.853	KO redundancy ~ gl_dist
**357**	0.000	0.895	0.041	KO redundancy ~ gl_dist
**358**	0.017	0.453	1.000	KO redundancy ~ gl_dist
**359**	0.000	0.696	0.070	KO redundancy ~ gl_dist
**360**	0.000	0.758	0.061	KO redundancy ~ gl_dist
**361**	0.018	0.338	1.000	KO redundancy ~ gl_dist

**362**	0.001	0.693	0.209	KO redundancy ~ gl_dist
**363**	0.004	0.498	1.000	KO redundancy ~ gl_dist
**364**	0.000	0.860	0.079	KO redundancy ~ gl_dist
**365**	0.011	0.550	1.000	KO redundancy ~ gl_dist
**366**	0.001	0.533	0.565	KO redundancy ~ gl_dist
**367**	0.012	0.343	1.000	KO redundancy ~ gl_dist
**368**	0.001	0.633	0.565	KO redundancy ~ gl_dist
**369**	0.000	0.717	0.186	KO redundancy ~ gl_dist
**370**	0.000	0.850	0.018	KO redundancy ~ gl_dist
**371**	0.012	0.317	1.000	KO redundancy ~ gl_dist
**372**	0.000	0.756	0.054	KO redundancy ~ gl_dist

**373**	0.001	0.550	0.263	KO redundancy ~ gl_dist
**374**	0.002	0.734	0.853	KO redundancy ~ gl_dist
**375**	0.000	0.562	0.186	KO redundancy ~ gl_dist
**376**	0.002	0.375	0.853	KO redundancy ~ gl_dist
**377**	0.002	0.375	0.853	KO redundancy ~ gl_dist
**378**	0.002	0.377	0.853	KO redundancy ~ gl_dist
**379**	0.030	0.394	1.000	KO redundancy ~ gl_dist
**380**	0.003	0.402	1.000	KO redundancy ~ gl_dist
**381**	0.002	0.739	0.628	KO redundancy ~ gl_dist
**382**	0.002	0.483	0.696	KO redundancy ~ gl_dist
**383**	0.003	0.453	1.000	KO redundancy ~ gl_dist

**384**	0.005	0.585	1.000	KO redundancy ~ gl_dist
**385**	0.020	0.415	1.000	KO redundancy ~ gl_dist
**386**	0.659	-0.003	1.000	KO redundancy ~ gl_dist
**387**	0.008	0.589	1.000	KO redundancy ~ gl_dist
**388**	0.000	0.488	0.115	KO redundancy ~ gl_dist
**389**	0.009	0.672	1.000	KO redundancy ~ gl_dist
**390**	0.009	0.392	1.000	KO redundancy ~ gl_dist
**391**	0.002	0.663	0.853	KO redundancy ~ gl_dist
**392**	0.002	0.713	0.771	KO redundancy ~ gl_dist
**393**	0.000	0.760	0.010	KO redundancy ~ gl_dist
**394**	0.049	0.324	1.000	KO redundancy ~ gl_dist

**1**	0.277	0.128	1.000	KO redundancy ~ watemp
**2**	0.033	0.188	1.000	KO redundancy ~ watemp
**3**	0.142	0.195	1.000	KO redundancy ~ watemp
**4**	0.010	0.276	1.000	KO redundancy ~ watemp
**5**	0.052	0.194	1.000	KO redundancy ~ watemp
**6**	0.060	0.163	1.000	KO redundancy ~ watemp
**7**	0.063	0.136	1.000	KO redundancy ~ watemp
**8**	0.004	0.289	1.000	KO redundancy ~ watemp
**9**	0.035	0.229	1.000	KO redundancy ~ watemp
**10**	0.277	0.129	1.000	KO redundancy ~ watemp
**11**	0.142	0.112	1.000	KO redundancy ~ watemp

**12**	0.001	0.482	0.368	KO redundancy ~ watemp
**13**	0.925	-0.072	1.000	KO redundancy ~ watemp
**14**	0.142	0.176	1.000	KO redundancy ~ watemp
**15**	0.211	0.178	1.000	KO redundancy ~ watemp
**16**	0.091	0.130	1.000	KO redundancy ~ watemp
**17**	0.096	0.218	1.000	KO redundancy ~ watemp
**18**	0.026	0.172	1.000	KO redundancy ~ watemp
**19**	0.086	0.165	1.000	KO redundancy ~ watemp
**20**	0.242	0.165	1.000	KO redundancy ~ watemp
**21**	0.355	0.048	1.000	KO redundancy ~ watemp
**22**	0.086	0.160	1.000	KO redundancy ~ watemp

**23**	0.201	0.168	1.000	KO redundancy ~ watemp
**24**	0.121	0.119	1.000	KO redundancy ~ watemp
**25**	0.086	0.173	1.000	KO redundancy ~ watemp
**26**	0.127	0.103	1.000	KO redundancy ~ watemp
**27**	0.461	0.030	1.000	KO redundancy ~ watemp
**28**	0.355	0.025	1.000	KO redundancy ~ watemp
**29**	0.114	0.265	1.000	KO redundancy ~ watemp
**30**	0.314	0.153	1.000	KO redundancy ~ watemp
**31**	0.327	0.133	1.000	KO redundancy ~ watemp
**32**	0.096	0.241	1.000	KO redundancy ~ watemp
**33**	0.183	0.178	1.000	KO redundancy ~ watemp

**34**	0.201	0.146	1.000	KO redundancy ~ watemp
**35**	0.096	0.201	1.000	KO redundancy ~ watemp
**36**	0.068	0.205	1.000	KO redundancy ~ watemp
**37**	0.063	0.246	1.000	KO redundancy ~ watemp
**38**	0.017	0.259	1.000	KO redundancy ~ watemp
**39**	0.063	0.224	1.000	KO redundancy ~ watemp
**40**	0.060	0.226	1.000	KO redundancy ~ watemp
**41**	0.030	0.174	1.000	KO redundancy ~ watemp
**42**	0.026	0.272	1.000	KO redundancy ~ watemp
**43**	0.063	0.227	1.000	KO redundancy ~ watemp
**44**	0.063	0.226	1.000	KO redundancy ~ watemp

**45**	0.007	0.324	1.000	KO redundancy ~ watemp
**46**	0.046	0.156	1.000	KO redundancy ~ watemp
**47**	0.024	0.264	1.000	KO redundancy ~ watemp
**48**	0.038	0.165	1.000	KO redundancy ~ watemp
**49**	0.006	0.289	1.000	KO redundancy ~ watemp
**50**	0.056	0.161	1.000	KO redundancy ~ watemp
**51**	0.060	0.155	1.000	KO redundancy ~ watemp
**52**	0.060	0.152	1.000	KO redundancy ~ watemp
**53**	0.060	0.154	1.000	KO redundancy ~ watemp
**54**	0.060	0.154	1.000	KO redundancy ~ watemp
**55**	0.052	0.193	1.000	KO redundancy ~ watemp

**56**	0.060	0.154	1.000	KO redundancy ~ watemp
**57**	0.060	0.154	1.000	KO redundancy ~ watemp
**58**	0.060	0.154	1.000	KO redundancy ~ watemp
**59**	0.023	0.299	1.000	KO redundancy ~ watemp
**60**	0.007	0.326	1.000	KO redundancy ~ watemp
**61**	0.052	0.193	1.000	KO redundancy ~ watemp
**62**	0.018	0.199	1.000	KO redundancy ~ watemp
**63**	0.018	0.283	1.000	KO redundancy ~ watemp
**64**	0.035	0.211	1.000	KO redundancy ~ watemp
**65**	0.014	0.203	1.000	KO redundancy ~ watemp
**66**	0.020	0.238	1.000	KO redundancy ~ watemp

**67**	0.052	0.181	1.000	KO redundancy ~ watemp
**68**	0.028	0.289	1.000	KO redundancy ~ watemp
**69**	0.429	-0.022	1.000	KO redundancy ~ watemp
**70**	0.013	0.260	1.000	KO redundancy ~ watemp
**71**	0.020	0.223	1.000	KO redundancy ~ watemp
**72**	0.014	0.279	1.000	KO redundancy ~ watemp
**73**	0.033	0.211	1.000	KO redundancy ~ watemp
**74**	0.024	0.218	1.000	KO redundancy ~ watemp
**75**	0.056	0.140	1.000	KO redundancy ~ watemp
**76**	0.023	0.254	1.000	KO redundancy ~ watemp
**77**	0.013	0.216	1.000	KO redundancy ~ watemp

**78**	0.006	0.278	1.000	KO redundancy ~ watemp
**79**	0.035	0.179	1.000	KO redundancy ~ watemp
**80**	0.006	0.271	1.000	KO redundancy ~ watemp
**81**	0.006	0.272	1.000	KO redundancy ~ watemp
**82**	0.014	0.235	1.000	KO redundancy ~ watemp
**83**	0.009	0.286	1.000	KO redundancy ~ watemp
**84**	0.017	0.221	1.000	KO redundancy ~ watemp
**85**	0.021	0.223	1.000	KO redundancy ~ watemp
**86**	0.009	0.289	1.000	KO redundancy ~ watemp
**87**	0.017	0.291	1.000	KO redundancy ~ watemp
**88**	0.010	0.289	1.000	KO redundancy ~ watemp

**89**	0.035	0.286	1.000	KO redundancy ~ watemp
**90**	0.046	0.289	1.000	KO redundancy ~ watemp
**91**	0.043	0.240	1.000	KO redundancy ~ watemp
**92**	0.043	0.291	1.000	KO redundancy ~ watemp
**93**	0.046	0.288	1.000	KO redundancy ~ watemp
**94**	0.043	0.290	1.000	KO redundancy ~ watemp
**95**	0.046	0.290	1.000	KO redundancy ~ watemp
**96**	0.026	0.324	1.000	KO redundancy ~ watemp
**97**	0.035	0.273	1.000	KO redundancy ~ watemp
**98**	0.030	0.288	1.000	KO redundancy ~ watemp
**99**	0.038	0.289	1.000	KO redundancy ~ watemp

**100**	0.398	-0.021	1.000	KO redundancy ~ watemp
**101**	0.277	0.128	1.000	KO redundancy ~ watemp
**102**	0.265	0.130	1.000	KO redundancy ~ watemp
**103**	0.383	-0.015	1.000	KO redundancy ~ watemp
**104**	0.277	0.128	1.000	KO redundancy ~ watemp
**105**	0.102	0.129	1.000	KO redundancy ~ watemp
**106**	0.221	0.060	1.000	KO redundancy ~ watemp
**107**	0.060	0.135	1.000	KO redundancy ~ watemp
**108**	0.040	0.145	1.000	KO redundancy ~ watemp
**109**	0.565	0.029	1.000	KO redundancy ~ watemp
**110**	0.495	0.042	1.000	KO redundancy ~ watemp

**111**	0.114	0.110	1.000	KO redundancy ~ watemp
**112**	0.002	0.385	0.771	KO redundancy ~ watemp
**113**	0.134	0.100	1.000	KO redundancy ~ watemp
**114**	0.142	0.095	1.000	KO redundancy ~ watemp
**115**	0.398	0.012	1.000	KO redundancy ~ watemp
**116**	0.211	0.146	1.000	KO redundancy ~ watemp
**117**	0.007	0.366	1.000	KO redundancy ~ watemp
**118**	0.006	0.313	1.000	KO redundancy ~ watemp
**119**	0.001	0.327	0.509	KO redundancy ~ watemp
**120**	0.583	0.100	1.000	KO redundancy ~ watemp
**121**	0.583	0.100	1.000	KO redundancy ~ watemp

**122**	0.265	0.092	1.000	KO redundancy ~ watemp
**123**	0.114	0.266	1.000	KO redundancy ~ watemp
**124**	0.529	0.099	1.000	KO redundancy ~ watemp
**125**	0.547	0.099	1.000	KO redundancy ~ watemp
**126**	0.086	0.258	1.000	KO redundancy ~ watemp
**127**	0.004	0.372	1.000	KO redundancy ~ watemp
**128**	0.947	0.088	1.000	KO redundancy ~ watemp
**129**	0.947	0.088	1.000	KO redundancy ~ watemp
**130**	0.841	0.031	1.000	KO redundancy ~ watemp
**131**	0.201	0.098	1.000	KO redundancy ~ watemp
**132**	0.060	0.271	1.000	KO redundancy ~ watemp

**133**	0.086	0.085	1.000	KO redundancy ~ watemp
**134**	0.149	0.096	1.000	KO redundancy ~ watemp
**135**	0.076	0.151	1.000	KO redundancy ~ watemp
**136**	0.461	0.132	1.000	KO redundancy ~ watemp
**137**	0.033	0.202	1.000	KO redundancy ~ watemp
**138**	0.779	0.025	1.000	KO redundancy ~ watemp
**139**	0.779	0.025	1.000	KO redundancy ~ watemp
**140**	0.640	-0.022	1.000	KO redundancy ~ watemp
**141**	0.640	-0.022	1.000	KO redundancy ~ watemp
**142**	0.640	-0.022	1.000	KO redundancy ~ watemp
**143**	0.640	-0.022	1.000	KO redundancy ~ watemp

**144**	0.620	-0.022	1.000	KO redundancy ~ watemp
**145**	0.040	0.177	1.000	KO redundancy ~ watemp
**146**	0.620	-0.012	1.000	KO redundancy ~ watemp
**147**	0.013	0.199	1.000	KO redundancy ~ watemp
**148**	0.242	0.168	1.000	KO redundancy ~ watemp
**149**	0.640	-0.012	1.000	KO redundancy ~ watemp
**150**	0.841	-0.049	1.000	KO redundancy ~ watemp
**151**	0.841	-0.049	1.000	KO redundancy ~ watemp
**152**	0.841	-0.049	1.000	KO redundancy ~ watemp
**153**	0.253	0.169	1.000	KO redundancy ~ watemp
**154**	0.883	-0.050	1.000	KO redundancy ~ watemp

**155**	0.026	0.240	1.000	KO redundancy ~ watemp
**156**	0.231	0.064	1.000	KO redundancy ~ watemp
**157**	0.718	-0.065	1.000	KO redundancy ~ watemp
**158**	0.718	-0.064	1.000	KO redundancy ~ watemp
**159**	0.718	-0.064	1.000	KO redundancy ~ watemp
**160**	0.081	0.194	1.000	KO redundancy ~ watemp
**161**	0.779	-0.030	1.000	KO redundancy ~ watemp
**162**	0.221	0.153	1.000	KO redundancy ~ watemp
**163**	0.862	-0.076	1.000	KO redundancy ~ watemp
**164**	0.862	-0.076	1.000	KO redundancy ~ watemp
**165**	0.862	-0.075	1.000	KO redundancy ~ watemp

**166**	0.862	-0.075	1.000	KO redundancy ~ watemp
**167**	0.904	-0.078	1.000	KO redundancy ~ watemp
**168**	0.242	0.162	1.000	KO redundancy ~ watemp
**169**	0.102	0.169	1.000	KO redundancy ~ watemp
**170**	0.063	0.260	1.000	KO redundancy ~ watemp
**171**	0.414	0.114	1.000	KO redundancy ~ watemp
**172**	0.149	0.116	1.000	KO redundancy ~ watemp
**173**	0.414	0.114	1.000	KO redundancy ~ watemp
**174**	0.414	0.115	1.000	KO redundancy ~ watemp
**175**	0.414	0.114	1.000	KO redundancy ~ watemp
**176**	0.149	0.115	1.000	KO redundancy ~ watemp

**177**	0.414	0.114	1.000	KO redundancy ~ watemp
**178**	0.149	0.114	1.000	KO redundancy ~ watemp
**179**	0.461	0.010	1.000	KO redundancy ~ watemp
**180**	0.383	0.127	1.000	KO redundancy ~ watemp
**181**	0.383	0.127	1.000	KO redundancy ~ watemp
**182**	0.383	0.127	1.000	KO redundancy ~ watemp
**183**	0.383	0.127	1.000	KO redundancy ~ watemp
**184**	0.429	-0.126	1.000	KO redundancy ~ watemp
**185**	0.142	0.134	1.000	KO redundancy ~ watemp
**186**	0.314	0.093	1.000	KO redundancy ~ watemp
**187**	0.369	0.168	1.000	KO redundancy ~ watemp

**188**	0.149	0.119	1.000	KO redundancy ~ watemp
**189**	0.429	0.165	1.000	KO redundancy ~ watemp
**190**	0.242	0.266	1.000	KO redundancy ~ watemp
**191**	0.174	0.122	1.000	KO redundancy ~ watemp
**192**	0.265	0.138	1.000	KO redundancy ~ watemp
**193**	0.114	0.086	1.000	KO redundancy ~ watemp
**194**	0.277	0.127	1.000	KO redundancy ~ watemp
**195**	0.052	0.171	1.000	KO redundancy ~ watemp
**196**	0.314	0.138	1.000	KO redundancy ~ watemp
**197**	0.165	0.066	1.000	KO redundancy ~ watemp
**198**	0.010	0.184	1.000	KO redundancy ~ watemp

**199**	0.081	0.117	1.000	KO redundancy ~ watemp
**200**	0.221	0.075	1.000	KO redundancy ~ watemp
**201**	0.149	0.109	1.000	KO redundancy ~ watemp
**202**	0.157	0.127	1.000	KO redundancy ~ watemp
**203**	0.221	0.176	1.000	KO redundancy ~ watemp
**204**	0.221	0.099	1.000	KO redundancy ~ watemp
**205**	0.414	0.044	1.000	KO redundancy ~ watemp
**206**	0.495	0.034	1.000	KO redundancy ~ watemp
**207**	0.012	0.187	1.000	KO redundancy ~ watemp
**208**	0.565	-0.008	1.000	KO redundancy ~ watemp
**209**	0.127	0.097	1.000	KO redundancy ~ watemp

**210**	0.174	0.125	1.000	KO redundancy ~ watemp
**211**	0.211	0.069	1.000	KO redundancy ~ watemp
**212**	0.211	0.069	1.000	KO redundancy ~ watemp
**213**	0.174	0.125	1.000	KO redundancy ~ watemp
**214**	0.174	0.125	1.000	KO redundancy ~ watemp
**215**	0.253	0.005	1.000	KO redundancy ~ watemp
**216**	0.327	0.067	1.000	KO redundancy ~ watemp
**217**	0.121	0.156	1.000	KO redundancy ~ watemp
**218**	0.429	0.014	1.000	KO redundancy ~ watemp
**219**	0.192	0.154	1.000	KO redundancy ~ watemp
**220**	0.231	0.126	1.000	KO redundancy ~ watemp

**221**	0.221	0.131	1.000	KO redundancy ~ watemp
**222**	0.221	0.130	1.000	KO redundancy ~ watemp
**223**	0.221	0.128	1.000	KO redundancy ~ watemp
**224**	0.221	0.127	1.000	KO redundancy ~ watemp
**225**	0.678	-0.025	1.000	KO redundancy ~ watemp
**226**	0.174	0.100	1.000	KO redundancy ~ watemp
**227**	0.004	0.312	1.000	KO redundancy ~ watemp
**228**	0.127	0.194	1.000	KO redundancy ~ watemp
**229**	0.108	0.199	1.000	KO redundancy ~ watemp
**230**	0.183	0.170	1.000	KO redundancy ~ watemp
**231**	0.183	0.169	1.000	KO redundancy ~ watemp

**232**	0.068	0.170	1.000	KO redundancy ~ watemp
**233**	0.398	0.040	1.000	KO redundancy ~ watemp
**234**	0.052	0.178	1.000	KO redundancy ~ watemp
**235**	0.383	0.028	1.000	KO redundancy ~ watemp
**236**	0.369	0.029	1.000	KO redundancy ~ watemp
**237**	0.142	0.168	1.000	KO redundancy ~ watemp
**238**	0.369	-0.006	1.000	KO redundancy ~ watemp
**239**	0.242	0.105	1.000	KO redundancy ~ watemp
**240**	0.461	0.040	1.000	KO redundancy ~ watemp
**241**	0.478	0.038	1.000	KO redundancy ~ watemp
**242**	0.478	0.039	1.000	KO redundancy ~ watemp

**243**	0.478	0.039	1.000	KO redundancy ~ watemp
**244**	0.165	0.037	1.000	KO redundancy ~ watemp
**245**	0.063	0.214	1.000	KO redundancy ~ watemp
**246**	0.086	0.175	1.000	KO redundancy ~ watemp
**247**	0.086	0.176	1.000	KO redundancy ~ watemp
**248**	0.081	0.179	1.000	KO redundancy ~ watemp
**249**	0.779	-0.043	1.000	KO redundancy ~ watemp
**250**	0.091	0.173	1.000	KO redundancy ~ watemp
**251**	0.758	-0.069	1.000	KO redundancy ~ watemp
**252**	0.461	0.024	1.000	KO redundancy ~ watemp
**253**	0.461	0.024	1.000	KO redundancy ~ watemp

**254**	0.174	0.095	1.000	KO redundancy ~ watemp
**255**	0.157	0.042	1.000	KO redundancy ~ watemp
**256**	0.640	0.000	1.000	KO redundancy ~ watemp
**257**	0.102	0.184	1.000	KO redundancy ~ watemp
**258**	0.052	0.226	1.000	KO redundancy ~ watemp
**259**	0.678	-0.027	1.000	KO redundancy ~ watemp
**260**	0.314	0.063	1.000	KO redundancy ~ watemp
**261**	0.565	0.072	1.000	KO redundancy ~ watemp
**262**	0.231	0.152	1.000	KO redundancy ~ watemp
**263**	0.277	0.036	1.000	KO redundancy ~ watemp
**264**	0.021	0.233	1.000	KO redundancy ~ watemp

**265**	0.060	0.219	1.000	KO redundancy ~ watemp
**266**	0.327	0.114	1.000	KO redundancy ~ watemp
**267**	0.445	0.008	1.000	KO redundancy ~ watemp
**268**	0.529	0.006	1.000	KO redundancy ~ watemp
**269**	0.102	0.180	1.000	KO redundancy ~ watemp
**270**	0.341	0.057	1.000	KO redundancy ~ watemp
**271**	0.114	0.185	1.000	KO redundancy ~ watemp
**272**	0.383	0.126	1.000	KO redundancy ~ watemp
**273**	0.026	0.242	1.000	KO redundancy ~ watemp
**274**	0.076	0.224	1.000	KO redundancy ~ watemp
**275**	0.068	0.154	1.000	KO redundancy ~ watemp

**276**	0.461	0.019	1.000	KO redundancy ~ watemp
**277**	0.108	0.210	1.000	KO redundancy ~ watemp
**278**	0.108	0.212	1.000	KO redundancy ~ watemp
**279**	0.231	0.110	1.000	KO redundancy ~ watemp
**280**	0.659	-0.022	1.000	KO redundancy ~ watemp
**281**	0.063	0.119	1.000	KO redundancy ~ watemp
**282**	0.001	0.318	0.209	KO redundancy ~ watemp
**283**	0.096	0.107	1.000	KO redundancy ~ watemp
**284**	0.547	0.012	1.000	KO redundancy ~ watemp
**285**	0.414	0.050	1.000	KO redundancy ~ watemp
**286**	0.383	0.061	1.000	KO redundancy ~ watemp

**287**	0.383	0.064	1.000	KO redundancy ~ watemp
**288**	0.565	0.015	1.000	KO redundancy ~ watemp
**289**	0.015	0.239	1.000	KO redundancy ~ watemp
**290**	0.221	0.099	1.000	KO redundancy ~ watemp
**291**	0.211	0.162	1.000	KO redundancy ~ watemp
**292**	0.211	0.162	1.000	KO redundancy ~ watemp
**293**	0.211	0.162	1.000	KO redundancy ~ watemp
**294**	0.211	0.161	1.000	KO redundancy ~ watemp
**295**	0.006	0.215	1.000	KO redundancy ~ watemp
**296**	0.174	0.167	1.000	KO redundancy ~ watemp
**297**	0.043	0.236	1.000	KO redundancy ~ watemp

**298**	0.242	0.135	1.000	KO redundancy ~ watemp
**299**	0.015	0.197	1.000	KO redundancy ~ watemp
**300**	0.414	0.141	1.000	KO redundancy ~ watemp
**301**	0.461	0.109	1.000	KO redundancy ~ watemp
**302**	0.355	0.132	1.000	KO redundancy ~ watemp
**303**	0.265	0.127	1.000	KO redundancy ~ watemp
**304**	0.174	0.255	1.000	KO redundancy ~ watemp
**305**	0.369	0.021	1.000	KO redundancy ~ watemp
**306**	0.004	0.198	1.000	KO redundancy ~ watemp
**307**	0.327	0.246	1.000	KO redundancy ~ watemp
**308**	0.011	0.243	1.000	KO redundancy ~ watemp

**309**	0.314	0.049	1.000	KO redundancy ~ watemp
**310**	0.035	0.249	1.000	KO redundancy ~ watemp
**311**	0.253	0.164	1.000	KO redundancy ~ watemp
**312**	0.253	0.164	1.000	KO redundancy ~ watemp
**313**	0.174	0.184	1.000	KO redundancy ~ watemp
**314**	0.000	0.550	0.001	KO redundancy ~ watemp
**315**	0.046	0.257	1.000	KO redundancy ~ watemp
**316**	0.046	0.257	1.000	KO redundancy ~ watemp
**317**	0.033	0.299	1.000	KO redundancy ~ watemp
**318**	0.003	0.354	1.000	KO redundancy ~ watemp
**319**	0.096	0.242	1.000	KO redundancy ~ watemp

**320**	0.004	0.258	1.000	KO redundancy ~ watemp
**321**	0.277	0.171	1.000	KO redundancy ~ watemp
**322**	0.277	0.162	1.000	KO redundancy ~ watemp
**323**	0.091	0.242	1.000	KO redundancy ~ watemp
**324**	0.277	0.163	1.000	KO redundancy ~ watemp
**325**	0.277	0.163	1.000	KO redundancy ~ watemp
**326**	0.004	0.366	1.000	KO redundancy ~ watemp
**327**	0.121	0.202	1.000	KO redundancy ~ watemp
**328**	0.121	0.202	1.000	KO redundancy ~ watemp
**329**	0.020	0.248	1.000	KO redundancy ~ watemp
**330**	0.183	0.178	1.000	KO redundancy ~ watemp

**331**	0.006	0.367	1.000	KO redundancy ~ watemp
**332**	0.001	0.275	0.329	KO redundancy ~ watemp
**333**	0.211	0.091	1.000	KO redundancy ~ watemp
**334**	0.231	0.076	1.000	KO redundancy ~ watemp
**335**	0.049	0.266	1.000	KO redundancy ~ watemp
**336**	0.277	0.161	1.000	KO redundancy ~ watemp
**337**	0.001	0.324	0.509	KO redundancy ~ watemp
**338**	0.038	0.235	1.000	KO redundancy ~ watemp
**339**	0.201	0.146	1.000	KO redundancy ~ watemp
**340**	0.201	0.146	1.000	KO redundancy ~ watemp
**341**	0.017	0.225	1.000	KO redundancy ~ watemp

**342**	0.174	0.222	1.000	KO redundancy ~ watemp
**343**	0.142	0.132	1.000	KO redundancy ~ watemp
**344**	0.023	0.182	1.000	KO redundancy ~ watemp
**345**	0.121	0.133	1.000	KO redundancy ~ watemp
**346**	0.121	0.136	1.000	KO redundancy ~ watemp
**347**	0.121	0.133	1.000	KO redundancy ~ watemp
**348**	0.002	0.278	0.628	KO redundancy ~ watemp
**349**	0.242	0.132	1.000	KO redundancy ~ watemp
**350**	0.068	0.207	1.000	KO redundancy ~ watemp
**351**	0.369	0.222	1.000	KO redundancy ~ watemp
**352**	0.068	0.207	1.000	KO redundancy ~ watemp

**353**	0.086	0.277	1.000	KO redundancy ~ watemp
**354**	0.002	0.339	0.943	KO redundancy ~ watemp
**355**	0.301	0.120	1.000	KO redundancy ~ watemp
**356**	0.086	0.259	1.000	KO redundancy ~ watemp
**357**	0.015	0.270	1.000	KO redundancy ~ watemp
**358**	0.127	0.228	1.000	KO redundancy ~ watemp
**359**	0.001	0.342	0.565	KO redundancy ~ watemp
**360**	0.001	0.346	0.410	KO redundancy ~ watemp
**361**	0.134	0.191	1.000	KO redundancy ~ watemp
**362**	0.007	0.288	1.000	KO redundancy ~ watemp
**363**	0.020	0.229	1.000	KO redundancy ~ watemp

**364**	0.003	0.293	1.000	KO redundancy ~ watemp
**365**	0.231	0.141	1.000	KO redundancy ~ watemp
**366**	0.052	0.212	1.000	KO redundancy ~ watemp
**367**	0.052	0.255	1.000	KO redundancy ~ watemp
**368**	0.301	0.140	1.000	KO redundancy ~ watemp
**369**	0.108	0.254	1.000	KO redundancy ~ watemp
**370**	0.003	0.302	1.000	KO redundancy ~ watemp
**371**	0.021	0.212	1.000	KO redundancy ~ watemp
**372**	0.003	0.297	1.000	KO redundancy ~ watemp
**373**	0.086	0.143	1.000	KO redundancy ~ watemp
**374**	0.072	0.326	1.000	KO redundancy ~ watemp

**375**	0.076	0.127	1.000	KO redundancy ~ watemp
**376**	0.072	0.305	1.000	KO redundancy ~ watemp
**377**	0.072	0.306	1.000	KO redundancy ~ watemp
**378**	0.072	0.308	1.000	KO redundancy ~ watemp
**379**	0.253	0.063	1.000	KO redundancy ~ watemp
**380**	0.063	0.327	1.000	KO redundancy ~ watemp
**381**	0.017	0.267	1.000	KO redundancy ~ watemp
**382**	0.253	0.100	1.000	KO redundancy ~ watemp
**383**	0.192	0.122	1.000	KO redundancy ~ watemp
**384**	0.010	0.333	1.000	KO redundancy ~ watemp
**385**	0.035	0.139	1.000	KO redundancy ~ watemp

**386**	0.355	0.084	1.000	KO redundancy ~ watemp
**387**	0.006	0.280	1.000	KO redundancy ~ watemp
**388**	0.060	0.159	1.000	KO redundancy ~ watemp
**389**	0.081	0.193	1.000	KO redundancy ~ watemp
**390**	0.081	0.179	1.000	KO redundancy ~ watemp
**391**	0.081	0.296	1.000	KO redundancy ~ watemp
**392**	0.063	0.167	1.000	KO redundancy ~ watemp
**393**	0.005	0.224	1.000	KO redundancy ~ watemp
**394**	0.289	-0.220	1.000	KO redundancy ~ watemp

Supplementary Table 4 Leave-one-cluster-out tests output for the models with chlorophyll-a as covariate at a relative phylogenetic depth of 0.6. cluster = identifier of the phylogenetic cluster, p = p-value of the Wilcoxon signed rank test, median_effect = relative median difference in coefficient, padj = adjusted p-value, test = relationship that was tested.

cluster	p	median_effect	padj	test
**1**	0.000	0.493	0.000	norm_size ~ chla
**2**	0.192	-0.081	1.000	norm_size ~ chla
**3**	0.640	0.028	1.000	norm_size ~ chla
**4**	0.925	0.005	1.000	norm_size ~ chla
**5**	0.134	-0.104	1.000	norm_size ~ chla
**6**	0.620	0.043	1.000	norm_size ~ chla
**7**	0.314	0.085	1.000	norm_size ~ chla
**8**	0.602	0.045	1.000	norm_size ~ chla
**9**	0.183	0.119	1.000	norm_size ~ chla
**10**	0.738	-0.019	1.000	norm_size ~ chla
**11**	0.862	-0.016	1.000	norm_size ~ chla
**12**	0.989	-0.035	1.000	norm_size ~ chla
**13**	0.947	0.035	1.000	norm_size ~ chla
**14**	0.620	0.009	1.000	norm_size ~ chla
**15**	0.718	-0.013	1.000	norm_size ~ chla
**16**	0.718	-0.014	1.000	norm_size ~ chla

**17**	0.718	-0.076	1.000	norm_size ~ chla
**18**	0.779	-0.069	1.000	norm_size ~ chla
**19**	0.820	-0.032	1.000	norm_size ~ chla
**20**	0.862	-0.059	1.000	norm_size ~ chla
**21**	0.989	-0.029	1.000	norm_size ~ chla
**22**	0.201	-0.084	1.000	norm_size ~ chla
**23**	0.547	0.118	1.000	norm_size ~ chla
**24**	0.925	0.018	1.000	norm_size ~ chla
**25**	0.862	-0.019	1.000	norm_size ~ chla
**26**	0.341	-0.084	1.000	norm_size ~ chla
**27**	0.883	0.007	1.000	norm_size ~ chla
**28**	0.043	0.150	1.000	norm_size ~ chla
**29**	0.758	-0.012	1.000	norm_size ~ chla
**30**	0.000	0.288	0.003	norm_size ~ chla
**31**	0.841	0.031	1.000	norm_size ~ chla
**32**	0.968	-0.012	1.000	norm_size ~ chla
**33**	0.841	0.004	1.000	norm_size ~ chla
**1**	0.000	0.705	0.000	norm_gene ~ chla
**2**	0.369	-0.050	1.000	norm_gene ~ chla

**3**	0.659	0.125	1.000	norm_gene ~ chla
**4**	0.758	0.134	1.000	norm_gene ~ chla
**5**	0.005	-0.173	0.169	norm_gene ~ chla
**6**	0.529	0.098	1.000	norm_gene ~ chla
**7**	0.529	0.091	1.000	norm_gene ~ chla
**8**	0.495	0.092	1.000	norm_gene ~ chla
**9**	0.355	0.147	1.000	norm_gene ~ chla
**10**	0.799	0.136	1.000	norm_gene ~ chla
**11**	0.862	0.034	1.000	norm_gene ~ chla
**12**	0.738	0.092	1.000	norm_gene ~ chla
**13**	0.718	0.139	1.000	norm_gene ~ chla
**14**	0.583	0.054	1.000	norm_gene ~ chla
**15**	0.820	0.139	1.000	norm_gene ~ chla
**16**	0.820	0.138	1.000	norm_gene ~ chla
**17**	0.904	-0.018	1.000	norm_gene ~ chla
**18**	0.841	0.038	1.000	norm_gene ~ chla
**19**	0.904	-0.018	1.000	norm_gene ~ chla
**20**	0.862	0.026	1.000	norm_gene ~ chla
**21**	0.968	0.051	1.000	norm_gene ~ chla

**22**	0.265	-0.003	1.000	norm_gene ~ chla
**23**	0.602	0.203	1.000	norm_gene ~ chla
**24**	0.820	0.092	1.000	norm_gene ~ chla
**25**	1.000	0.074	1.000	norm_gene ~ chla
**26**	0.640	-0.011	1.000	norm_gene ~ chla
**27**	0.738	0.077	1.000	norm_gene ~ chla
**28**	0.056	0.217	1.000	norm_gene ~ chla
**29**	0.583	0.085	1.000	norm_gene ~ chla
**30**	0.000	0.469	0.000	norm_gene ~ chla
**31**	0.862	0.107	1.000	norm_gene ~ chla
**32**	0.862	0.030	1.000	norm_gene ~ chla
**33**	0.758	0.088	1.000	norm_gene ~ chla
**1**	0.000	0.707	0.000	norm_trna ~ chla
**2**	0.017	-0.202	0.552	norm_trna ~ chla
**3**	0.947	-0.061	1.000	norm_trna ~ chla
**4**	0.968	-0.083	1.000	norm_trna ~ chla
**5**	0.000	-0.424	0.009	norm_trna ~ chla
**6**	0.883	-0.076	1.000	norm_trna ~ chla
**7**	0.968	-0.107	1.000	norm_trna ~ chla

**8**	1.000	-0.139	1.000	norm_trna ~ chla
**9**	0.495	-0.043	1.000	norm_trna ~ chla
**10**	0.355	-0.033	1.000	norm_trna ~ chla
**11**	0.429	0.018	1.000	norm_trna ~ chla
**12**	0.369	-0.126	1.000	norm_trna ~ chla
**13**	0.678	-0.078	1.000	norm_trna ~ chla
**14**	0.149	0.036	1.000	norm_trna ~ chla
**15**	0.355	-0.006	1.000	norm_trna ~ chla
**16**	0.355	-0.006	1.000	norm_trna ~ chla
**17**	0.738	0.016	1.000	norm_trna ~ chla
**18**	0.547	-0.099	1.000	norm_trna ~ chla
**19**	0.301	-0.141	1.000	norm_trna ~ chla
**20**	0.221	-0.108	1.000	norm_trna ~ chla
**21**	0.989	-0.056	1.000	norm_trna ~ chla
**22**	0.779	-0.109	1.000	norm_trna ~ chla
**23**	0.779	-0.075	1.000	norm_trna ~ chla
**24**	0.758	-0.098	1.000	norm_trna ~ chla
**25**	0.583	-0.006	1.000	norm_trna ~ chla
**26**	0.165	0.048	1.000	norm_trna ~ chla

**27**	0.461	-0.112	1.000	norm_trna ~ chla
**28**	0.000	0.240	0.001	norm_trna ~ chla
**29**	0.301	-0.094	1.000	norm_trna ~ chla
**30**	0.495	-0.075	1.000	norm_trna ~ chla
**31**	0.989	-0.032	1.000	norm_trna ~ chla
**32**	0.698	-0.079	1.000	norm_trna ~ chla
**33**	0.989	-0.088	1.000	norm_trna ~ chla
